# Author Correction: Arylmethylamino steroids as antiparasitic agents

**DOI:** 10.1038/s41467-019-11018-x

**Published:** 2019-07-08

**Authors:** Reimar Krieg, Esther Jortzik, Alice-Anne Goetz, Stéphanie Blandin, Sergio Wittlin, Mourad Elhabiri, Mahsa Rahbari, Selbi Nuryyeva, Kerstin Voigt, Hans-Martin Dahse, Axel Brakhage, Svenja Beckmann, Thomas Quack, Christoph G. Grevelding, Anthony B. Pinkerton, Bruno Schönecker, Jeremy Burrows, Elisabeth Davioud-Charvet, Stefan Rahlfs, Katja Becker

**Affiliations:** 1 Institute of Anatomy II, University Hospital Jena, Teichgraben 7, 07743 Jena, Germany; 2 Biochemistry and Molecular Biology, Interdisciplinary Research Centre, Justus Liebig University Giessen, Heinrich Buff Ring 26-32, 35392 Giessen, Germany; 3 Université de Strasbourg, CNRS, Inserm, MIR UPR9022/U963, F-67000 Strasbourg, France; 4 Swiss Tropical and Public Health Institute, Socinstrasse 57, PO Box, 4002 Basel, Switzerland; 5 University of Basel, Petersplatz 1, 4001 Basel, Switzerland; 6 UMR 7509 Centre National de la Recherche Scientifique and University of Strasbourg, European School of Chemistry, Polymers and Materials (ECPM), 25, rue Becquerel, F-67087 Strasbourg, France; 7 New York University Abu Dhabi, PO Box, 129188 Abu Dhabi, UAE; 8 Leibniz Institute for Natural Product Research and Infection Biology-Hans Knöll Institute (HKI), Adolf-Reichwein-Strasse 23, 07745 Jena, Germany; 9 Institute of Parasitology, Biomedical Research Centre, Justus Liebig University Giessen, Schubertstrasse 81, 35392 Giessen, Germany; 10 Conrad Prebys Center for Chemical Genomics, Sanford-Burnham-Prebys Medical Discovery Institute, 10901 North Torrey Pines Road, La Jolla, California, 92037 USA; 11 Conrad Prebys Center for Chemical Genomics, Sanford-Burnham-Prebys Medical Discovery Institute, 6400 Sanger Road, Orlando, Florida 32827 USA; 12 Institute of Organic and Macromolecular Chemistry, Friedrich Schiller University Jena, Humboldtstrasse 10, 07743 Jena, Germany; 13 Medicines for Malaria Venture, 20 Route de Pré-Bois, 1215 Geneva 15, Switzerland

Correction to: *Nature Communications* 10.1038/ncomms14478, published online 17 February 2019.

This Article contains errors in Figure 1a. The chemical structure of compound **9** is incorrect. The configurational assignments for compounds **3**, **5**, **7** and **10** are incorrectly labeled as ‘17alpha’ and should be labelled as ‘16alpha’. The configurational assignments for compounds **4**, **6**, **8** and **11** are incorrectly labeled as ‘17beta’ and should be labelled as ‘16beta’. The configurational assignments for compounds **13** and **14** are incorrectly labeled as ‘17alpha’ and ‘17beta’ and should be labelled as ‘3beta’ and ‘3alpha’, respectively. The correct version of Fig. 1a is:

**Figure 1:**
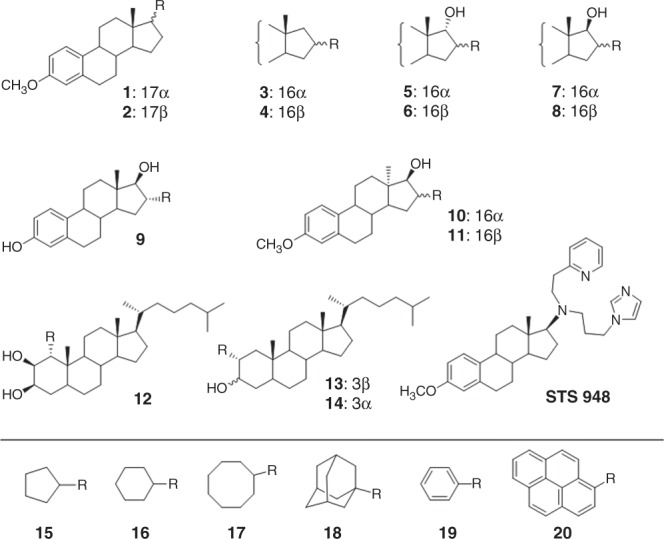
Fig. 1

The error has not been corrected in the PDF or HTML versions of the Article.

