# Supplementary material for: Arylmethylamino steroids as antiparasitic agents
Source: Nat Commun. 2017 Feb 17;8:14478. doi: 10.1038/ncomms14478 (PMC5321741; doi:10.1038/ncomms14478)
Supplement: Supplementary Information — Supplementary Figures, Supplementary Tables, Supplementary Notes, Supplementary Methods and Supplementary References [file ncomms14478-s1.pdf]

Supplementary Figures

A

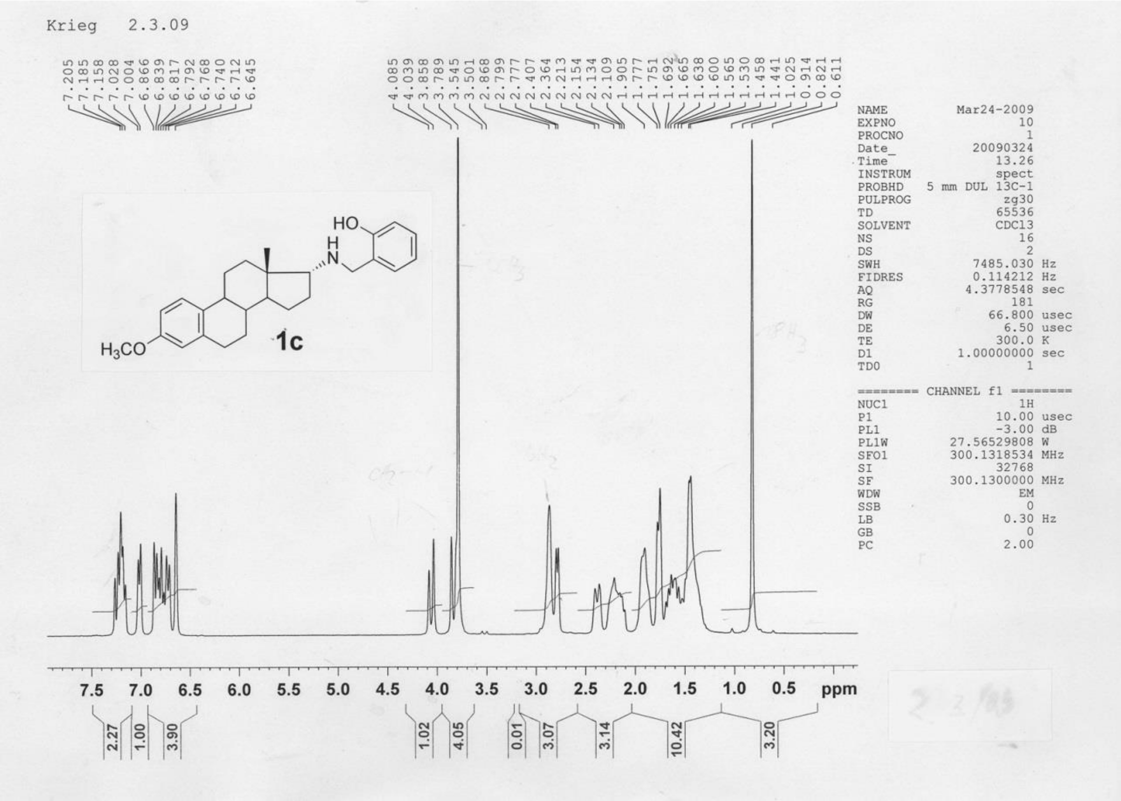

B

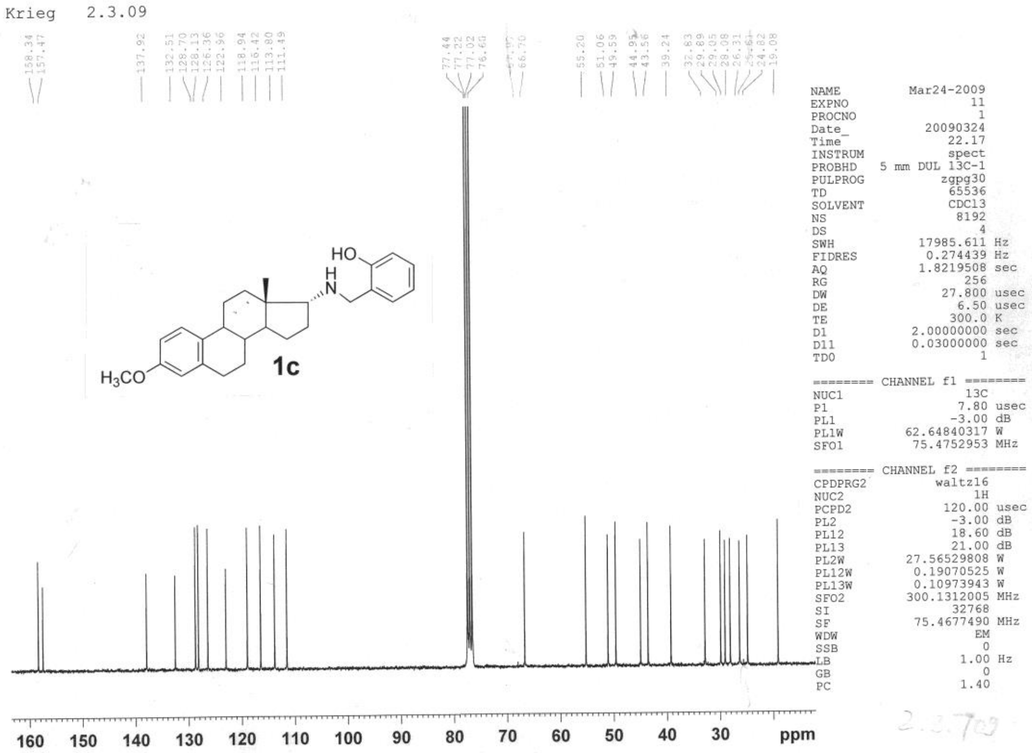

C

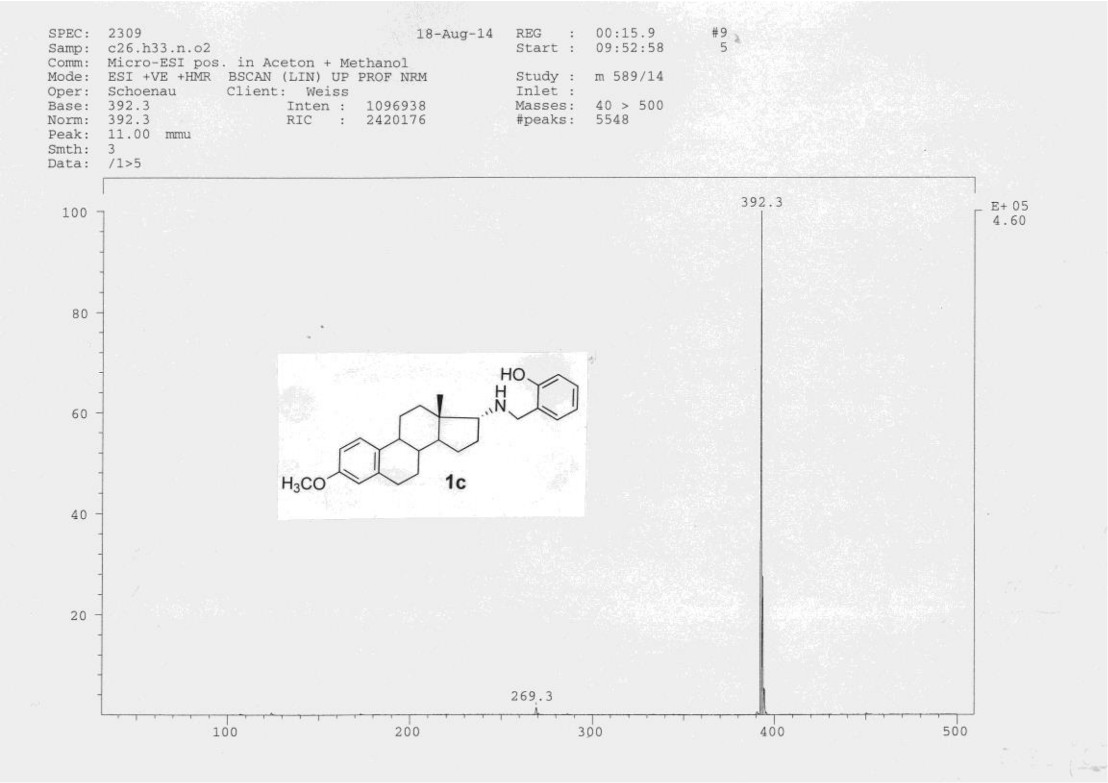

Supplementary Figure 1. (A, B, C) ^{1}H and ^{13}C NMR spectra of compound 1c.

A

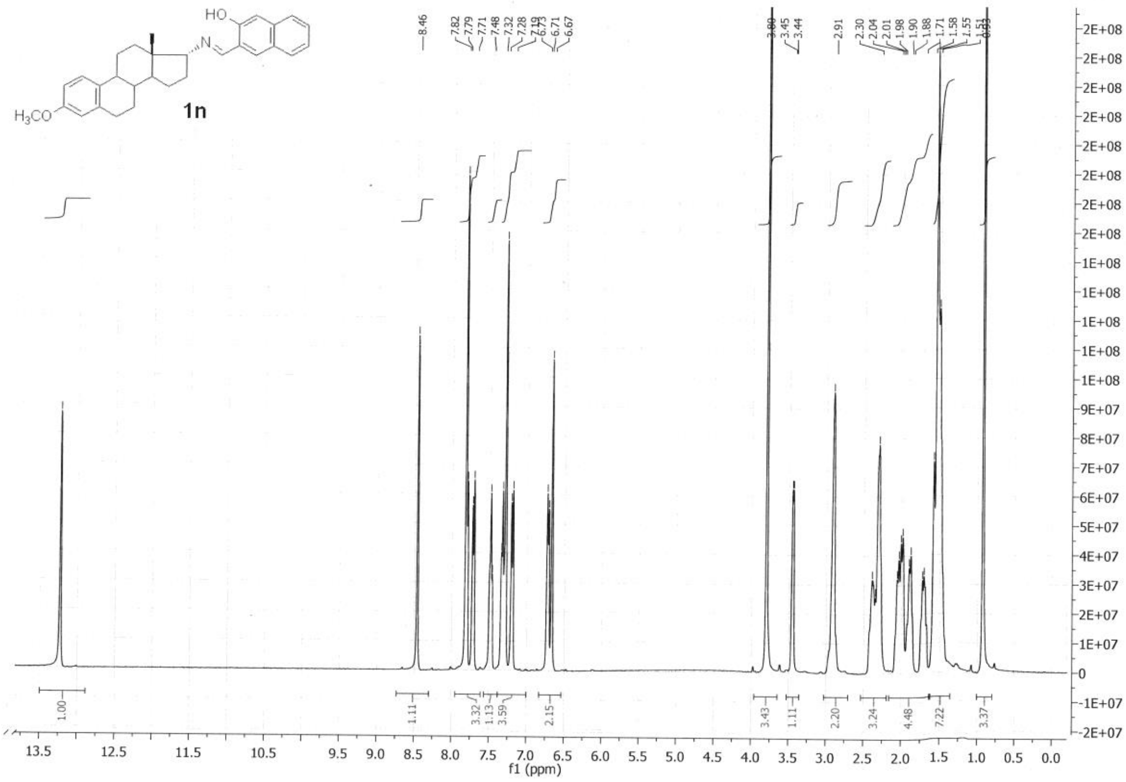

B

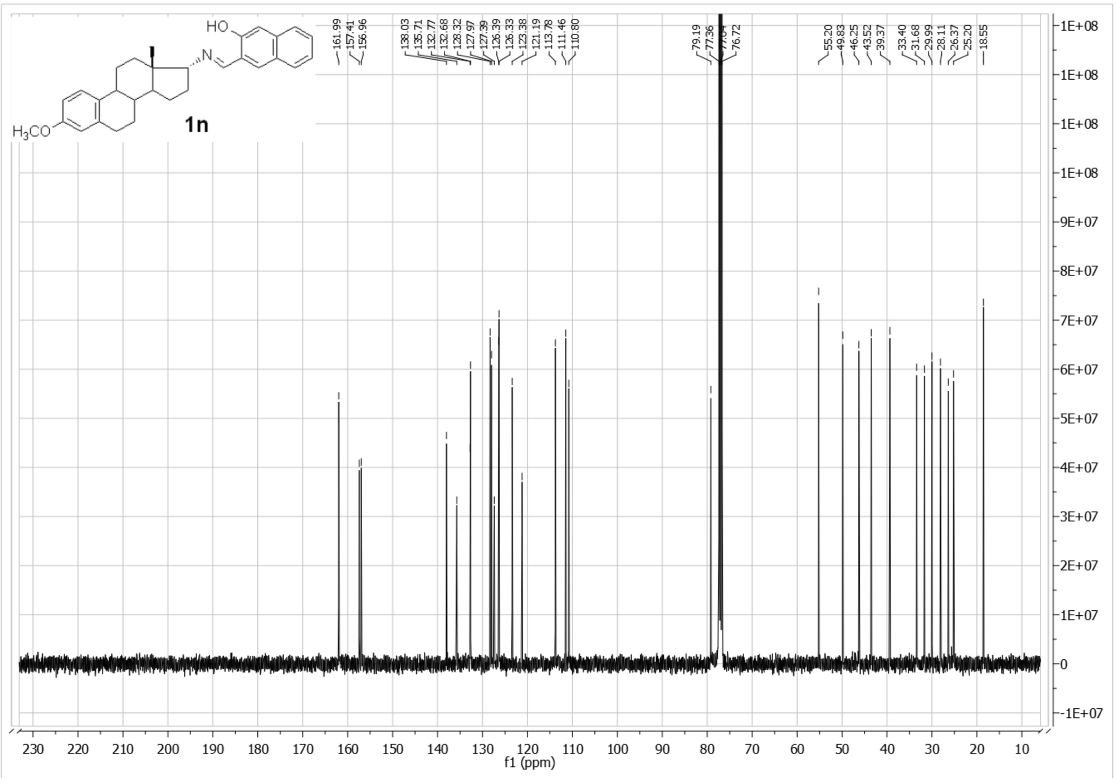

C

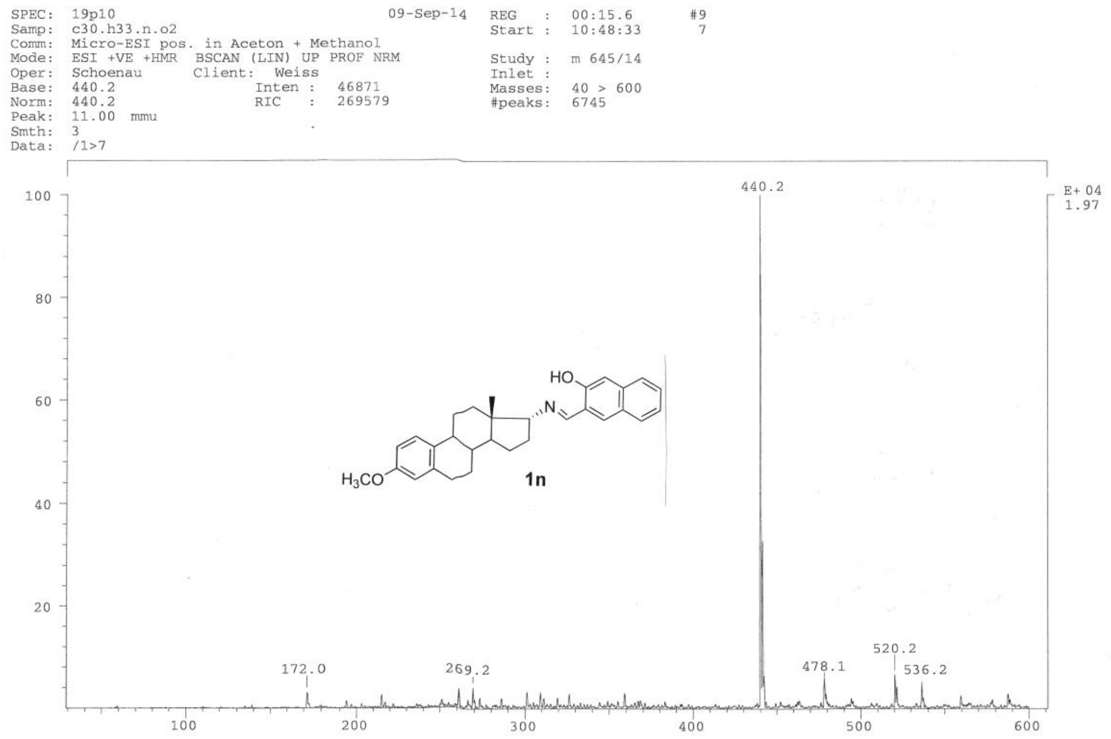

Supplementary Figure 2. (A, B, C) ^{1}H and ^{13}C NMR spectra of compound 1n.

A

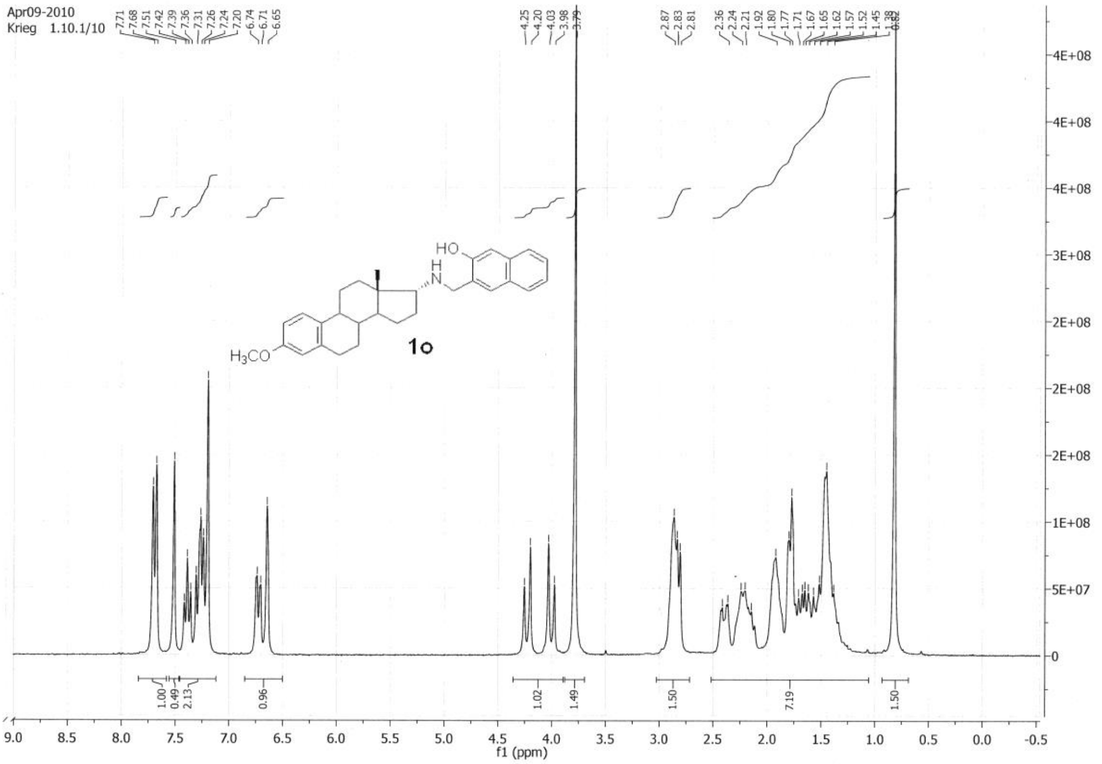

B

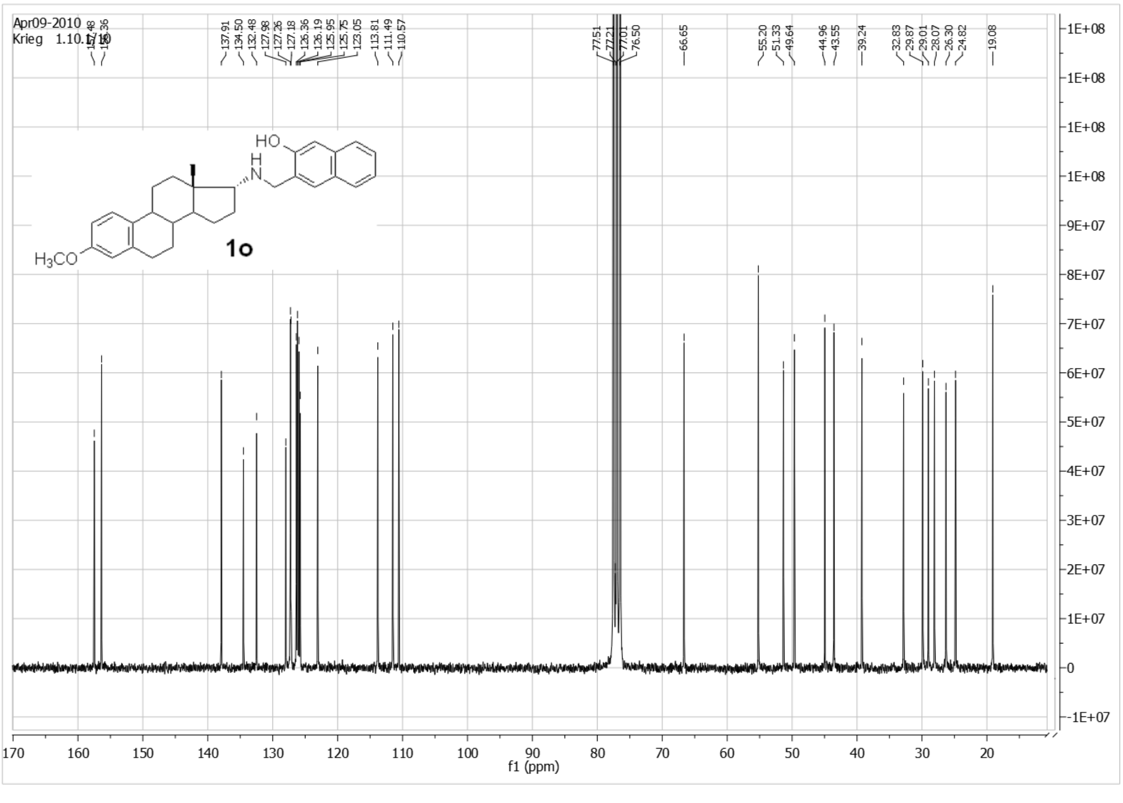

C

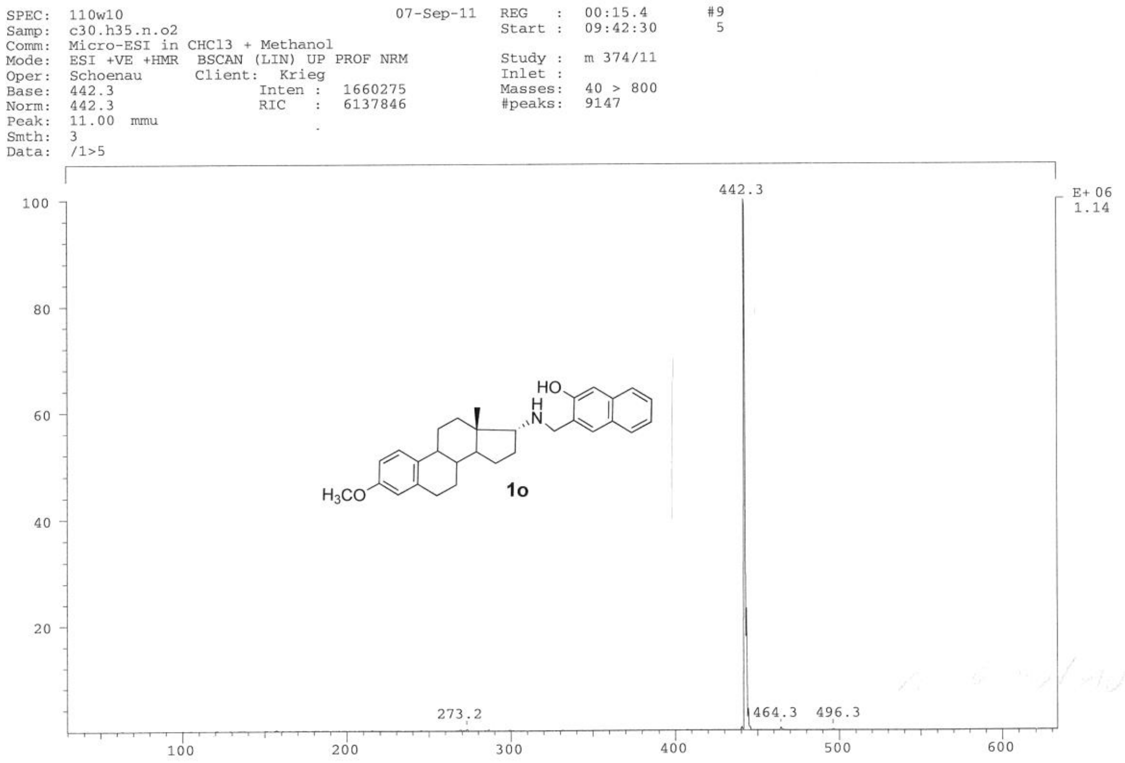

Supplementary Figure 3. (A, B, C) ^{1}H and ^{13}C NMR spectra of compound 1o.

A

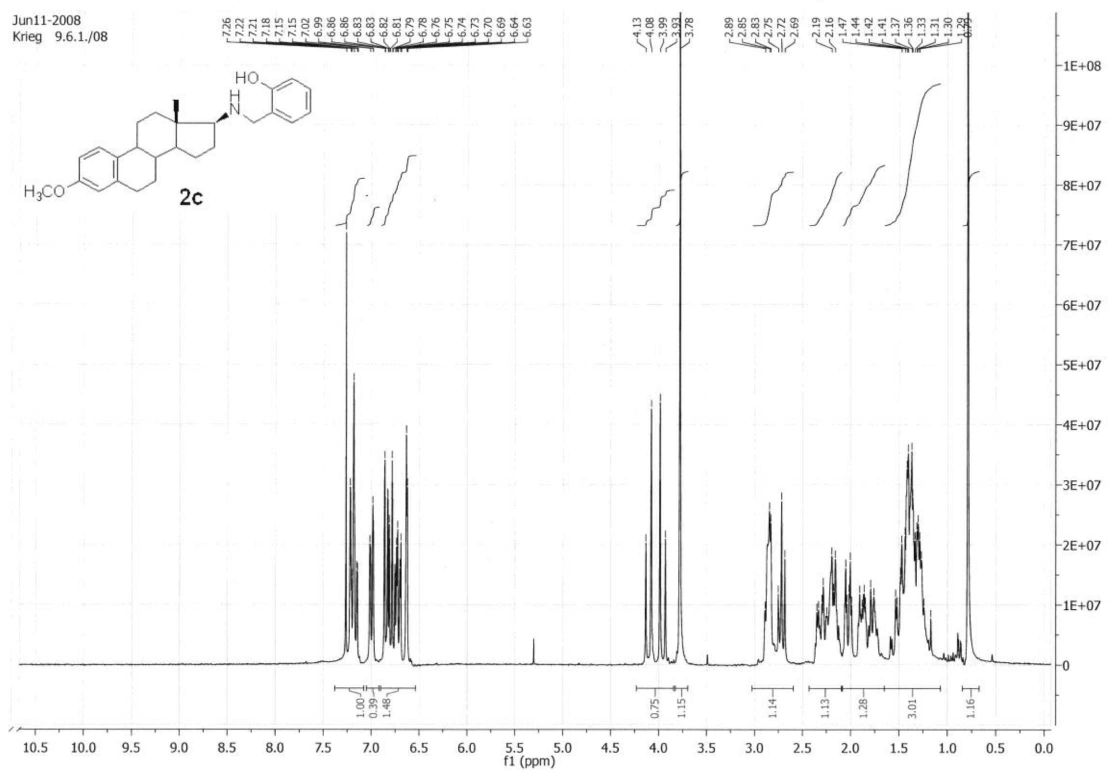
Jun11-2008
Krieg 9.6.1./08
HO
N
H
H3CO
2c
f1 (ppm)

B

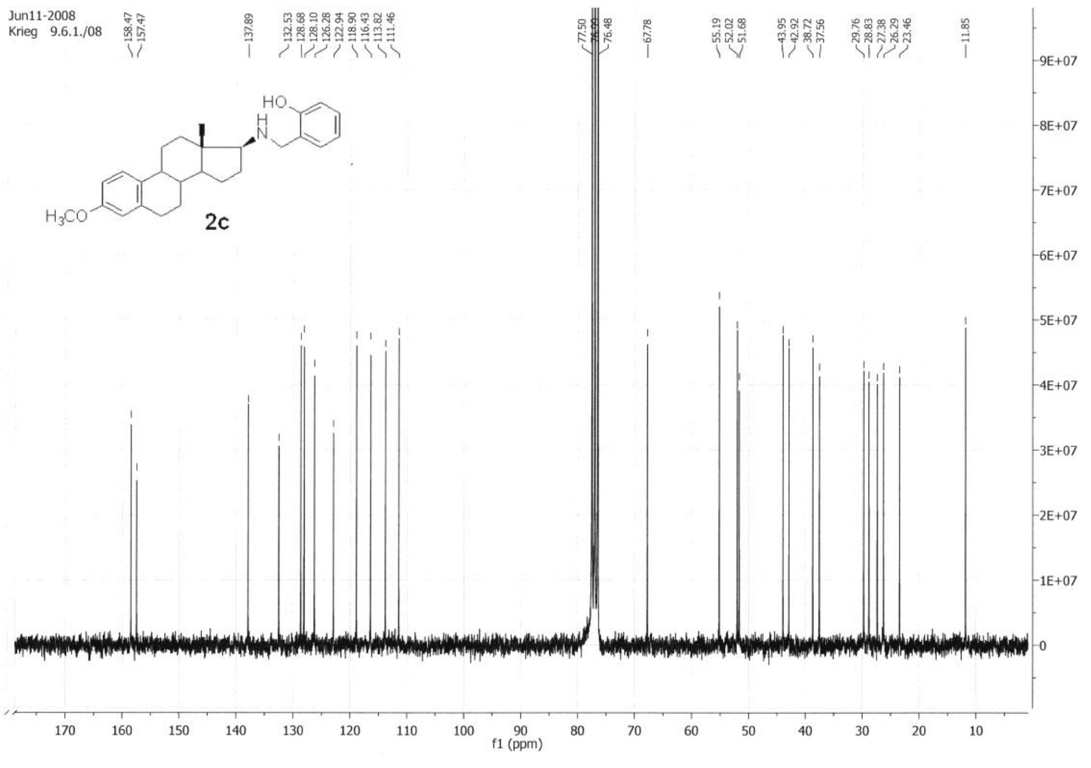
Jun11-2008
Krieg 9.6.1./08
HO
N
H
H3CO
2c
f1 (ppm)

C

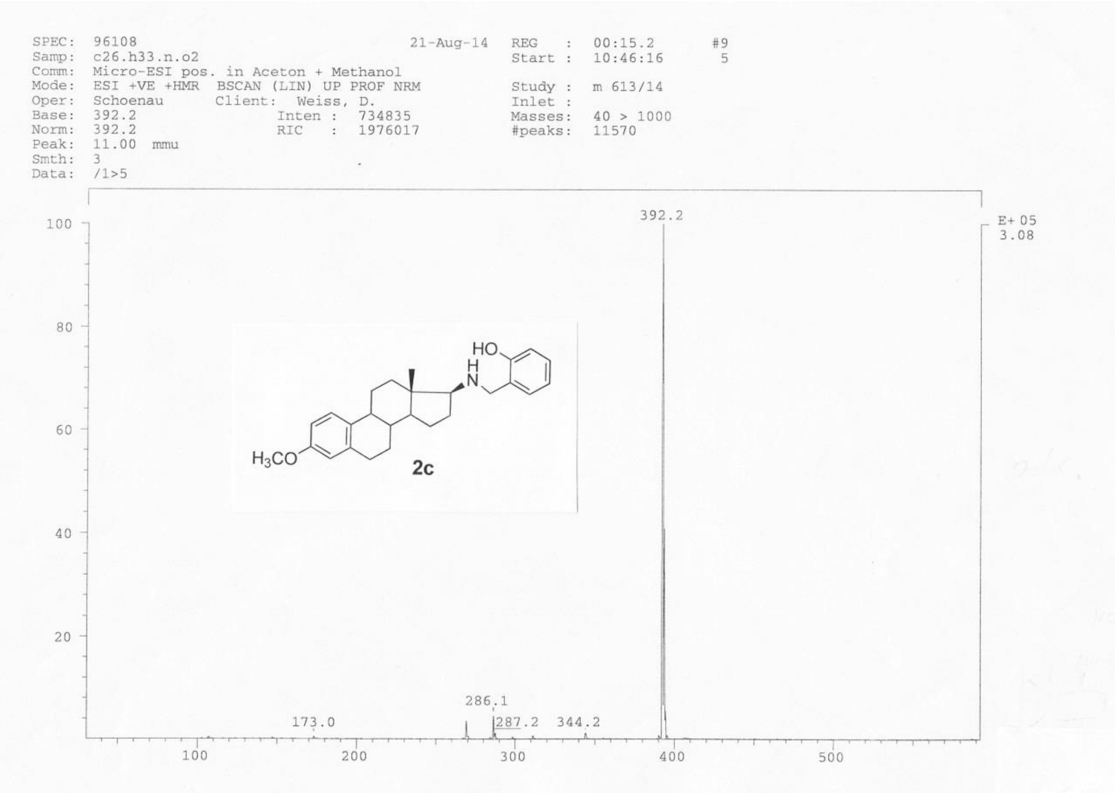

Supplementary Figure 4. (A, B, C) ^{1}H and ^{13}C NMR spectra of compound 2c.

A

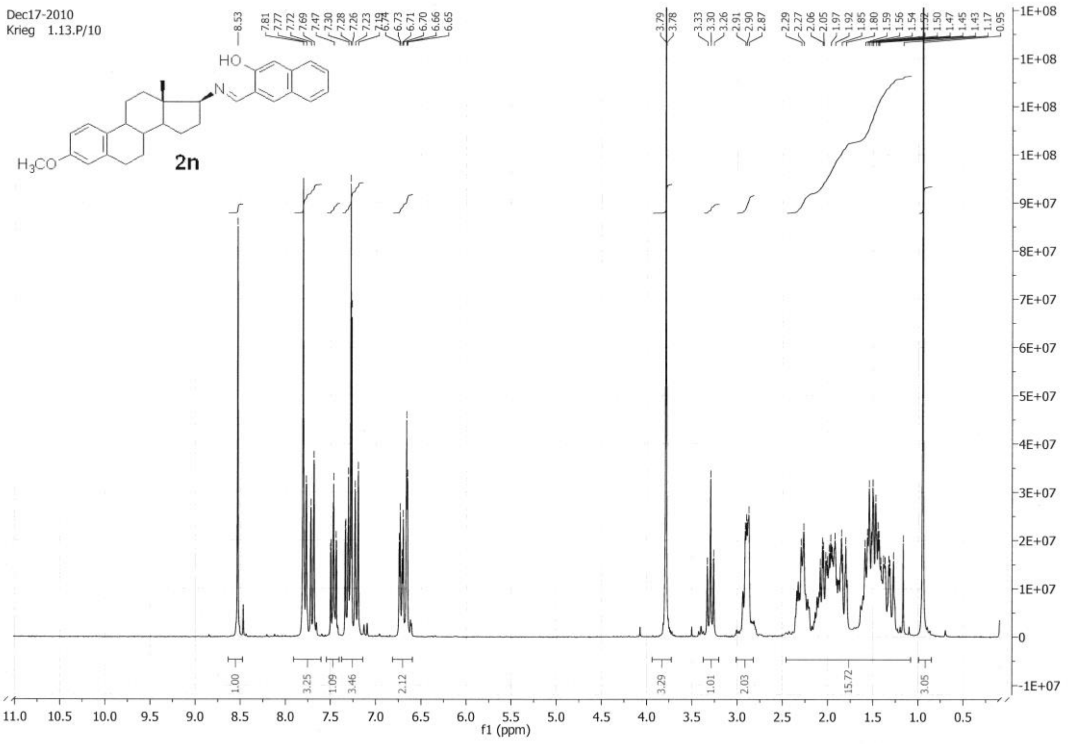

B

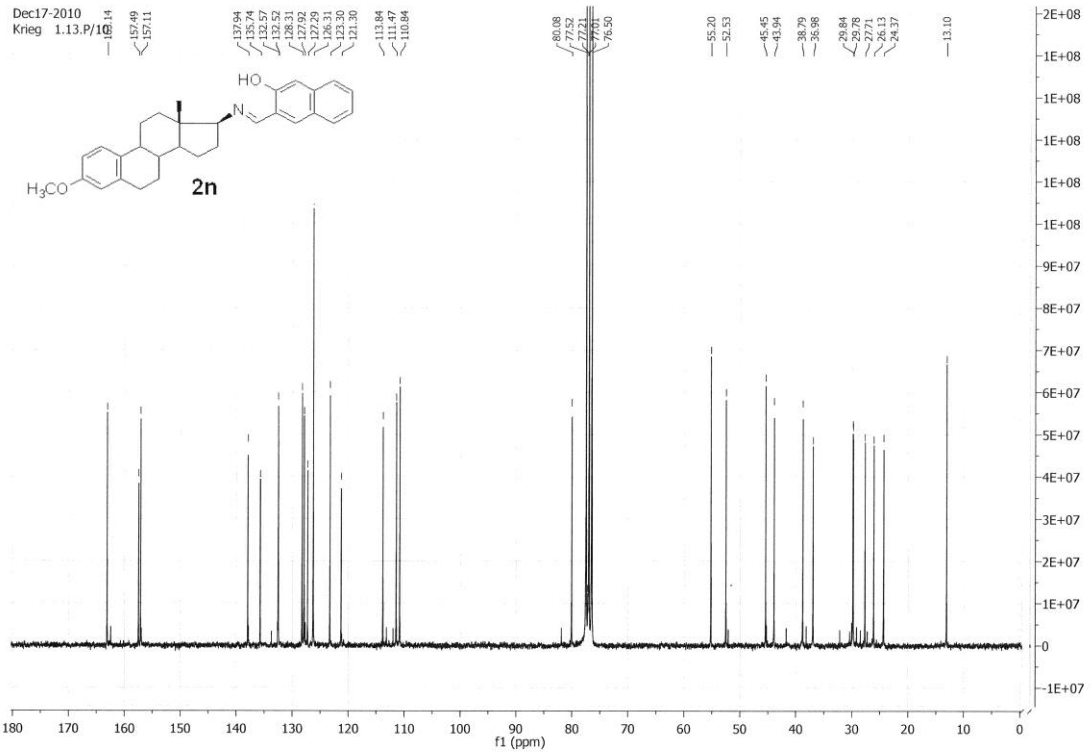

Supplementary Figure 5. (A, B) ^{1}H and ^{13}C NMR spectra of compound 2n.

A

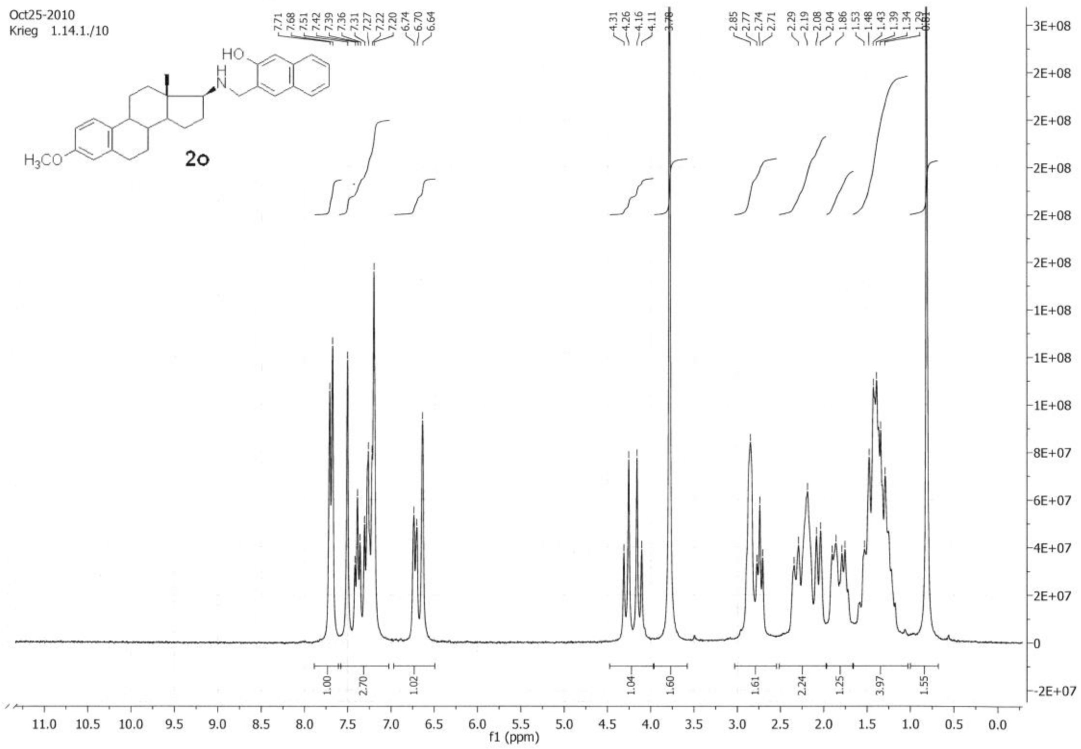
Oct25-2010
Krieg 1.14.1./10
HO
H
N
H3CO
2o
f1 (ppm)

B

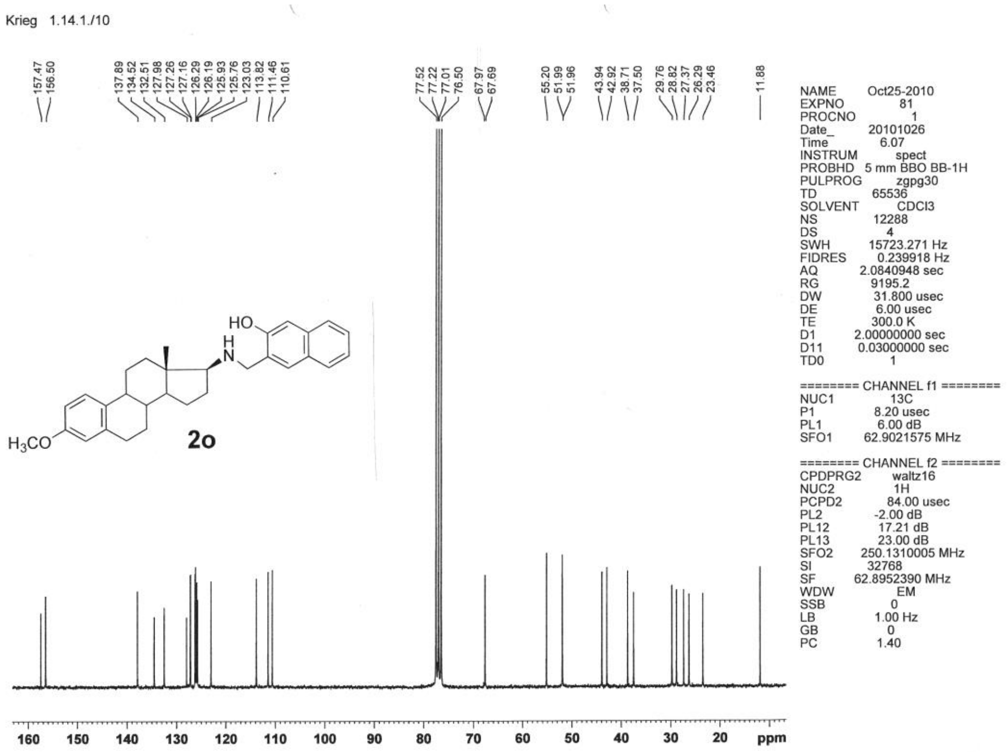
Krieg 1.14.1./10
HO
H
N
H3CO
2o
NAME Oct25-2010
EXPNO 81
PROCNO 1
Date_ 20101026
Time 6.07
INSTRUM spect
PROBHD 5 mm BBO BB-1H
PULPROG zgpg30
TD 65536
SOLVENT CDCl3
NS 12288
DS 4
SWH 15723.271 Hz
FIDRES 0.239918 Hz
AQ 2.0840948 sec
RG 9195.2
DW 31.800 usec
DE 6.00 usec
TE 300.0 K
D1 2.00000000 sec
D11 0.03000000 sec
TD0 1
======== CHANNEL f1 ========
NUC1 13C
P1 8.20 usec
PL1 6.00 dB
SFO1 62.9021575 MHz
======== CHANNEL f2 ========
CPDPRG2 waltz16
NUC2 1H
PCPD2 84.00 usec
PL2 -2.00 dB
PL12 17.21 dB
PL13 23.00 dB
SFO2 250.1310005 MHz
SI 32768
SF 62.8952390 MHz
WDW EM
SSB 0
LB 1.00 Hz
GB 0
PC 1.40
ppm

C

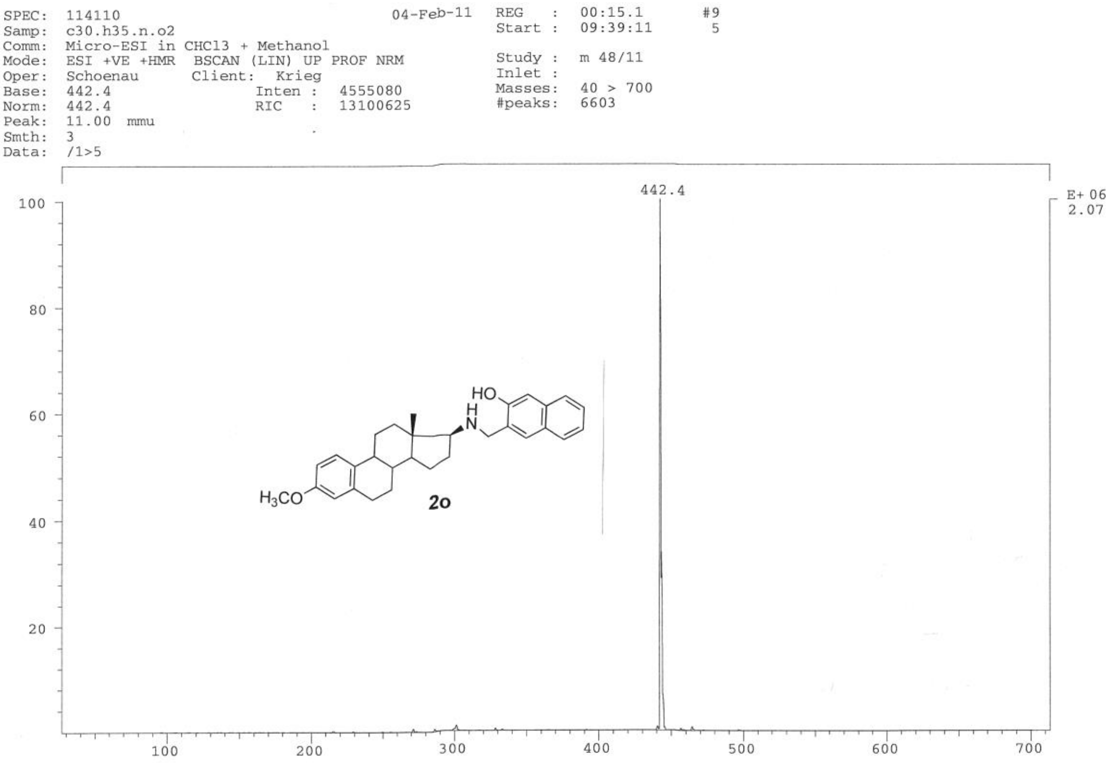

Supplementary Figure 6. (A, B, C) ^{1}H and ^{13}C NMR spectra of compound 2o.

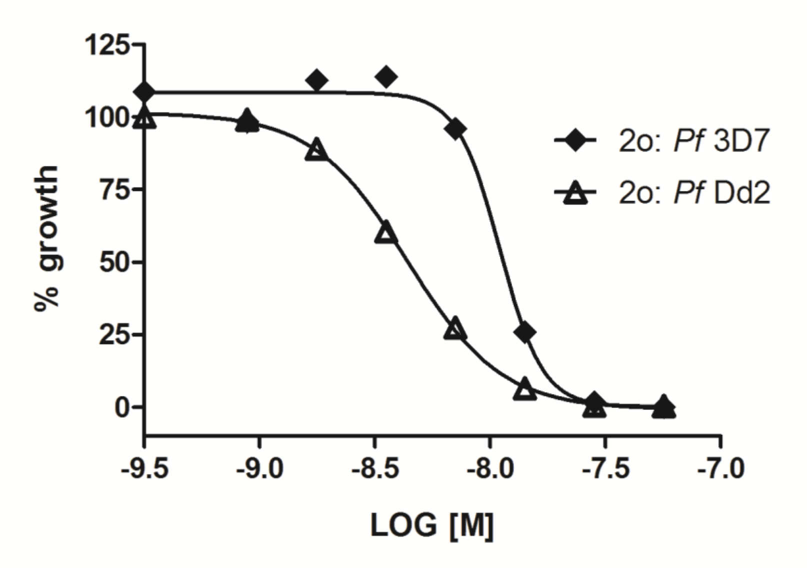

Supplementary Figure 7. IC_{50} values on chloroquine-sensitive (3D7) and resistant (Dd2) *Plasmodium falciparum* blood stage parasites shown here for compound 2o.

Representative curves are given. IC_{50} values determined to be: **1o** 3D7: 4.1 ± 1.6 nM; **2o** 3D7: 6.6 ± 2.1 nM; **1o** Dd2: 1.0 ± 0.9 nM; **2o** Dd2: 2.0 ± 1.2 nM (mean values of at least 3 independent replicates with standard deviation).

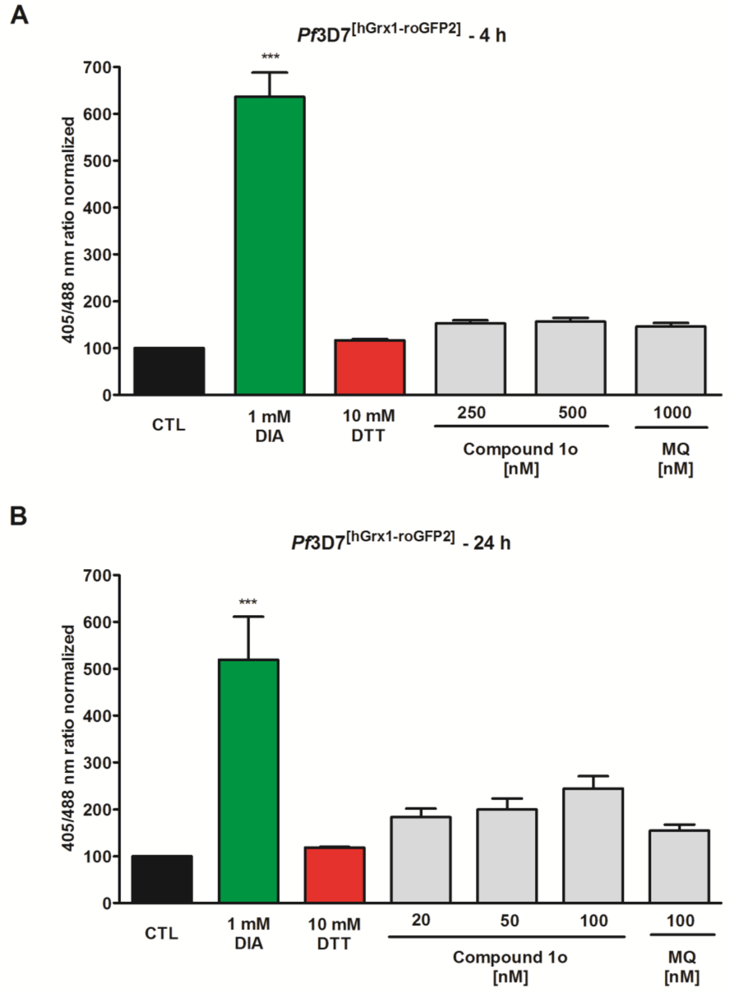

Supplementary Figure 8. Mid- and long-term effects of compound 1o on the redox ratio of *P. falciparum* 3D7[hGrx1-roGFP2] parasites. (A) *P. falciparum* cells were treated with compound **1o** (IC_{50} ~ 5 nM) at 50 x and 100 x IC_{50} and 1 µM MQ (IC_{50} = 8 nM) for 4 h. (B) For 24 h incubations, cells were treated with compound **1o** at 4 x, 10 x, 20 x IC_{50}, and with 100 nM MQ. Free thiols were subsequently blocked with 2 mM NEM. Both 4 h and 24 h incubations of 3D7[hGrx1-roGFP2] parasites with compound **1o** showed an increase of the 405/488 nm fluorescence ratio in a concentration-dependent manner. Non-treated parasites served as controls. All experiments included fully oxidized (1 mM DIA) and fully reduced (10 mM DTT) parasites (2 min incubation) prior to blocking with NEM. Data (mean ± SEM) from three independent experiments are shown. One-way ANOVA test with 95% confidence intervals with the Dunnett's Multiple Comparison Test was applied for statistical analysis of significance (*, $p < 0.05$; **, $p < 0.01$; ***, $p < 0.001$) and indicated significant changes compared to the respective control.

A

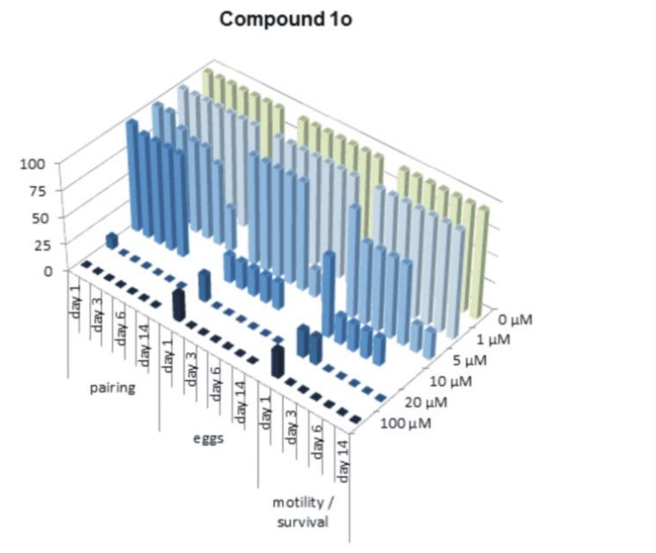
Compound 1o
100
75
50
25
0
day 1
day 3
day 6
day 14
day 1
day 3
day 6
day 14
day 1
day 3
day 6
day 14
pairing
eggs
motility / survival
0 µM
1 µM
5 µM
10 µM
20 µM
100 µM

B

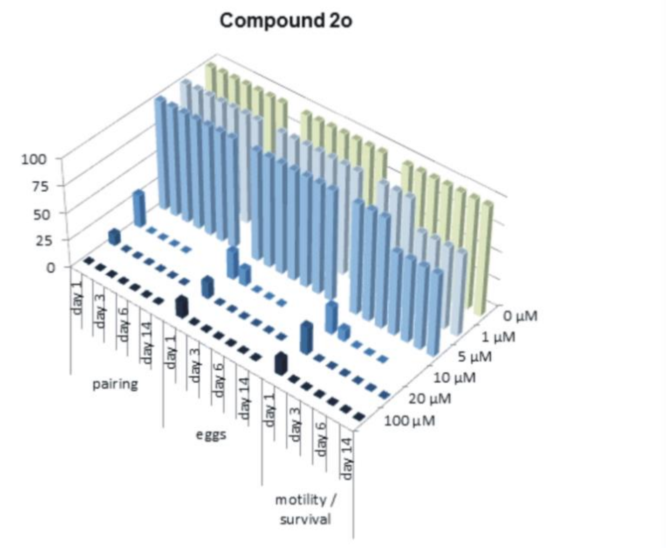
Compound 2o
100
75
50
25
0
day 1
day 3
day 6
day 14
day 1
day 3
day 6
day 14
day 1
day 3
day 6
day 14
pairing
eggs
motility / survival
0 µM
1 µM
5 µM
10 µM
20 µM
100 µM

C

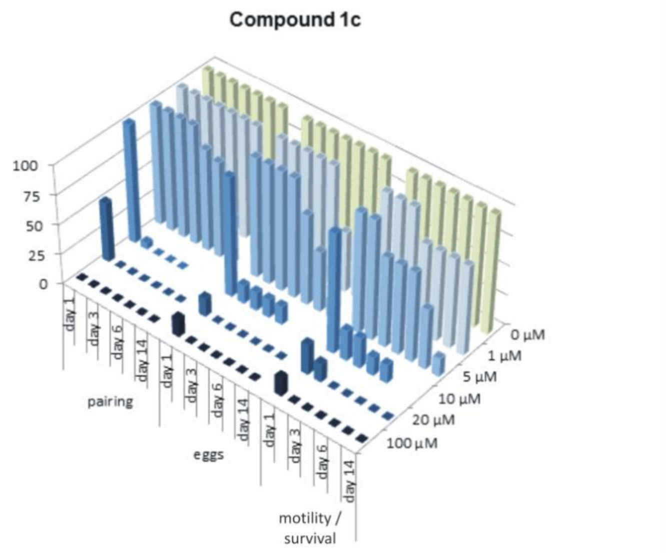
Compound 1c
100
75
50
25
0
day 1
day 3
day 6
day 14
day 1
day 3
day 6
day 14
day 1
day 3
day 6
day 14
pairing
eggs
motility / survival
0 µM
1 µM
5 µM
10 µM
20 µM
100 µM

Supplementary Figure 9. Effects of the steroid compounds on adult *Schistosoma mansoni in vitro*

Diagrams (A-C) summarize the observed effects (given as a % on the x-axis) of different concentrations (1-100 µM; 0 µM = untreated control worms) of the compounds **1o**, **2o**, and **1c** on pairing stability (pairing), egg production (eggs), and motility/survival in adult schistosomes over a treatment period of 14 days.

(A) 1o: using a concentration of 5 µM, couples started to separate from day 2 onward and showed slightly reduced motility, which diminished further with increasing treatment time. At 10 µM, egg production as well as the motility of the treated worms were remarkably reduced (about 75%) starting from days 1 and 2, respectively. At 20 µM, no paired worms were present anymore from day 2 on, egg production stopped completely, and nearly all worms were dead.

(B) 2o: using a concentration of 1-5 µM, slightly reduced motility was observed as a first sign of decreasing viability from day 4 on. Increasing the concentration to 10 µM significantly affected pairing stability, egg production, and motility/survival beginning with days 1-2 of the treatment. At this concentration, the worms died within 3 days. At 20 µM, all worms were dead after 1 day of treatment.

(C) 1c: starting with decreased motility using 1 µM from day 4 of treatment on, reduced pairing stability and egg production as well as increasingly diminished motility were observed using a concentration of 5 µM after about 1 week. These effects were enhanced at 10 µM after 2 days of treatment, leading to the death of nearly all worms at this concentration after 6 days. Survival of worms ended already after 2 days of treatment when 20 µM were applied.

A

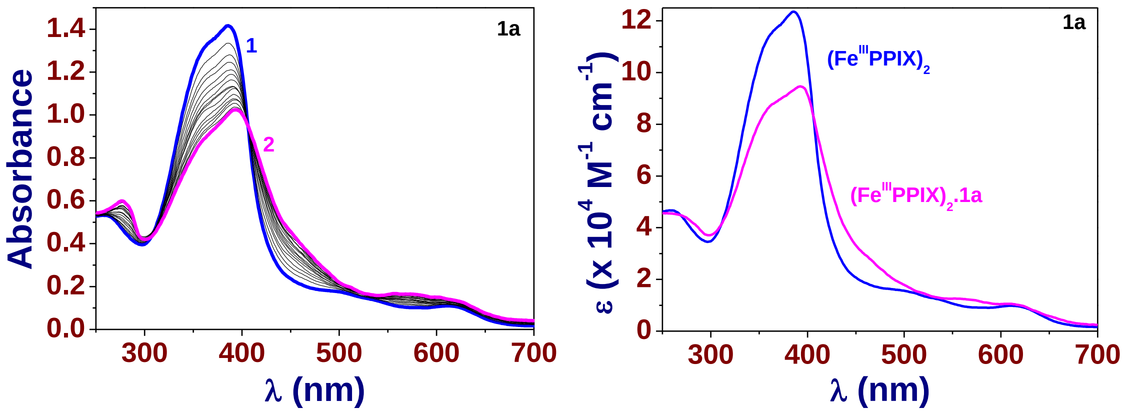
Absorbance
λ (nm)
1a
1
2
ε (x 10^4 M^-1 cm^-1)
λ (nm)
1a
(Fe^III PPIX)_2
(Fe^III PPIX)_2.1a

B

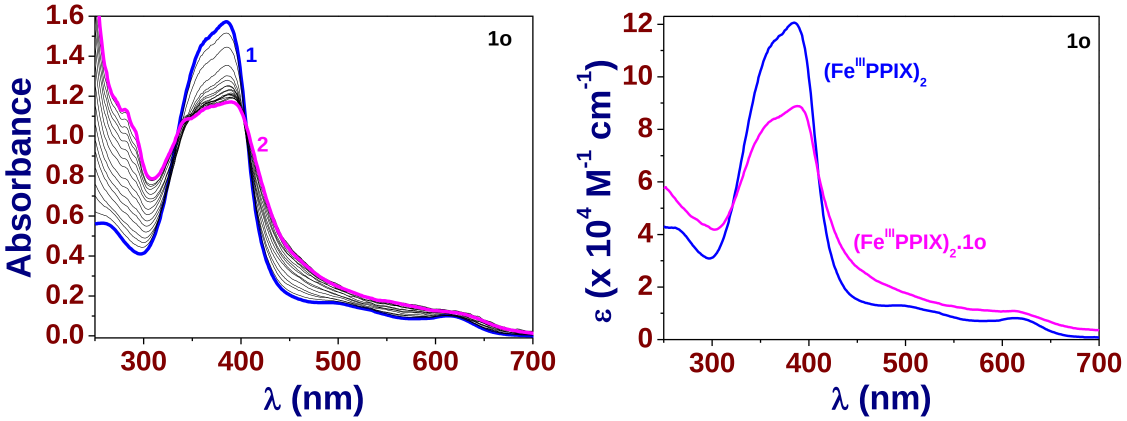
Absorbance
λ (nm)
1o
1
2
ε (x 10^4 M^-1 cm^-1)
λ (nm)
1o
(Fe^III PPIX)_2
(Fe^III PPIX)_2.1o

C

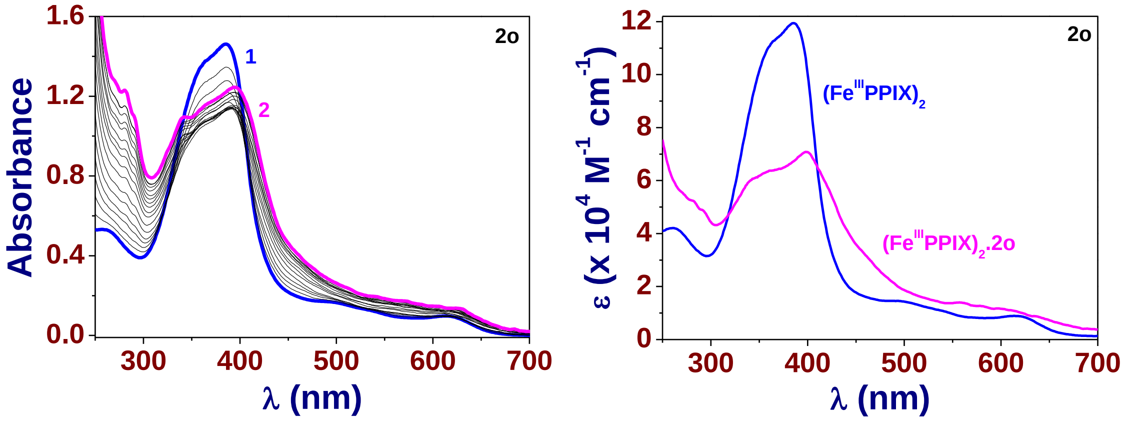
Absorbance
λ (nm)
2o
1
2
ε (x 10^4 M^-1 cm^-1)
λ (nm)
2o
(Fe^III PPIX)_2
(Fe^III PPIX)_2.2o

D

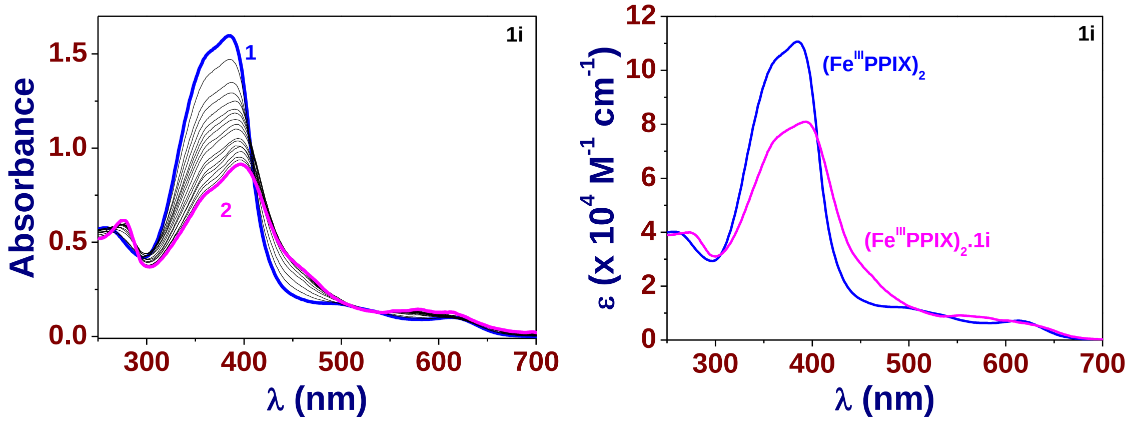
1i
Absorbance
λ (nm)
1
2
ε (x 10^4 M^-1 cm^-1)
(Fe^III PPIX)_2
(Fe^III PPIX)_2.1i

E

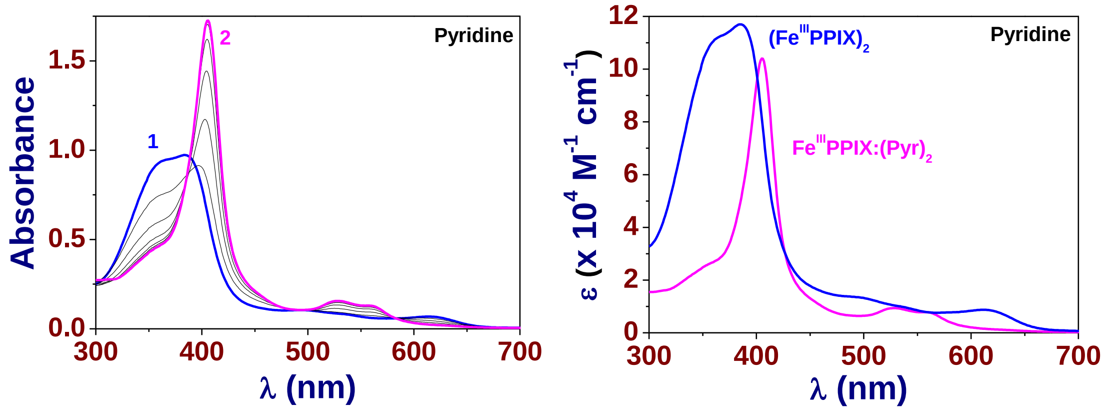
Pyridine
Absorbance
λ (nm)
1
2
ε (x 10^4 M^-1 cm^-1)
(Fe^III PPIX)_2
Fe^III PPIX:(Pyr)_2

F

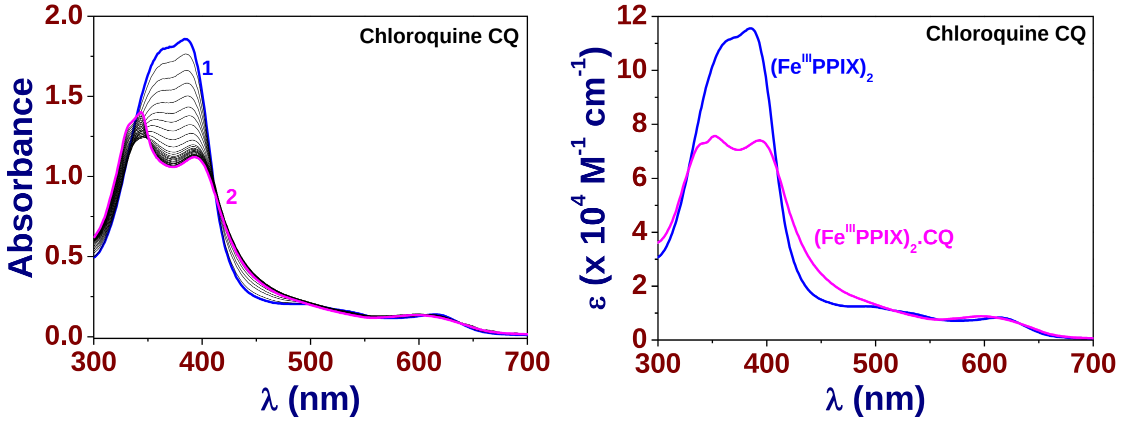
Chloroquine CQ
Absorbance
λ (nm)
1
2
ε (x 10^4 M^-1 cm^-1)
(Fe^III PPIX)_2
(Fe^III PPIX)_2.CQ

Supplementary Figure 10. (left) Spectrophotometric titration of haem (under its π-π dimeric form, log K_{Dim} = 6.82) by the substrates **1a**, **1o**, **2o**, **1i**, and chloroquine (**CQ**), and (right) electronic spectra of haem, of the free substrates and of their corresponding Fe^{III}PPIX substrate complexes. Solvent: 0.2 M aqueous sodium HEPES buffer pH 7.5; T = 25.0 °C; l = 1 cm.

For **1a**: $[Fe^{III}PPIX]_0 = 2.41 \times 10^{-5}$ M; (1) $[\mathbf{1a}]_0/[Fe^{III}PPIX]_0 = 0$; (2) $[\mathbf{1a}]_0/[Fe^{III}PPIX]_0 = 2.78$.

For **1o**: $[Fe^{III}PPIX]_0 = 2.80 \times 10^{-5}$ M; (1) $[\mathbf{1o}]_0/[Fe^{III}PPIX]_0 = 0$; (2) $[\mathbf{1o}]_0/[Fe^{III}PPIX]_0 = 2.39$.

For **2o**: $[Fe^{III}PPIX]_0 = 2.46 \times 10^{-5}$ M; (1) $[\mathbf{2o}]_0/[Fe^{III}PPIX]_0 = 0$; (2) $[\mathbf{2o}]_0/[Fe^{III}PPIX]_0 = 2.72$.

For **1i**: $[Fe^{III}PPIX]_0 = 3.0 \times 10^{-5}$ M; (1) $[\mathbf{1i}]_0/[Fe^{III}PPIX]_0 = 0$; (2) $[\mathbf{1i}]_0/[Fe^{III}PPIX]_0 = 2.23$.

For **CQ**: $[Fe^{III}PPIX]_0 = 3.36 \times 10^{-5}$ M; (1) $[\mathbf{CQ}]_0/[Fe^{III}PPIX]_0 = 0$; (2) $[\mathbf{CQ}]_0/[Fe^{III}PPIX]_0 = 1.03$.

For pyridine (noted **Pyr**), $[Fe^{III}PPIX]_0 = 1.68 \times 10^{-5}$ M; (1) $[\mathbf{Pyr}]_0/[Fe^{III}PPIX]_0 = 0$; (2) $[\mathbf{CQ}]_0/[Fe^{III}PPIX]_0 = 51667$.

(A) log $\beta_{(FeIIIPPIX)_2.\mathbf{1a}}$ = 12.0 ± 0.2; (B) log $\beta_{(FeIIIPPIX)_2.\mathbf{1o}}$ = 12.3 ± 0.2; (C) log $\beta_{(FeIIIPPIX)_2.\mathbf{2o}}$ = 11.9 ± 0.2; (D) log $\beta_{(FeIIIPPIX)_2.\mathbf{1i}}$ = 12.0 ± 0.2; (E) log $\beta(_{FeIIIPPIX)2.\mathbf{pyr}}$ = 2.25 ± 0.05; (F) log $\beta_{(FeIIIPPIX)2.\mathbf{CQ}}$ = 13.17 ± 0.07

A

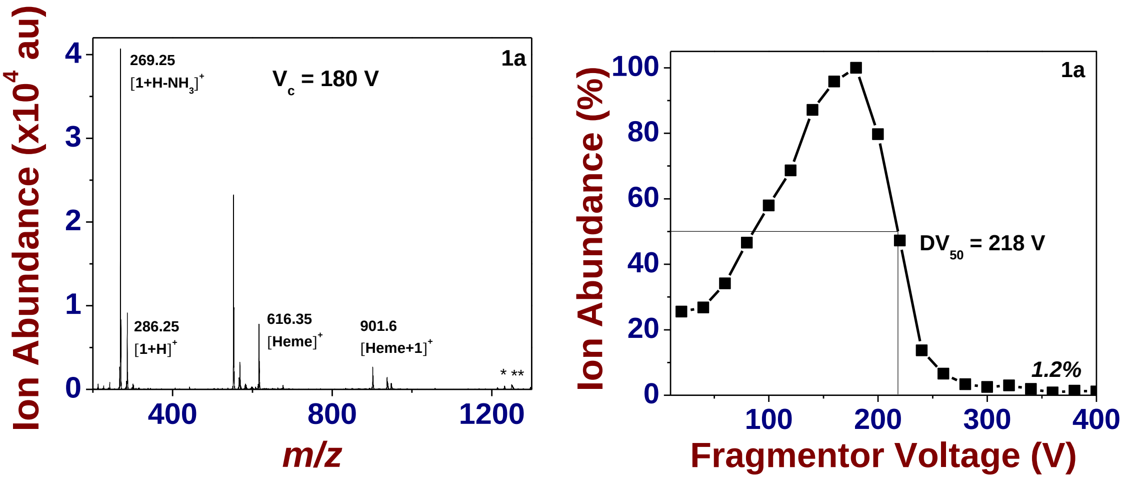
Ion Abundance (x10^4 au)
m/z
269.25
[1+H-NH3]+
V_c = 180 V
1a
286.25
[1+H]+
616.35
[Heme]+
901.6
[Heme+1]+
* **
Ion Abundance (%)
Fragmentor Voltage (V)
DV50 = 218 V
1.2%

B

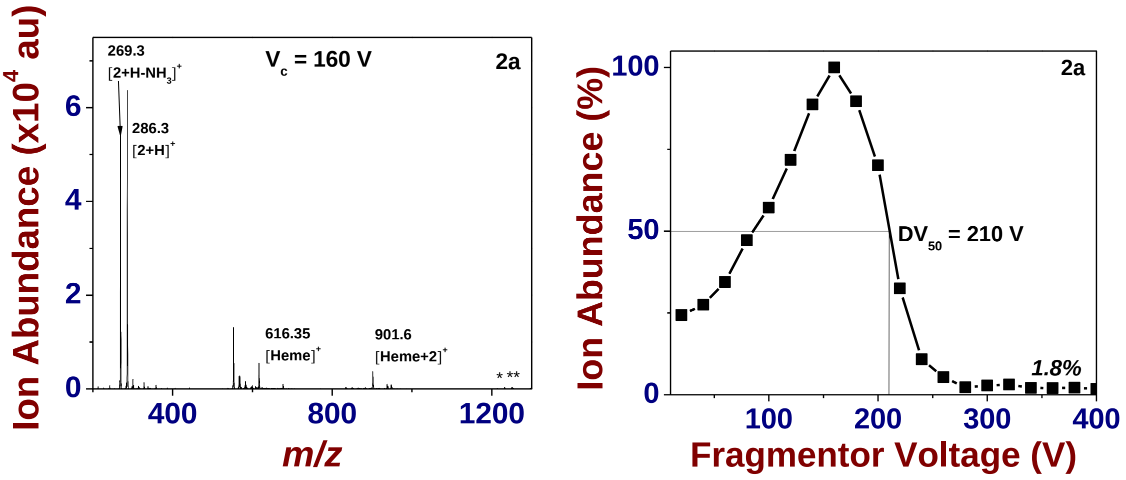
Ion Abundance (x10^4 au)
m/z
269.3
[2+H-NH3]+
V_c = 160 V
2a
286.3
[2+H]+
616.35
[Heme]+
901.6
[Heme+2]+
* **
Ion Abundance (%)
Fragmentor Voltage (V)
DV50 = 210 V
1.8%

C

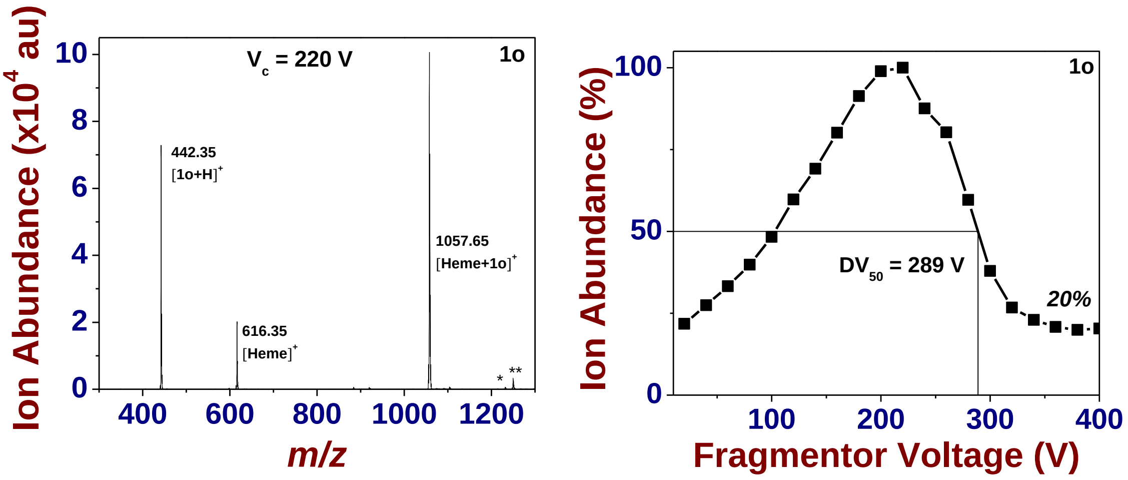
Ion Abundance (x10^4 au)
m/z
V_c = 220 V
1o
442.35
[1o+H]+
616.35
[Heme]+
1057.65
[Heme+1o]+
* **
Ion Abundance (%)
Fragmentor Voltage (V)
DV50 = 289 V
20%

D

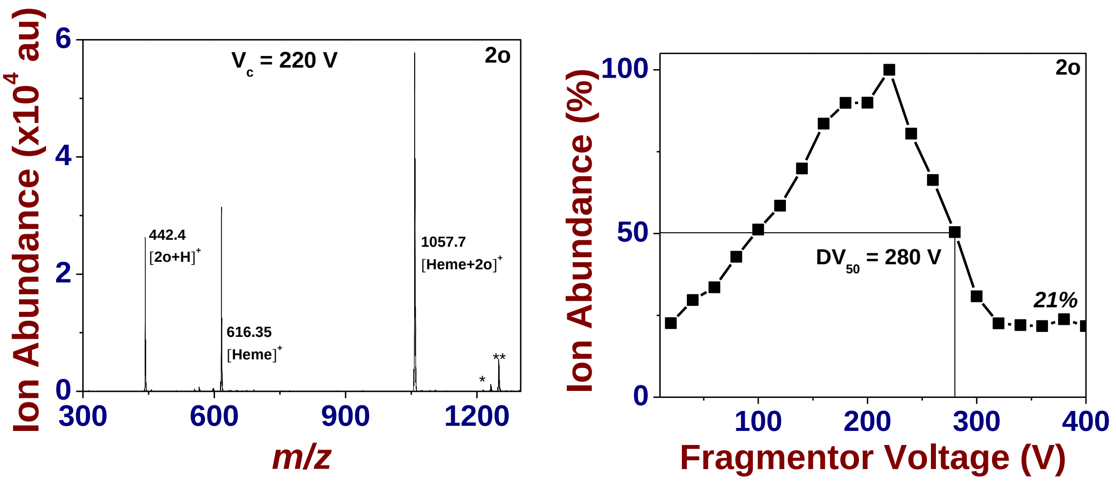

Ion Abundance (x10^4 au)
V_c = 220 V
2o
442.4
[2o+H]+
616.35
[Heme]+
1057.7
[Heme+2o]+
*
**
300
600
900
1200
m/z
Ion Abundance (%)
2o
DV50 = 280 V
21%
100
200
300
400
Fragmentor Voltage (V)

E

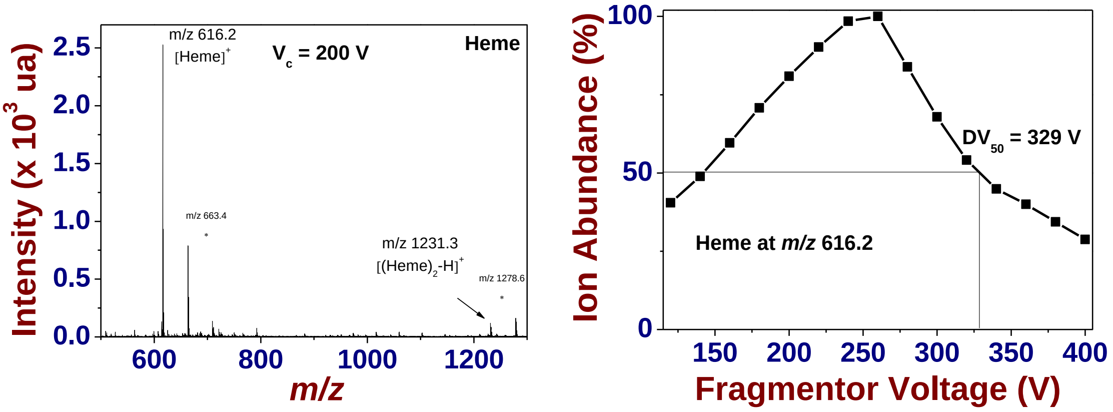

Intensity (x 10^3 ua)
m/z 616.2
[Heme]+
V_c = 200 V
Heme
m/z 663.4
m/z 1231.3
[(Heme)2-H]+
m/z 1278.6
600
800
1000
1200
m/z
Ion Abundance (%)
DV50 = 329 V
Heme at m/z 616.2
150
200
250
300
350
400
Fragmentor Voltage (V)

F

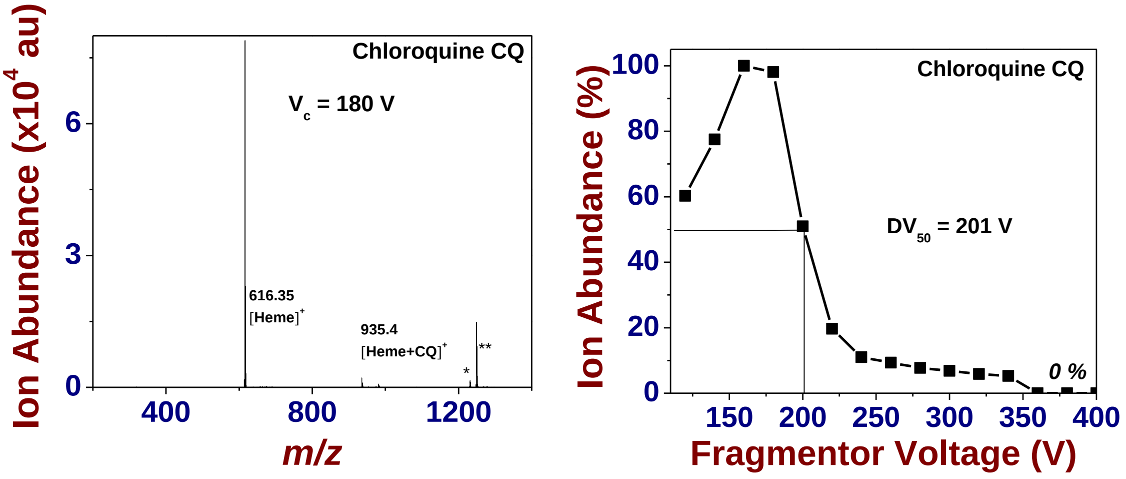

Ion Abundance (x10^4 au)
Chloroquine CQ
V_c = 180 V
616.35
[Heme]+
935.4
[Heme+CQ]+
*
**
400
800
1200
m/z
Ion Abundance (%)
Chloroquine CQ
DV50 = 201 V
0 %
150
200
250
300
350
400
Fragmentor Voltage (V)

G

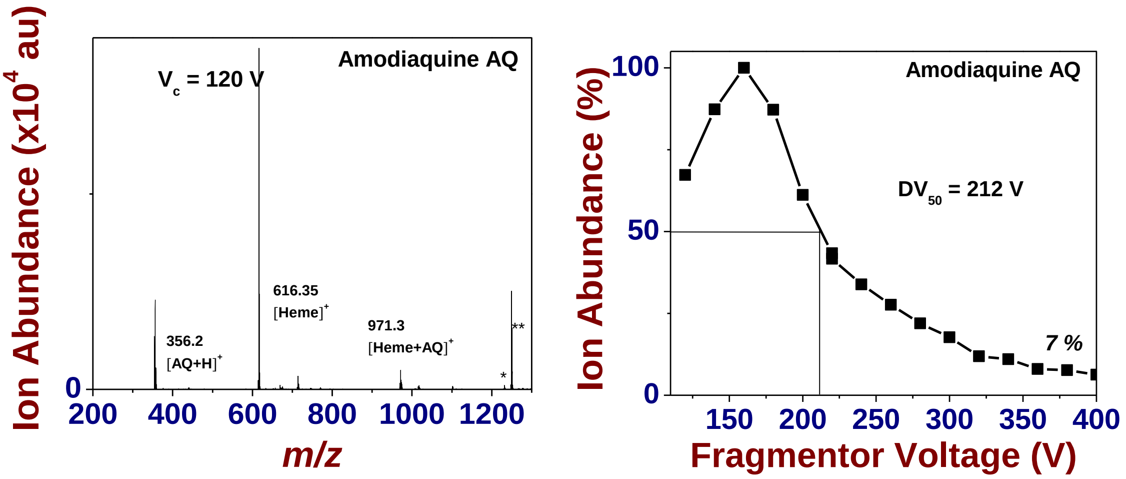

Supplementary Figure 11. (left) ESI mass spectra of a 1:1 mixture of 50 µM haem and 50 µM substrate in H_2O/CH_3CN (5/95) + 0.1% formic acid. (right) Stability responses of the haem substrate adduct obtained from ESI-CID experiments. Positive mode; fragmentor voltage from 20 V to 400 V with 20 V increments. For **1i** and **1k**, 1:1 adducts were detected, but their intensities were too weak to be accurately measured. * = $[(haem)_2\text{-}H]^+$ (m/z = 1231.65); ** = $[(haem)_2\text{+}OH]^+$ (m/z = 1249.65). (A) **1a**; (B) **2a**; (C) **1o**; (D) **2o**; (E) haem; (F) chloroquine **CQ**; (G) amodiaquine **AQ.**

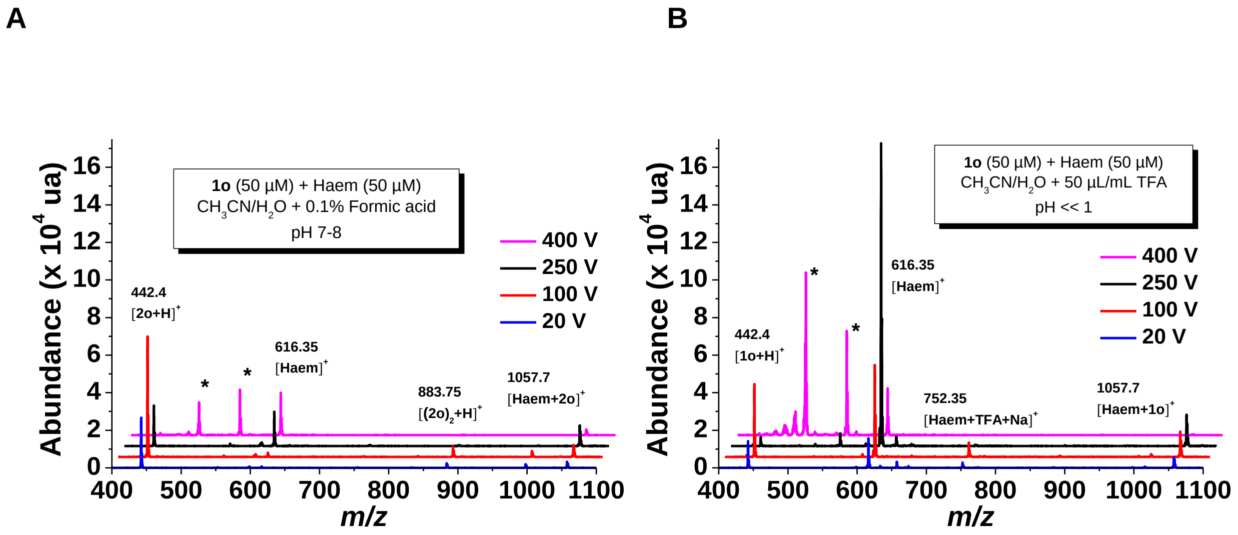

Supplementary Figure 12. ESI mass spectra of a 1:1 mixture of 50 µM haem and 50 µM **1o** in (A) H_2O /CH_3CN (50/50) + 0.1% formic acid and (B) H_2O /CH_3CN (50/50) + 50 µl/ml pure TFA. * = Haem fragmentation products.

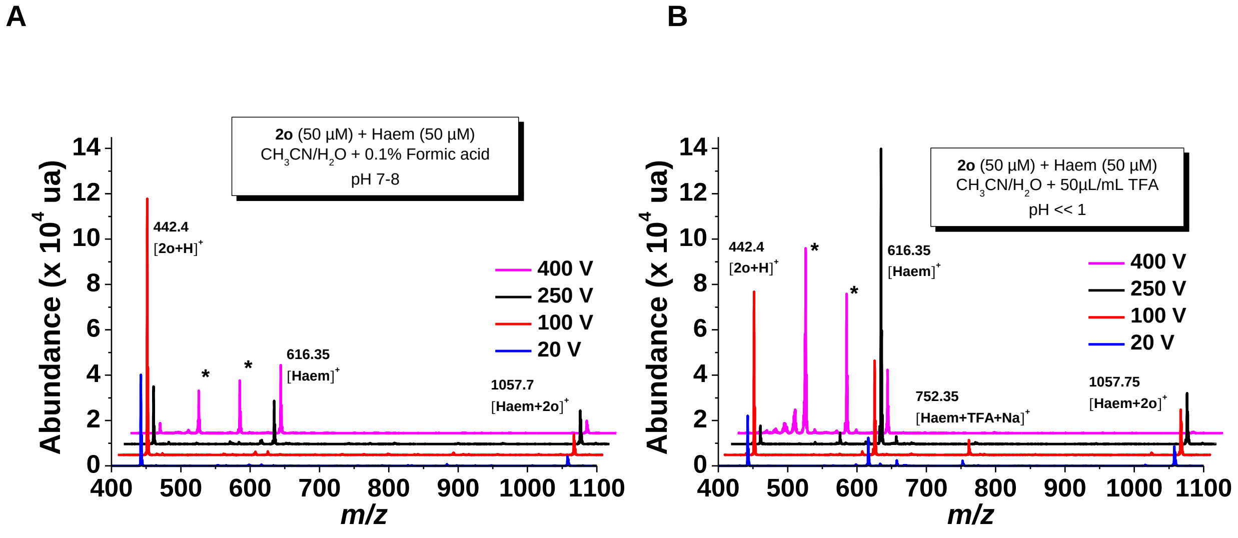

Supplementary Figure 13. ESI mass spectra of a 1:1 mixture of 50 µM haem and 50 µM **2o** in (A) H_2O /CH_3CN (50/50) + 0.1% formic acid and (B) H_2O /CH_3CN (50/50) + 50 µl/ml pure TFA. * = Haem fragmentation products.

A B

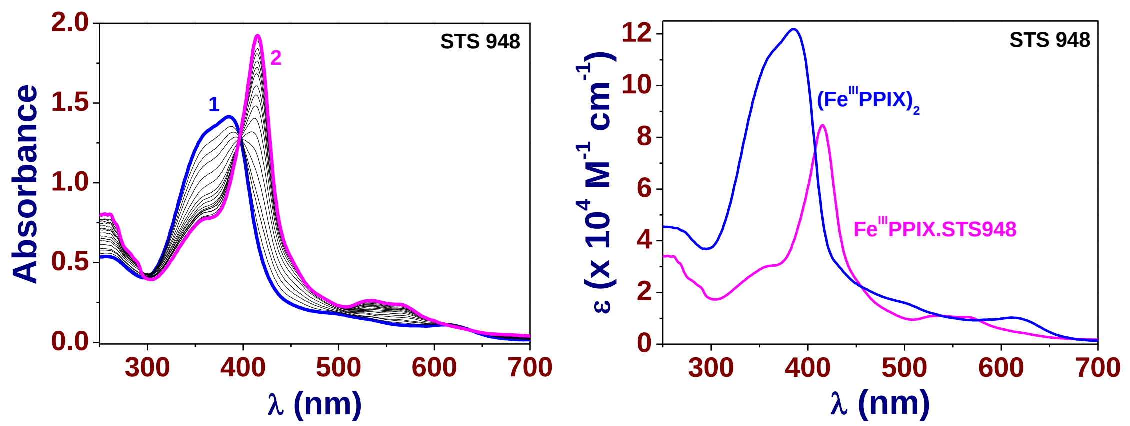

Supplementary Figure 14. (A) Spectrophotometric titration of haem (under its π-π dimeric form, log K_{Dim} = 6.82) via **STS948**, and (B) electronic spectra of haem, of the free substrates, and of its corresponding Fe^{III}PPIX substrate complex with **STS948**. Solvent: 0.2 M aqueous sodium HEPES buffer pH 7.5; T = 25.0 °C; l = 1 cm. For **STS948**: [Fe^{III}PPIX]$_0$ = 2.41 × 10^{-5} M; (1) [**STS948**]$_0$/[Fe^{III}PPIX]$_0$ = 0; (2) [**STS948**]$_0$/[Fe^{III}PPIX]$_0$ = 2.78. log $K_{\mathrm{FeIIIPPIX.\mathbf{STS948}}}$ = 6.09 ± 0.06.

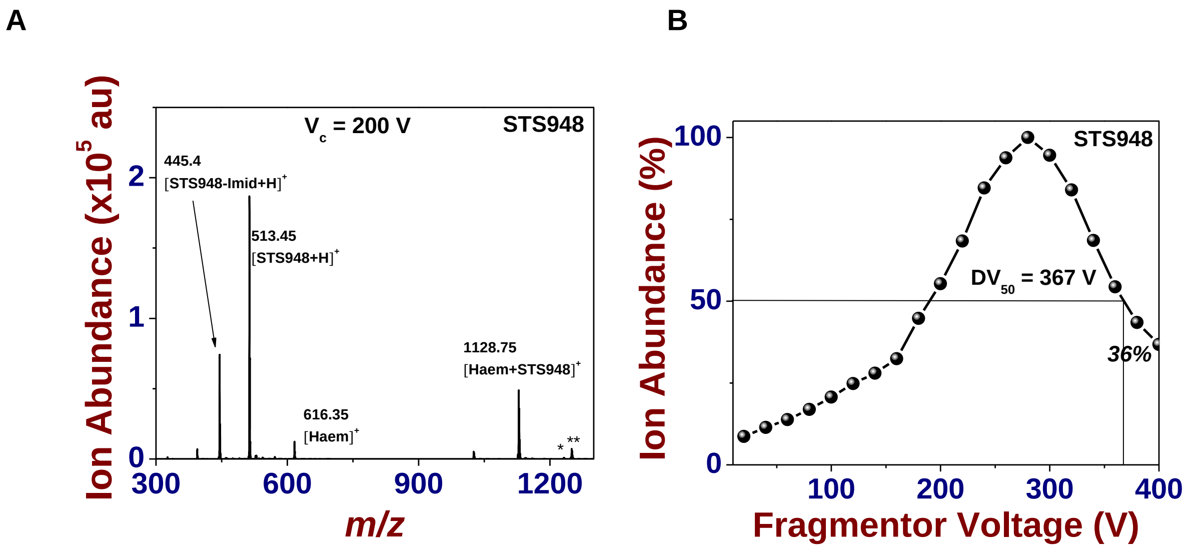

Supplementary Figure 15. (A) ESI mass spectrum of a 1:1 mixture of 50 µM haem and 50 µM **STS948** in H_2O/CH_3CN (50/50) + 0.1% formic acid. (B) Stability responses of the haem.substrate adduct obtained from ESI-MS-CID experiments. Positive mode; fragmentor voltage from 20 V to 400 V with 20 V increments. * = $[(\text{haem})_2\text{-H}]^+$ (*m/z* = 1,231.65); ** = $[(\text{haem})_2\text{+OH}]^+$ (*m/z* = 1,249.65).

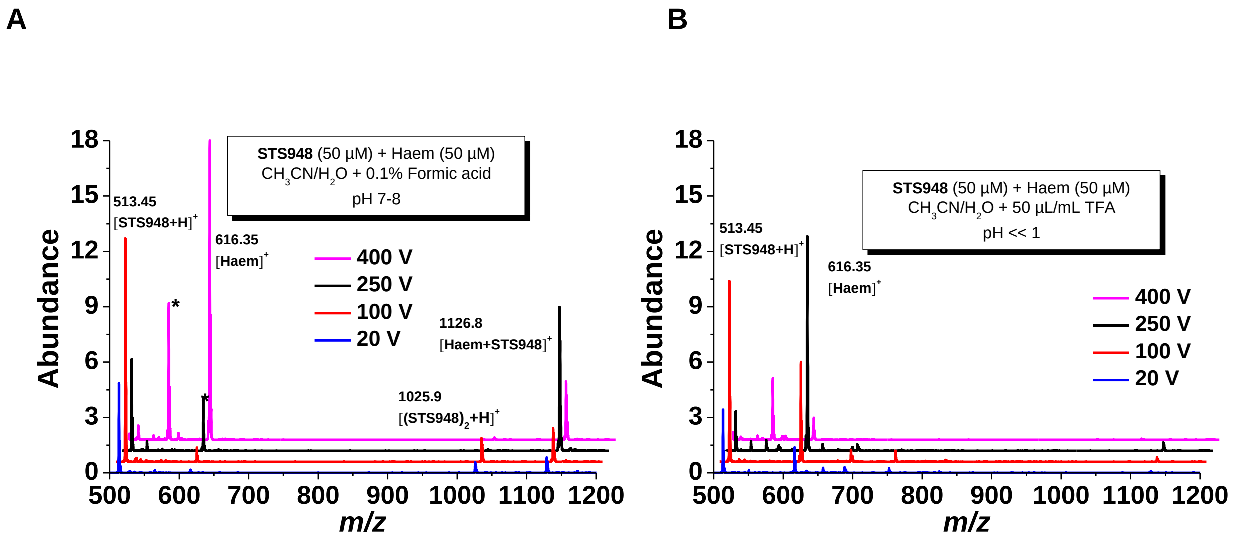

Supplementary Figure 16. ESI mass spectra of a 1:1 mixture of 50 µM haem and 50 µM **STS948** in (A) H_2O /CH_3CN (50/50) + 0.1% formic acid and (B) H_2O /CH_3CN (50/50) + 50 µl/ml pure TFA. * = Haem fragmentation products.

A

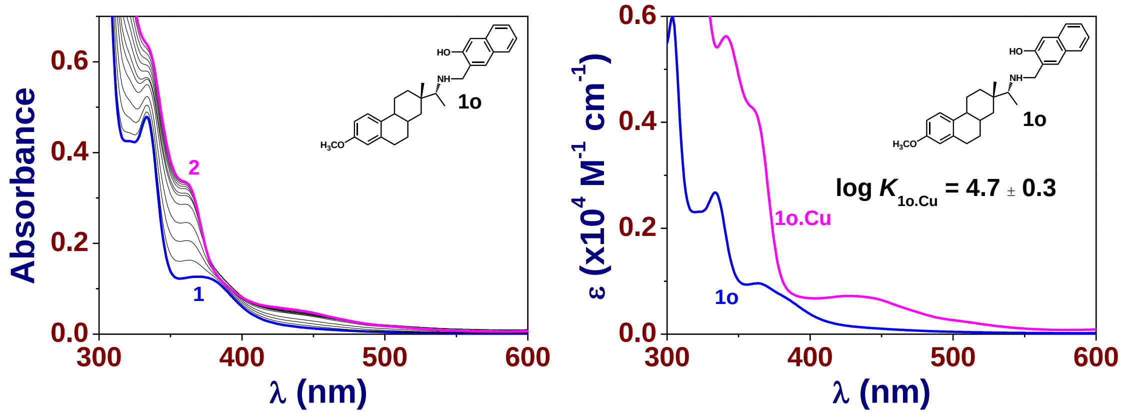

Absorbance
λ (nm)
1
2
1o
ε (x10^4 M^{-1} cm^{-1})
log $K_{1o.Cu}$ = 4.7 ± 0.3
1o.Cu
1o

B

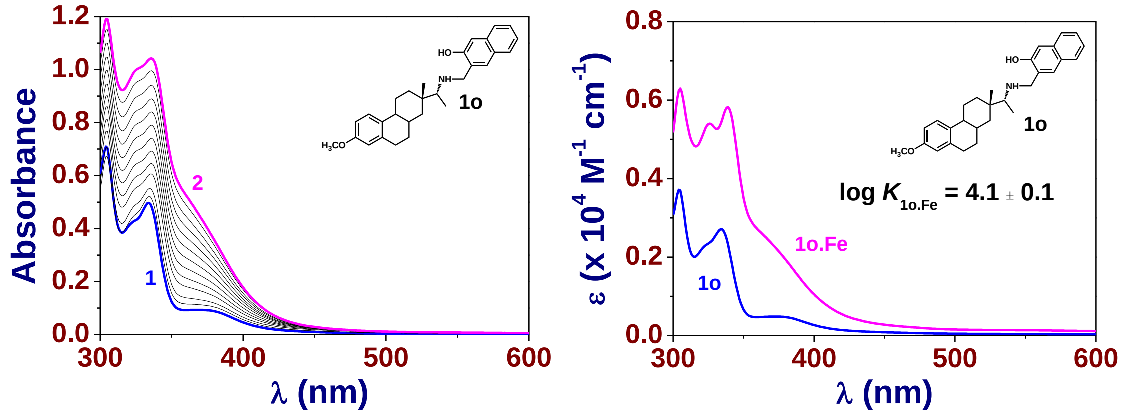

Absorbance
λ (nm)
1
2
1o
ε (x 10^4 M^{-1} cm^{-1})
log $K_{1o.Fe}$ = 4.1 ± 0.1
1o.Fe
1o

C

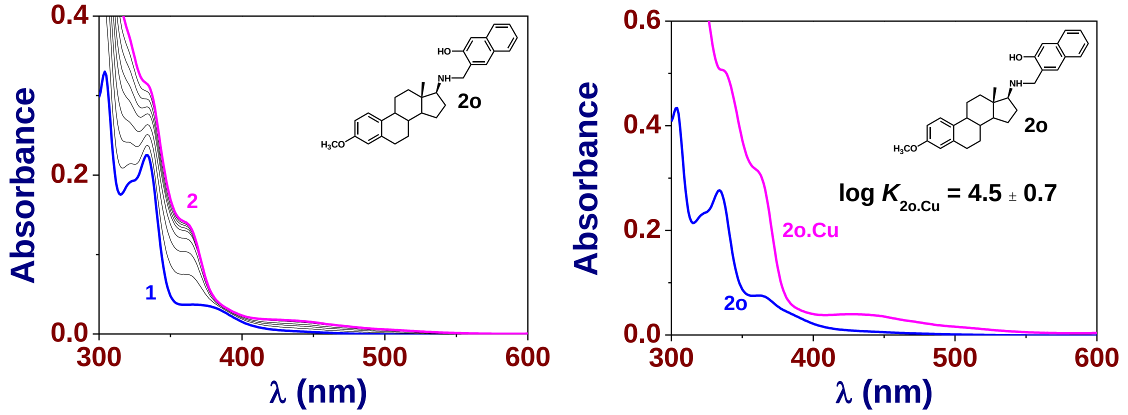

Absorbance
λ (nm)
1
2
2o
Absorbance
log $K_{2o.Cu}$ = 4.5 ± 0.7
2o.Cu
2o

D

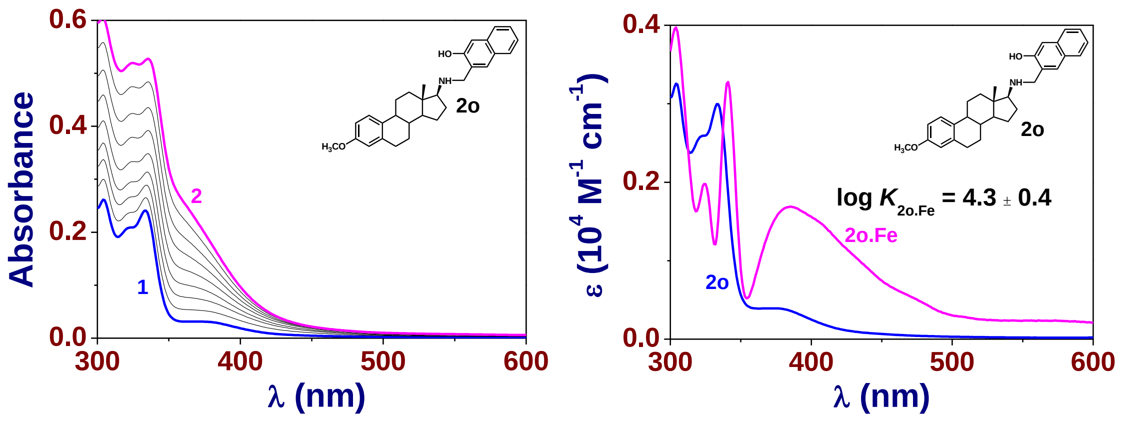

Supplementary Figure 17. (left) Spectrophotometric titration of the substrates **1o** and **2o** by Cu(II) and Fe(III) and (right) electronic spectra of the free substrates and of corresponding metal complexes. Solvent: 0.2 M aqueous sodium HEPES buffer pH 7.5 + DMSO (1:1 *v/v*); T = 25.0 °C; l = 1 cm. (A) (**1**) [**1o**]$_0$ = 1.78 × 10^{-4} M; (**2**) [Cu(II)]$_0$/[**1o**]$_0$ = 0.67; (B) (**1**) [**1o**]$_0$ = 1.78 × 10^{-4} M; (**2**) [Fe(III)]$_0$/[**1o**]$_0$ = 1.0; (C) (**1**) [**2o**]$_0$ = 8.1 × 10^{-5} M; (**2**) [Cu(II)]$_0$/[**2o**]$_0$ = 1.47; (D) (**1**) [**2o**]$_0$ = 1.78 × 10^{-4} M; (**2**) [Fe(III)]$_0$/[**2o**]$_0$ = 2.2.

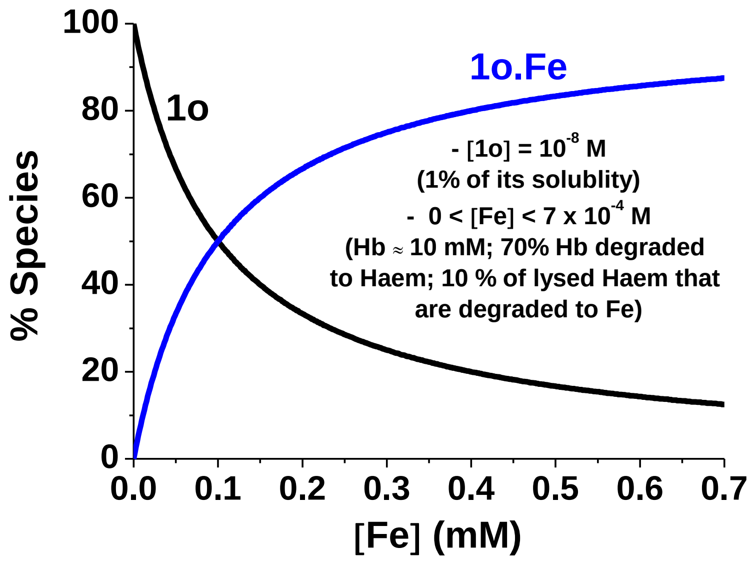

Supplementary Figure 18. Species diagram distribution showing the formation of **1o**:Fe complexes within parasitized RBCs and assuming relevant Hb catabolites and **1o** parameters.

Supplementary Tables

Compound	IC_{50} [nM]	Compound	IC_{50} [nM]
**1a**	>1,000	**4c**	63
**1b**	>2,500	**5c**	160
**1c**	**7.4**	**5s**	99
**1e**	434	**6c**	676
**1g**	116	**6w**	1,620
**1i**	872	**7c**	120
**1k**	1,010	**7s**	55
**1o**	**4.1**	**8b**	>2,500
**1s**	11	**8c**	224
**2a**	>1,000	**9c**	999
**2b**	>2,500	**10c**	84
**2c**	**73**	**11c**	141
**2e**	741	**12c**	4,360
**2m**	183	**13c**	465
**2n**	5,540	**14c**	208
**2o**	**6.6**	**15c**	>6,000
**2p**	>5,000	**16c**	>6,000
**2q**	713	**17c**	2,000
**2s**	62	**18c**	4,000
**2u**	114	**19c**	>15,000
**3a**	>1,000	**20b**	>15,000
**3c**	42	**20c**	5,600
**4a**	>1,000	**20n**	>13,000
		**20o**	>13,000

Supplementary Table 1. IC_{50} values of the steroid derivatives determined *in vitro* on the *Plasmodium falciparum* strain 3D7.

Substance	Dosage mg/kg			Route	Parasitized RBC over 100					Average	% of control	% activity	Mouse survival in days					Average	Parasitized RBC after 30 d §		
					M1	M2	M3	M4	M5				M1	M2	M3	M4	M5		M1	M2	M3
**1c**	3x	10	T/A	**p.o.**	28	26	53			36	53	**47**	8	11	7			9			
**1c**	3x	30	T/A	**p.o.**	0.53	0.31	0.48			0.44	0.65	**99.4**	13	12	14			13			
**1c**	3x	100	T/A	**p.o.**	0.23	0.14	0.23			0.20	0.30	**99.7**	15	17	14			15			
**1c**	3x	10	T/A	**s.c.**	57	68	42			56	83	**<40**	7	6	8			7			
**1c**	3x	30	T/A	**s.c.**	21	24	32			26	38	**62**	14	14	14			14			
Control (d4)					48	74	72	69	76	68			4	4	4	4	4	4			
**1o**	4x	100	T/E	**i.p.**	0.10	0.10	0.10			0.10	0.17	**99.8**	30	30	30			>30	0.0	0.0	0.0
Control (d4)					63	49	55	69	57	59			4	4	4	4	4	4			
**1o**	4x	100	T/E	**p.o.**	0.00	0.30	0.10			0.13	0.22	**99.8**	30	30	20			27	0.0	0.0	
Control (d3)					62	56	60	55	66	60			4	4	4	4	4	4			
**1o**	1x	100	T/E	**p.o.**	0.30	0.60	0.50			0.47	1.5	**98**	14	11	14			13			
Control (d3)					31.0	29.8	31.6	27.3	32.9	30.52			3	3	3	3	3	3			

Supplementary Table 2. ***In vivo*** **activity of compounds 1c and 1o in the** ***Plasmodium berghei*** **mouse model (Peter's test).** 10-100 mg/kg bodyweight of the respective compound was given via oral administration (p.o.), subcutaneously (s.c.), or intraperitoneally (i.p.) on 3 or 4 consecutive days.4 x 100 mg/kg **1o** i.p. reduced parasitaemia by 99.8% and cured all mice.

d, day; M, mouse; § parasitized RBC after 30 d (microscope).

Compound	Concen-tration	Solvent	*Bacillus subtilis JMRC:STI:10880* B1	*Staphylo-coccus aureus JMRC:STI:10760* B3	*Escherichia coli JMRC:ST:33699* B4	*Pseudo-monas aeruginosa JMRC:ST:33772* B7	*Pseudo-monas aeruginosa JMRC:ST:337721* B9	*MRSA Staph. aureus JMRC:ST:33793* R9	*VRSA Enteroc. faecalis* 1528 R10	*Mycobac. vaccae* 10670 M4	*Sporobolo. salmoni-color* 549 H4	*Candida albicans* C. alb. H8	*Penicillium notatum* JP36 P1
**1o**	1,000 µg/ml	DMSO	0	0	15P	0	0	0	0	18p	22p	10/14p	0
**2o**	1,000 µg/ml	DMSO	0	0	16P	0	0	0	0	17p	24p	10/13P	0
**Cipro-floxacin**	5 µg/ml	distilled water	31	19	23/33p	27	28/37p	0	16F	21(p)			
**Ampho-tericin B**	10 µg/ml	DMSO/ MeOH									18p	21	19p
**Solvent**		DMSO	12P	12P	13P	13P	16P	12P	12P	13P	20p	0	13p

Supplementary Table 3. Antibacterial profile of the steroid compounds 1o and 2o: p = colonies in the inhibition zone; P = many colonies in the inhibition zone; A = indication of inhibition; F = facilitation.

Compound	Start concen-tration	*Alternaria alternata JMRC:SF:09317*	*Arthroderma benhamiae JMRC:ST:35888*	*Aspergillus fumigatus JMRC:Afum:0007 3*	*Aspergillus terreus JMRC:SF:0630 7*	*Candida albicans JMRC:ST:3586 4*	*Lichtheimia corymbifera JMRC:SF:09682*	*Penicillium chrysogenum JMRC:SF:1013 7*	*Rhizopus arrhizus* FSU 5857
		48 h	72 h	48 h	48 h	48 h	48 h	48 h	48 h
**1o**	1 mg/ml	500/250	500/1mg	250/250	250/125	1 mg/500	500/62.5	1 mg/125	500/500
**2o**	1 mg/ml	125/15,6	31.2/2	250/125	250/4	500/250	62.5/7.8	250/125	500/500
**Amphotericin B**	100 µg/ml	0.78/0.1	3.12/0.2	3.12/0.4	50/0.2	0.4/0.2	<0.05/<0.05	100/1.56	25/1.56
**Solvent**		100/0	50/12.5	100/25	100/12.5	100/100	100/100	100/100	50/50

Supplementary Table 4: Antifungal profile of the steroid compounds 1o and 2o: Dilution of test germs: 2 x 10^4; solvent: DMSO/MeOH; 1st value = microscopic visualization-based observation; 2nd value = optical density-based observation.

**Parameter**	**C57BL/6 mice (n=3/time point)**		
**Route**	IV	IP	PO
**Dose (mg/kg)**	1	100	100
**T_{max} (h)**		3.0	8.25
**C_{max} (ng/ml)**		850	43
**Vdss (l/kg)**	6.40		
**$AUC_{0\text{-}last}$ (h*ng/ml)**	567	12,955	622
**Cl (ml/min/kg)**	29.6		
**$t_{1/2}$ (h)**	>8	>8	>8
**%F**		24	<5

Supplementary Table 5. Mouse pharmacokinetics of compound 1o.

**Aqueous Solubility (Pion's Buffer)**<br>pH 5.0/6.2/7.4<br>µg/ml	0.26/0.38/0.30
**Hepatic Microsome Stability**<br>% remaining after 1 h<br>Human<br>Mouse	 <br>38.47<br>38.51
**Plasma Stability**<br>% remaining after 3 h<br>Human<br>Mouse	 <br>62.46<br>58.91
**Plasma Protein Binding**<br>% bound<br>Human 1 µM/10 µM<br>Mouse 1 µM/10 µM	 <br>99.13/99.06<br>99.06/98.93
**PAMPA**<br>Pe (x10^{-6} cm/s)<br>Donor pH 5.0/6.2/7.4<br>Acceptor pH 7.4	(0/0/0)*

*Although very poorly permeable at all 3 pH levels tested, the compound was well embedded in the filter membrane.

Supplementary Table 6. Pharmacokinetic properties of compound 1o.

**Compound**	Molar mass (g/mol)	Adduct [Haem+S] m/z	DV_{50} (V)	Bottom plateau (%)	Dissociation Constant K_D (µM)
**1o**	441.62	1,057.65	290	20	3.3
**2o**	441.62	1,057.7	280	21.7	8.3
**1a**	285.43	901.6	218	1.2	6.6
**2a**	285.43	901.6	210	1.8	n/a
**1h**	405.58	n/a	n/a	n/a	6.6
**2h**	405.58	n/a	n/a	n/a	n/a
**CQ**	319.87	935.4	201	0	1.3
**AQ**	355.86	971.3	212	7	n/a

Supplementary Table 7. ESI-MS-CID data (m/z, calculated DV50 = dissociation voltage at 50% and % bottom plateau) of the [haematin-drug]+ adducts and dissociation constants (K_D, µM) of the haematin-drug complexes measured with absorption spectrophotometric titrations. Conditions: 50 µM haem and 50 µM substrate in H_2O/CH_3CN (5/95) + 0.1% formic acid. Stability responses of the haem substrate adduct were obtained via ESI-CID experiments. Positive mode; fragmentor voltage from 20 V to 400 V with 20 V increments.

Supplementary Notes

Supplementary Note 1 - Synthesis: Experimental data for procedures and characterization of compounds

General

All reagents, unless otherwise noted, were received from commercial providers (Sigma-Aldrich®, VWR®, and Acros Organics®) and were used in the offered quality. Reactions were preferably performed under argon or nitrogen in the absence of moisture. Products were dried in a vacuum desiccator at ≈10 Torr for several days over phosphorus pentoxide at room temperature.

TLC: Reactions were monitored using Merck aluminium-backed silica gel 60 plates F_{254} or Fluka aluminium-coated plates (Al_2O_3), UV detection at 254 nm, or detection by spraying with a mixture of 80 ml concentrated sulfuric acid, 20 ml EtOH, 200 mg vanillin (4-hydroxy-3-methoxybenzaldehyde), and subsequent visualization by heating at 170 °C; eluent: AcOEt/n-hexane (1:2, v/v) unless not stated otherwise. - NMR: Spectra were recorded at 298 K on a Bruker AC 250 (^{1}H: 250 MHz, ^{13}C: 63 MHz) and AC 400 (^{1}H: 400 MHz, ^{13}C: 100 MHz). As an internal standard, the chemical shift of the residual protons of the deuterated solvent was used. Chemical shifts δ are given in ppm. - MS: Mass spectra were recorded on the devices FISONS Trio-200, FINNIGAN MAT SSQ 710, and SHIMADZU Biotech Axima LNR. - mp: Melting points were determined using a micro hot stage according to BOËTIUS. - Analysis: Elemental analysis was performed using a Foss-Heraeus CHN-O-RAPID or micro-analysis system according to Knobloch, M is given in g/mol. - IR: Shimadzu IRAffinity-1 and Bio-RAD FTS 175 UMA-500 spectrometers using KBr-pellets, if not noted otherwise; alternatively Nujol or an ATR moiety (Specac MKII Golden Bridge) were applied; v is given in cm^{-1}. - $[\alpha]_D$: Specific rotational values were estimated with a Polamat A (Carl Zeiss Jena) or PROPOL DIGITAL automatic polarimeter (Dr. Kernchen) at room temperature in chloroform (c =1)

unless otherwise not noted; c is given in g 100^{-1} ml^{-1}, $[\alpha]_D$ in °. – Abbreviations - spectral data: s - strong, m - middle, w - weak, v - very (e.g. vs - very strong), overl. - overlaid, sh - shoulder, aliph. - aliphatic, arom. - aromatic.

Synthesis

All steroid arylmethylamines described herein were prepared by condensing amino steroids (series **1**-**14**, Figure 1a in main text**)** with aryl aldehydes followed by subsequent reduction of the obtained *Schiff* bases. Typical overall yields are in the 60-90% range. The non-steroidal analogs (series **15**-**21**) were obtained in the same manner. Corresponding substitution patterns **a**-**w** are compiled in Figure 1b of the main text.

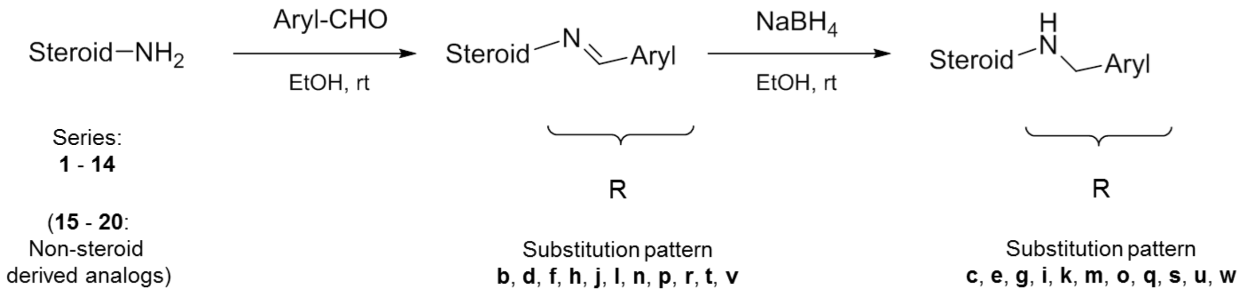

1. Amino steroids 1a - 14a

Estratriene derivatives were prepared from commercially available estrone methylether (series **1** - **8**, **10**, and **11**) or estrone (series **9**), following published protocols as outlined below:

17α-amino-3-methoxy-estra-1,3,5(10)-triene **1a** [1]

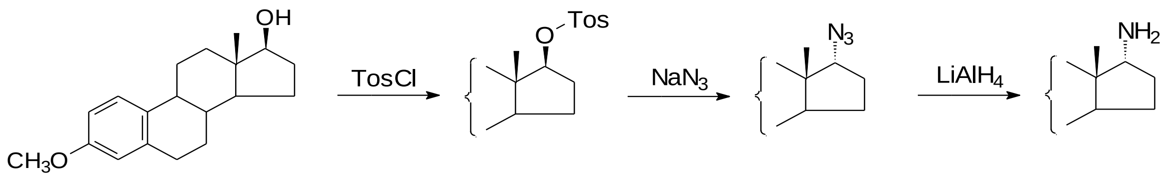

17β-amino-3-methoxy-estra-1,3,5(10)-triene **2a** [1]

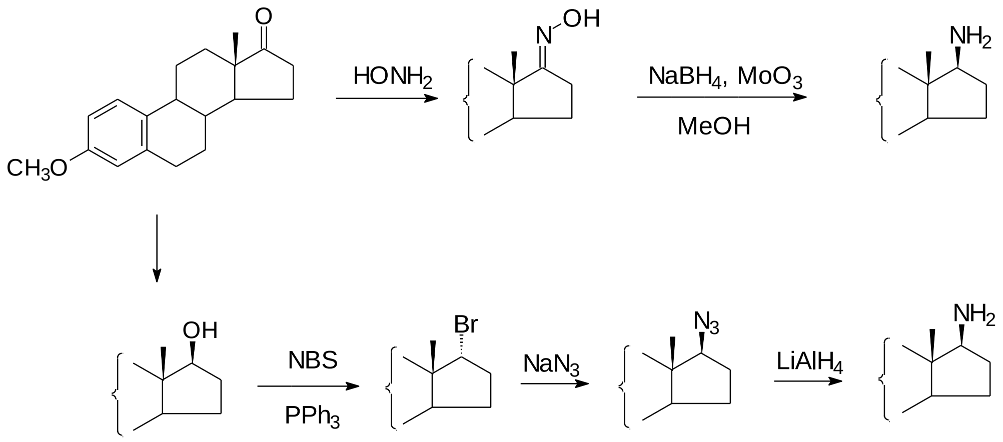

17α-amino-3-methoxy-estra-1,3,5(10)-triene **3a** [2] and 17β-amino-3-methoxy-estra-1,3,5(10)-triene **4a** [3]

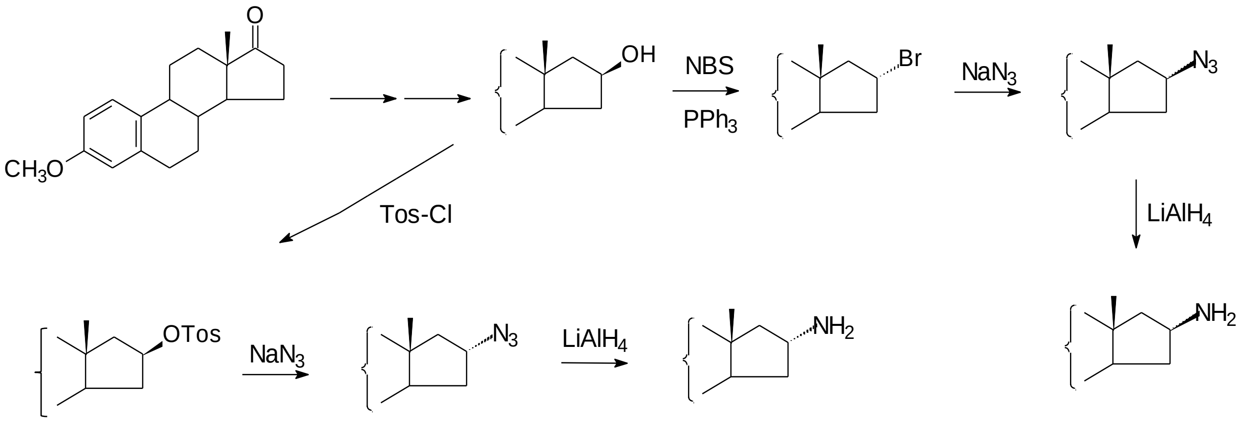

16α-amino-17-hydroxy-3-methoxy-estra-1,3,5(10)-trienes **5a** and **7a** [4]

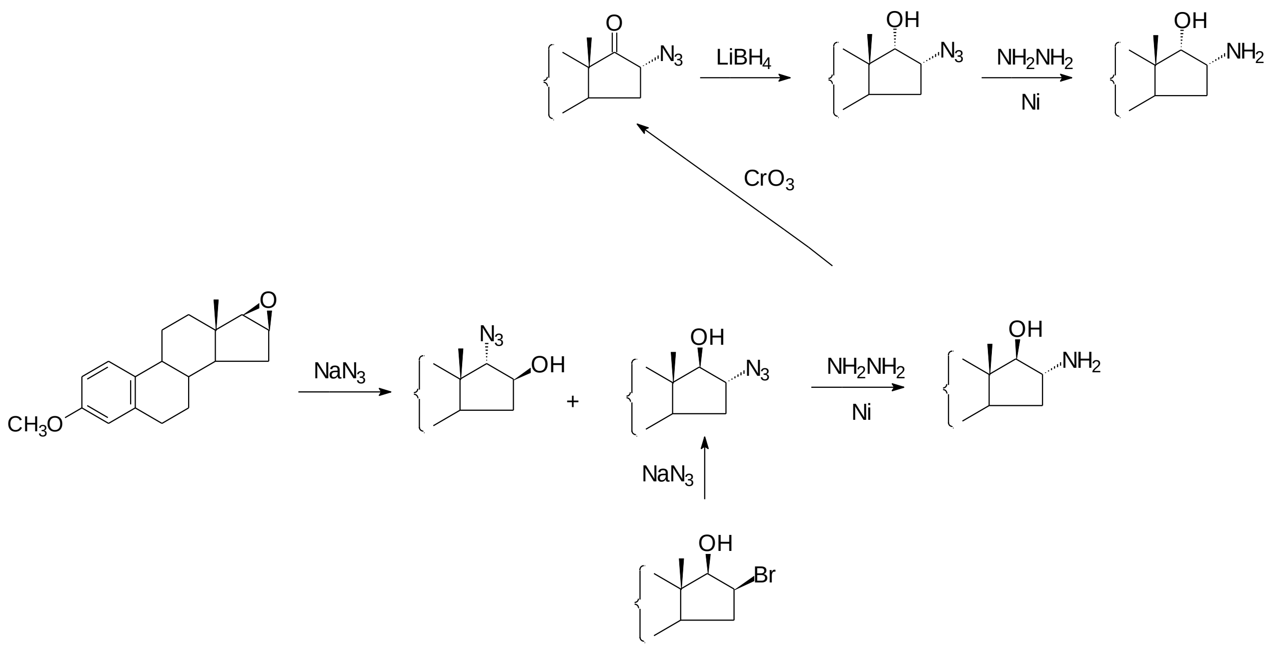

16β-amino-17-hydroxy-3-methoxy-estra-1,3,5(10)-trienes **6a** and **8a** [4]

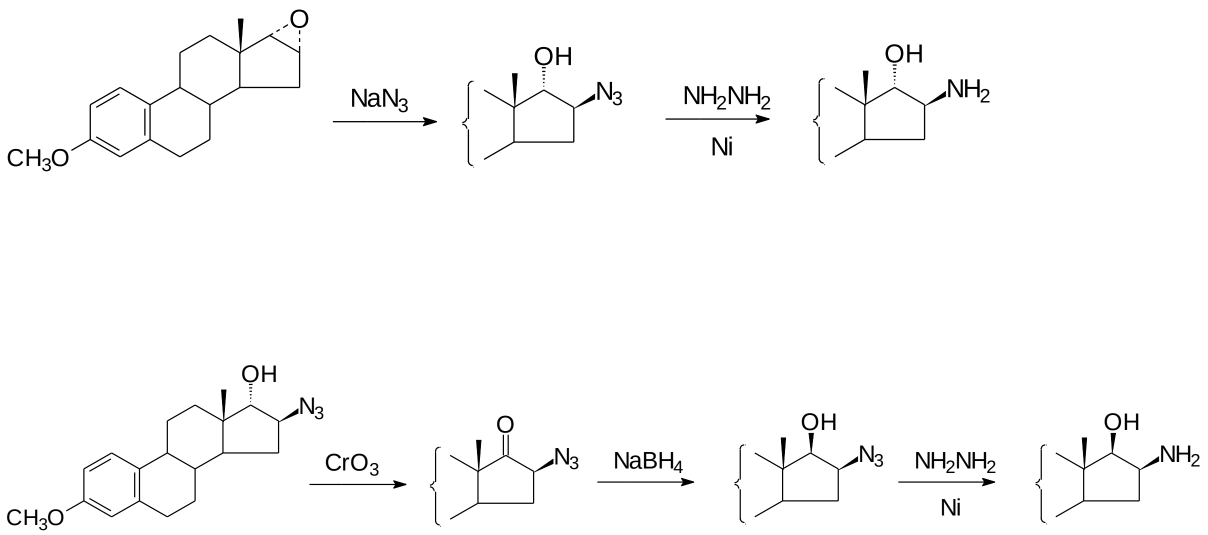

3-hydroxy-estratriene series - amino alcohol **9a** [5] and precursors [6]

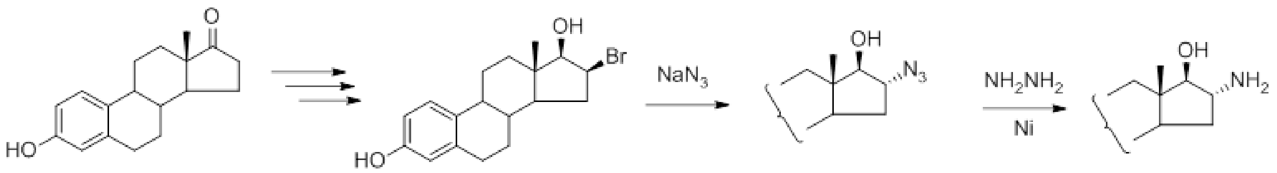

13-epi-estratriene series - amino alcohols **10a** [7] and **11a** [7] and precursors [8]

10a:

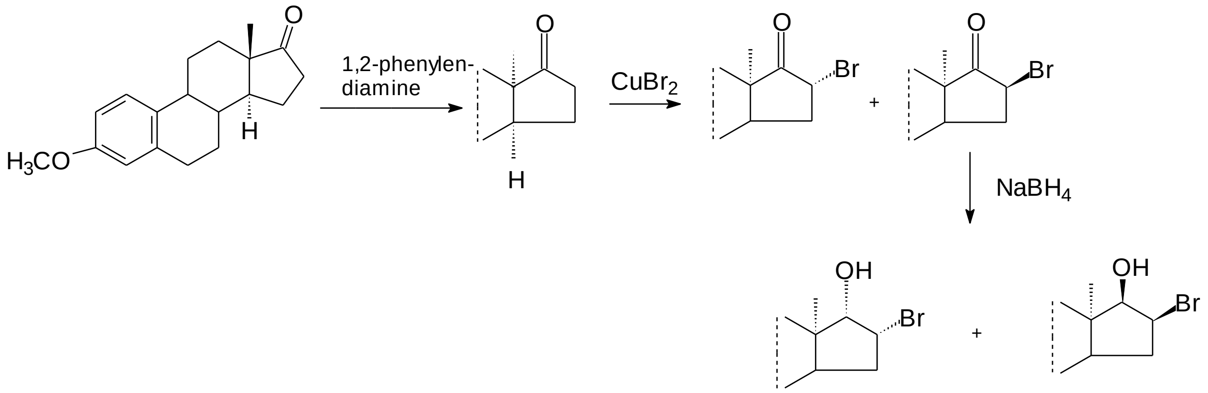

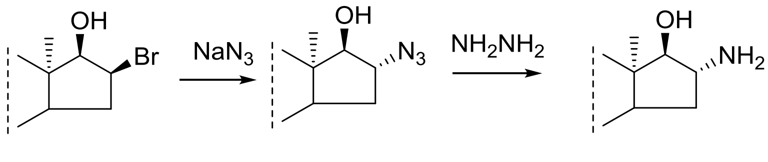

11a:

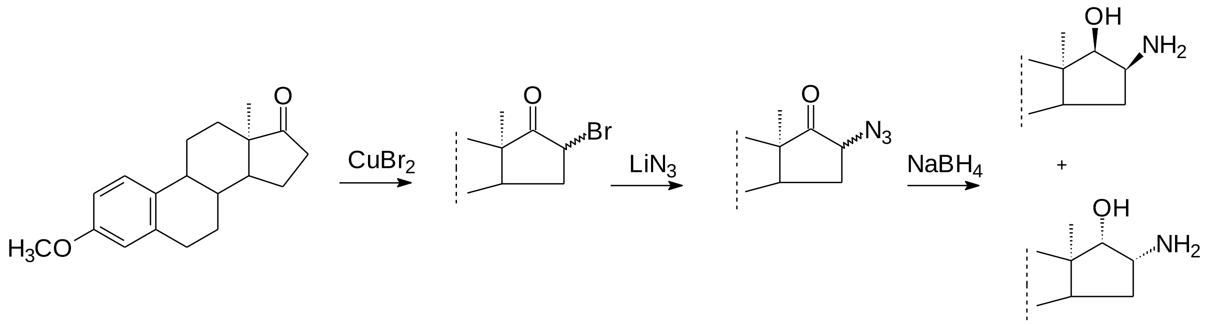

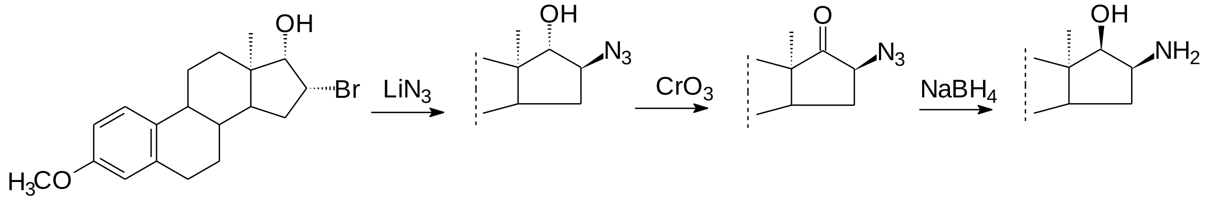

cholane series - amino alcohols **12a**, **13a**, and **14a**

12a [9]

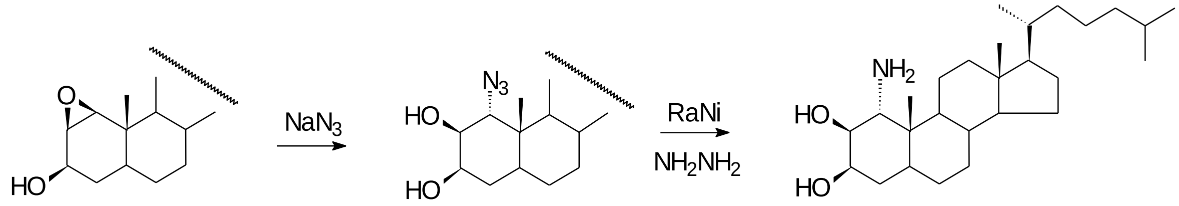

13a and **14a** [1]

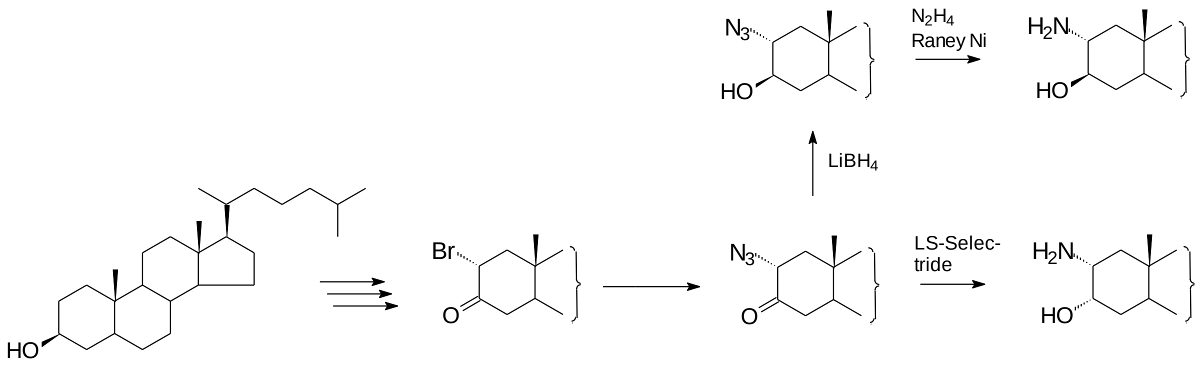

Synthesis of 3-hydroxynaphth-2-carbaldehyde (substitution-patterns **n** and **o**) from 2-methoxy naphthalene is described in ref. 11.

2. Steroid *Schiff* bases and corresponding arylmethylamino steroids

General procedure 1 - Schiff *bases via condensation of amino steroids* ***1a-14a*** *with aryl aldehydes*

1.0 mmol of the amino compound was dissolved in 15 ml of absolute EtOH and mixed with 1.1 mmol of the corresponding aldehyde. After stirring at room temperature for 2-4 h (TLC control), the yellow solution was concentrated to 50% of its initial volume. The resulting precipitate was collected via filtration, washed with MeOH/water, dried, and recrystallized. Alternatively, the solvent was removed completely.

General procedure 2 - preparation of arylmethylamino steroids via reduction of Schiff *bases with* $NaBH_4$

$NaBH_4$ (2.0 mmol, 76 mg) was added to a stirred solution or suspension of 1.0 mmol of *Schiff* base in 10 ml of abs. EtOH at room temperature. Typically, after 5-20 min the homogeneous solution turns colorless. After an additional 15 min, ice, water, and a few drops of acetic acid

were added slowly to the stirred solution. The obtained precipitate was filtered off, washed with water, and dried. The residue was crystallized as described below from MeOH, EtOH, EtOH/water, or AcOEt/hexane. Oily products were poured into 30 ml of CH_2Cl_2, extracted with water (3 times), and dried over Na_2SO_4.

General procedure 3 – one-step preparation of arylmethylamino steroids The synthesis could also be carried out advantageously as a one-pot procedure: 1.0 mmol of amino compound was dissolved in 15 ml of a 1:1 (v/v) mixture of absolute EtOH and THF, mixed with 1.1 mmol of aldehyde, and stirred at room temperature for 2-4 h (TLC control). To the yellow solution of *Schiff* base (or a mixture of corresponding tautomers in case of *cis*-amino alcohols), 113.5 mg (3.0 mmol) of $NaBH_4$ was added and stirred at room temperature until discoloration occurred (about 10-60 min). After slowly adding water/ice and a few drops of acetic acid, the precipitate was filtered off, washed with water, dried, and crystallized from MeOH, EtOH, EtOH/water, or AcOEt/hexane. Oily products were poured into 30 ml of CH_2Cl_2, extracted with water (3 times), and dried over Na_2SO_4. After evaporation of the solvent, the residue was purified via crystallization as described above or via column chromatography using silica gel and AcOEt/*n*-hexane as eluent. If necessary, less lipophilic and water-soluble compounds (series **15**-**20**) were isolated as hydroperchlorates in the following manner: The decolorized reaction mixture was added with ice/water and some drops of acetic acid as described above, concentrated to about 10-40% of its initial volume, acidified with perchloric acid (pH ≈ 2), and allowed to stand at 4 °C overnight. The obtained precipitate was collected via filtration, washed with water, dried, and recrystallized as described below. Precipitation of the reaction product can be promoted by adding a concentrated solution of ammonium perchlorate.

Steroid-derived Schiff *bases and corresponding antimalarials (series **1**-**14**)*

17α-[*(E)*-2-hydroxyphenylcarbaldimino)-3-methoxy-estra-1,3,5(10)-triene **1b**

Procedure 1. $C_{26}H_{31}NO_2$ (389.55), mp: 161-162.5 °C (from MeOH), yield 83%, yellow plates, TLC (AcOEt:hexane, 1:4 v/v): R_f = 0.64.

Analysis (calcd., found): C (80.17, 80.27), H (8.02, 7.70), N (3.60, 3.57). $[\alpha]_D$ = -116.3 (c = 8.432).

^{1}H NMR (250 MHz, $CDCl_3$): δ 0.864 (s, 3H, 18-H_3), 2.855 (m, broad, 2H, 6-H_2), 3.357 (d, J = 6.6 Hz, 1H, 17β-H), 3.760 (s, 3H, 3-MeO), 6.662 (m, 2H, 2-H und 4-H), 6.889 (m, 2H, 3- and 5-phenyl-H), 7.254 (m, 3H, 1-H and 4-H and 6-phenyl-H), 8.246 (s, 1H, N=CH), 13.754 (s, 1H, 2-phenyl-OH).

^{13}C NMR (63 MHz, $CDCl_3$, DEPT 135): δ 18.5 (18-CH_3), 25.1 (15-C), 26.4 ((11-C), 28.1 (7-C), 30.0 (6-C), 31.7 (12-C), 33.4 (16-C), 55.2 (CH_3O), 78.7 (17-C), 111.5 (2-C), 113.8 (4-C), 126.4 (1-C), 132.0 (10-C), 138.0 (5-C), 157.4 (3-C), 162.0 (CH=N); phenyl-C: 116.9, 118.5, 118.8, 131.1, 132.8, 161.0.

IR (ATR): 1630 (s, C=N); 1611 (m), 1581 (m), and 1500 (m, arom. C=C).

17α-(2-Hydroxyphenyl-methylamino)-3-methoxy-estra-1,3,5(10)-triene **1c**

Procedure 2. $C_{26}H_{33}NO_2$ (391.55), mp: 145.5-147.5 °C (from MeOH), yield 95%, white crystals, TLC: R_f = 0.71, $[\alpha]_D$ = +7.1 (c = 10.508).

ESI-MS: $[MH]^+$ = 392.3 (100%); HRMS (m/z): $[M^+]$ calcd. for $C_{26}H_{34}NO_2$, 392.25895; found, 392.25835.

^{1}H NMR (250 MHz, $CDCl_3$)**: δ** 0.914 (s, 3H, 18-H_3), 1.458-2.364 (m, overl.,13H, aliph. H), 2.788 (d, ^{3}J = 6.6 Hz, 1H, 17β-H), 2.868 (m, broad, 2H, 6-H_2), 3.789 (s, 3H, 3-MeO), 3.826 (d, ^{2}J = 13.8 Hz, 1H, NH-C$\underline{H}_2$-phenyl), 4.062 (d, ^{2}J = 13.8 Hz, 1H, NH-C$\underline{H}_2$-Phenyl), 6.645 (s, 1H, 4-H), 6.726 (d, ^{3}J = 8.4 Hz, 1H, 2-H), 6.760-6.866 (m, overl., 2H, 2x Ar-H), 7.014 (d, ^{3}J = 8.3 Hz, 1H, 1-H), 7.158-7.205 (m, overl. 2H, Ar-H).

^{13}C NMR (73 MHz, $CDCl_3$): δ 19.08, 24.82, 26.31, 28.08, 29.05, 29.89, 32.83, 39.24, 43.56, 44.95, 49.59, 51.06, 55.20, 66.70, 111.49, 113.80, 116.42, 118.94, 122.96, 126.36, 128.13, 128.70, 132.51, 137.92, 157.47, 158.34.

IR (ATR): 1613 (m), 1586 (m), and 1495 (m, arom. C=C), no signal at 1630 (for CH=N).

17α-[*(E)*-3-hydroxphenylcarbaldimino]-3-methoxy-estra-1,3,5(10)-triene **1d**

Procedure 1. $C_{26}H_{31}NO_2$ (389.55). The crude product precipitated from the reaction mixture at 4 °C was uniform (TLC), 78% after drying. It was directly applied for the next step; mp 165-167 °C, yield 78%, pale yellow powder, TLC: R_f = 0.47 (uniform).

17α-(3-hydroxyphenylmethylamino)-3-methoxy-estra-1,3,5(10)-triene **1e**

Procedure 2. $C_{26}H_{33}NO_2$ (391.55), mp: 128-129 °C (raw product), yield 91%, TLC: R_f = 0.19 (uniform).

Analysis (calcd., found): C (79.76, 78.38), H (8.50, 8.05), N (3.58, 3.44). $[\alpha]_D$ = -116.3 (c = 8.432). ESI-MS: $[MH]^+$ = 392.3 (100%); HRMS (m/z): $[M^+]$ calcd. for $C_{26}H_{34}NO_2$, 392.25895; found, 392.25835.

^{1}H NMR (250 MHz, DMSO-D_6): δ 0.668 (s, 3H, 18-H_3), 1.000-2.400 (m, overl, 13H, 13 x aliph. H), 2.587 (d, ^3J = 6.7 Hz, 1H, 17β-H), 2.771 (s, broad, 2H, 6-H_2), 3.513 (d, ^2J = 13.8 Hz, 1H, N-benzyl-H), 3.634 (d, overl., ^2J = 13.8 Hz, 1H, N-benzyl-H), 3.673 (s, overl., 3H, 3-CH_3O), 6.590 (s, 1H, 4-H), 6.630-6.748 (m, overl., 3H, 2-H and 2x Ar-H), 7.059 (t, ^3J = 7.8 Hz, 1H, Ar-H), 7.164 (d, ^3J = 8.5 Hz, 1H, 1-H), 9.214 (s, 1H, X-H).

^{13}C NMR (73 MHz, DMSO-D_6): δ 19.93, 24.87, 26.61, 28.24, 29.85, 30.07, 32.92, 43.69, 45.32, 48.61, 52.57, 55.31, 66.59, 111.91, 113.76, 113.86, 115.15, 118.88, 126.64, 129.38, 132.73, 137.89, 143.37, 157.46, 157.66.

IR (ATR): 16148 (m), 1582 (s), and 1485 (m, arom. C=C).

17α-[(*E)*-4-hydroxphenylcarbaldimino]-3-methoxy-estra-1,3,5(10)-triene **1f**

Procedure 1. $C_{26}H_{31}NO_2$ (389.55). Yellow oil after evaporation of the solvent. The crude product was applied for the next step; yield 100%, TLC: R_f = 0.45 (purity > 95%).

17α-(4-hydroxyphenyl-methylamino)-3-methoxy-estra-1,3,5(10)-triene **1g**

Procedure 2. $C_{26}H_{33}NO_2$ (391.55), mp: 143-145 °C (from EtOH/water = 9 : 1 v/v), yield 46%, amorphous white powder, TLC: R_f = 0.19 (uniform).

ESI-MS: $[MH]^+$ = 392.4 (100%); HRMS (m/z): $[M^+]$ calcd. for $C_{26}H_{34}NO_2$: 392.25895; found, 392.25853, analysis (calcd., found): C (79.76, 79.64), H (8.50, 8.40), N (3.58, 3.58). $[\alpha]_D$ = -116.3 (c = 8.432).

^{1}H NMR (250 MHz, $CDCl_3$): δ 0.759 (s, 3H, 18-H_3), 1.284-2.000 (m, overl, approx. 13H, 13 x aliph. H), 2.772 (d, overl., ^3J = 6.8 Hz, 1H, 17β-H), 2.843 (s, broad, 2H, 6-H_2), 3.593 (d, ^2J = 12.8 Hz, 1H, N-benzyl-H), approx. 3.740 (d, overl., ^2J = 12.8 Hz, 1H, N-benzyl-H), 3.778 (s, 3H, 3-CH^3O), 6.634-6.722 (s and d, overl., ^3J = 8.4 Hz, 4H, 4-H, 2-H and 2x Ar-H), 7.128-7.263 (d and m, overl., ^3J = 8.4 Hz, 1-H and 2x Ar-H).

^{13}C NMR (63 MHz, $CDCl_3$): δ 19.12, 25.04, 26.43, 28.03, 29.93, 30.08, 32.86, 39.24, 43.42, 45.06, 48.92, 52.30, 55.20, 67.03, 111.42, 113.76, 115.11, 115.53, 126.30, 129.49, 132.16, 132.92, 138.09, 155.01, 157.37.

IR (ATR): 1611 (m), 1599 (m), 1575 (w), and 1498 (s, arom. C=C).

17α-[*(E)*-2-methoxyphenylcarbaldimino]-3-methoxy-estra-1,3,5(10)-triene **1h**

Procedure 1. $C_{27}H_{33}NO_2$ (403.58). The crude product precipitated from reaction mixture at -18 °C was uniform (TLC). It was directly applied for the next step; mp: 133-134 °C, yield 88%, pale yellow amorphous powder, TLC: R_f = 0.69.

17α-(2-methoxyphenyl-methylamino)-3-methoxy-estra-1,3,5(10)-triene **1i**

Procedure 2. $C_{27}H_{35}NO_2$ (405.58), very slowly crystalizing colourless oil after precipitation from the reaction mixture with water at 4 °C (yield about 100%), after recrystallization from hexane or MeOH: mp: 71-72 °C, yield 52%, TLC: R_f = 0.42 (uniform).

Analysis (calcd., found): C (79.96, 80.38), H (8.70, 8.62), N (3.45, 3.44). ESI-MS: $[MH]^+$ = 406.3 (100%); HRMS (m/z): $[M^+]$ calcd. for $C_{27}H_{36}NO_2$, 406.27460; found, 406.27527.

^{1}H NMR (400 MHz, $CDCl_3$): δ 0.747 (s, 3H, 18-H_3), 1.270-2.348 (m, overl, approx. 13H, 13 x aliphatic H), 2.702 (d, overl., ^3J = 6.7 Hz, 1H, 17β-H), 2.847 (s, broad, 2H, 6-H_2), 3.705 (d, ^2J

= 13.6 Hz, 1H, N-benzyl-H), 3.789 (s, 3H, 3-CH_3O), approx. 3.800 (d, overl., ^2J = 13.6 Hz, 1H, N-benzyl-H), 3.870 (s, 3H, 2-CH_3O-benzyl), 6.638 (s, 1H, 4-H), 6.712 (d, ^3J = 8.6 Hz, 1H, 2-H), 6.876-6.934 (m, overl., 1-H and phenyl-H), 7.219-7276 (m, overl. 3H, 3x Ar-H).

^{13}C NMR (100 MHz, $CDCl_3$): δ 19.02, 25.04, 26.48, 28.06, 29.97, 30.10, 32.69, 39.34, 43.46, 43.68, 45.05, 48.23, 48.79, 55.20, 55.25, 66.37, 110.31, 111.43, 113.80, 120.45, 126.27, 128.01, 129.82, 133.07, 138.14, 157.42, 157.77.

IR (ATR): 1607 (m), 1589 (w), 1500 (w), and 1067 (m, arom. C=C).

17α-[*(E)*-4-methoxyphenylcarbaldimino]-3-methoxy-estra-1,3,5(10)-triene **1j**

$C_{27}H_{33}NO_2$ (403.58). This compound was not isolated.

17α-(4-methoxyphenyl-methylamino)-3-methoxy-estra-1,3,5(10)-triene **1k**

Procedure 3. $C_{27}H_{35}NO_2$ (405.58), uniform colourless oil, yield about 100%, TLC: R_f = 0.51. Analysis (calcd., found): C (79.96, 79.61), H (8.70, 8.47), N (3.45, 3.45). ESI-MS: $[MH]^+$ = 406.1 (100%); HRMS (m/z): $[M^+]$ calcd. for $C_{27}H_{36}NO_2$, 406.27460; found, 406.27530.

^1H NMR (250 MHz, $CDCl_3$): δ 0.746 (s, 3H, 18-H_3), 1.235-2.371 (m, overl., approx. 13H, aliph. H), 2.733 (d, overl., ^3J = 5.6 Hz, 1H, 17β-H), 2.865 (s, broad, 2H, 6-H_2), 3.361 (d, ^2J = 13.0 Hz, 1H, N-benzyl-H), approx. 3.740 (d, overl., ^2J = 13.0 Hz, 1H, N-benzyl-H), 3.778 (s, 3H, 3-MeO), 3.805 (s, 3H, CH_3O-Ar), 6.637 (s, 1H, 4-H), 6.703 (d, ^3J = 8.4 Hz, 1H, 2-H), 6.868 (d, ^3J = 8.6 Hz, 2H, 2x Ar-H), 7.205-7.264 (2x d, overl., ^3J = 8.4 and 8.6 Hz, 2H, 1-H and 2x Ar-H).

^{13}C NMR (63 MHz, $CDCl_3$) : δ 19.03, 24.97, 26.46, 28.08, 29.96, 30.30, 32.86, 39.28, 43.49, 45.13, 48.85, 52.27, 55.19, 55.26, 66.77, 111.41, 113.77, 126.31, 129.15, 132.98, 133.19, 138.10, 157.38, 158.50.

IR (ATR): 1607 (m), 1577 (w), 1510 (s), and 1500 (s, arom. C=C).

17α-[*(E)*-2-hydroxynaphth-1-yl-carbaldimino]-3-methoxy-estra-1,3,5(10)-triene **1l**

Procedure 1. $C_{30}H_{33}NO_2$ (439.60), mp: 134-136 °C (from EtOH), yellow crystals, yield 48%, TLC: R_f = 0.64 (uniform).

Analysis (calcd., found): C (81.97, 82.02), H (7.57, 7.34), N (3.19, 2.81).

IR (ATR): 1628 (s, C=N), 1613 (s), 1560 (w) and 1506 (s, arom. C=C).

17α-[(2-hydroxynaphth-1-yl)-methylamino]-3-methoxy-estra-1,3,5(10)-triene **1m**:

Procedure 2. $C_{30}H_{35}NO_2$ (441.61), mp: 105-106 °C (from EtOH, at 4 °C and subsequent addition of about 10% H_2O), small white crystals, yield 33%. TLC: R_f = 0.58 (uniform).

Analysis (calcd., found): C (81.59, 81.09), H (7.99, 7.88), N (3.17, 2.87). ESI-MS: $[MH]^+$ = 442.4 (100%).

^1H NMR (250 MHz, $CDCl_3$): δ 0.858 (s, 3H, 18-H_3), 2.875 (m_c, broad, 2H, 6-H_2), 3.796 (s, 3H, 3-CH_3O), 4.373 (d, ^2J = 9.3 Hz, 2H, benzyl-H), 4.483 (d, ^2J = 9.3 Hz, 2H, benzyl-H), 6.654 (s, 1H, 4-H), 6.736 (d, ^3J = 4.7 Hz, 1H, 2-H), 7.172 (d, ^3J = 4.7 Hz, 1H, 1-H), 7.200-7.480 (m, overl., approx. 6H, 6 x naphthyl-H).

IR (ATR): 1622 (m), 1607 (m), 1583 (m), 1498 (w), and 1466 (m, arom. C=C).

17α-[*(E)*-3-hydroxy-naphth-2-carbaldimino]-3-methoxy-estra-1,3,5(10)-triene **1n**

Procedure 1 using EtOH/THF (2:1 v/v) as solvent, aldehyde was first dissolved in THF. $C_{30}H_{33}NO_2$ (439.60), the uniform product precipitates readily from the reaction mixture and was used for the next step, TLC: R_f = 0.82 (uniform), yield 77%, pale yellow powder.

Analytical sample from EtOH/THF (1:1 v/v): mp: 246-248 °C, yellow crystals.

Analysis (calcd., found): C (81.97, 82.24), H (7.57, 7.63), N (3.19, 3.22). ESI-MS: $[MH]^+$ = 440.2 (100%); HRMS (m/z): $[M^+]$ calcd. for $C_{30}H_{34}NO_2$, 440.25589; found, 406.25723.

^1H NMR (400 MHz, $CDCl_3$): δ 0.93 (s, 3H, 18-H_3), 1.35-1.60 (m, overl., 7H, 7 x aliph. H), 1.55 (m, 1H, aliph. H), 1.71 (m, 1H, aliph. H), 1.98 (m, 2H, 2x aliph. H), 2.10 (s, sh,1H, aliph. H), 2.91 (s, 2H, 6-H_2), 3.45 (s, 1H, 17β-H), 3.80 (s, 3H, 3-CH_3O), 6.67 (s, 1H, 4-H), 6.73 (d, ^3J = 8.0 Hz, 1H, 2-H), 7.23 (d, ^3J = 8.0 Hz, 1H, 1-H), 7.32 (m, 2H, 2x Ar-H), 7.48 (s, 1H, Ar-H), 7.71 (s, 1H, Ar-H), 7.82 (s, 2H, 2x Ar-H), 8.46 (s, 1H, CH=N), 13.23 (s, 1H, OH).

^{13}C NMR (63 MHz, $CDCl_3$): δ 18.55, 25.20, 26.37, 28.11, 29.99, 31.68, 33.40, 39.37, 43.52, 46.25, 49.83, 55.20, 79.19, 110.80, 111.46, 113.78, 121.19, 123.38, 126.33, 126.39, 127.39, 127.97, 128.32, 132.67, 132.77, 135.71, 138.03, 156.96, 157.41, 161.99.
IR (ATR): 1625-1628 (s, broad; C=N overl. with C=C), 1615 (sh, m), 1583 (m), 1518, and 1496 (m, arom. C=C).

17α-[(3-hydroxy-naphth-2-yl)-methylamino]-3-methoxy-estra-1,3,5(10)-triene **1o**
Procedure 2. $C_{30}H_{35}NO_2$ (441.61), mp: 196-198 °C (from EtOH/THF = 1:1 v/v, 4 °C, then addition of about 10% water), yield 90%, TLC: R_f = 0.60 (uniform).
Analysis (calcd., found): C (81.59, 82.03), H (7.99, 7.96), N (3.17, 3.18). ESI-MS: $[MH]^+$ = 442.3 (100%). $[\alpha]_D$ -116.3 ° (c = 8.432).
^1H NMR (250 MHz, $CDCl_3$): δ 0.814 (s, 3H, 18-H_3), 1.293-2.291 (m, overl., approx. 13H, aliph. H), 2.706 (t, ^3J = 8.6 Hz, 1H, 17α-H), 2.850 (s, broad, 2H, 6-H_2), 3.784 (s, 3H, 3-CH_3O), 4.130 (d, ^2J = 13.7 Hz, 1H, Benzyl-H), 4.273 (d, ^2J = 13.7 Hz, 1H, benzyl-H), 6.639 (s, broad, 1H, 4-H), 6.717 (d, ^3J = 8.2 Hz, 1H, 2-H), 7.204-7.308 (m, overl. 3H, 1-H and 2x naphthyl-H), 7.390 (t, ^3J = 7.8 Hz, 1H, naphthyl-H), 7.508 (s, 1H, naphthyl-H), 7.692 (d, ^3J = 8.1 Hz, 2H, 2x naphthyl-H).
^{13}C NMR (63 MHz, $CDCl_3$): δ 11.88, 23.46, 26.29, 27.37, 28.82, 29.76, 37.50, 38.71, 42.92, 43.94, 51.96, 51.99, 55.20, 67.69, 110.61, 111.46, 113.82, 123.03, 125.76, 125.93, 126.19, 126.29, 127.16, 127.26, 127.98, 132.51, 134.52, 137.89, 156.50, 157.47.
IR (ATR): 1640 (w), 1614 (w), 1609 (w), 1577 (m), 1510 (m) and 1491 (m, arom. C=C).

17α-[*(E)*-5-chloro-2-hydroxyphenylcarbaldimino]-3-methoxy-estra-1,3,5(10)-triene **1r**
$C_{26}H_{30}NO_2Cl$ (423.98). This compound was not isolated.

17α-(5-chloro-2-hydroxyphenyl-methylamino)-3-methoxy-estra-1,3,5(10)-triene **1s**

Procedure 3. $C_{26}H_{32}NO_2Cl$ (426.00). The waxy raw product was purified with column chromatography (silica gel, EtOAc/hexane, gradient 1:8 to 1:2 v/v), colourless oil, yield 38%, TLC: R_f = 0.61.

Analysis (calcd., found): C (73.31, 73.26), H (7.57, 7.52), N (3.29, 3.18). ESI-MS: $[MH]^+$ = 426.2 (100%).

^{1}H NMR (250 MHz, $CDCl_3$): δ 0.801 (s, 3H, 18-H_3), 1.263-2.305 (m, overl., ca. 13H, 13 x aliphatic H), 2.805 and 2.833 (m, broad, 3H, 6-H_2 and 17β-H), 3.775 (s, 3H, 3-CH_3O), 4.829 (d, ^2J = 14.0 Hz, 1H, N-benzyl-H), 4.142 (d, ^2J = 14.0 Hz, 1H, N-benzyl-H), 6.626 (s, 1H, 4-H), 6.692 (d, ^3J = 8.2 Hz, 1H, 2-H),), 6.942 (d, ^3J = 8.5 Hz, 2H, 2x phenyl-H), 7.026 (s, 1H, phenyl-H), 7.143 (d, ^3J = 8.2 Hz, 1H, 1-H).

^{13}C NMR (63 MHz, $CDCl_3$): δ 19.08, 24.84, 26.16, 27.96, 28.18, 29.75, 32.60, 32.72, 39.11, 43.31, 44.83, 49.51, 55.17, 66.25, 111.51, 113.80, 118.00, 124.05, 126.28, 128.73, 129.15, 129.28, 132.11, 137.80, 156.10, 157.50.

IR (ATR): 1610 (m), 1578 (sh, m), 1497 (m, arom. C=C); 1482 (s), and 1464 (s).

17β-[*(E)*-2-hydroxphenylcarbaldimino)-3-methoxy-estra-1,3,5(10)-triene **2b**

Procedure 1. $C_{26}H_{31}NO_2$ (389.54), mp: 85-88 °C (from MeOH), yield 86%, yellow needles, TLC: R_f = 0.56 (uniform).

EA: calc. C 80.17, H 8.02, N 3.60; found: C 80.21, H 8.15, N 3.64. $[\alpha]_D$ +105.8 (c = 9.837).

^{1}H NMR (250 MHz, $CDCl_3$): δ 0.864 (s, 3H, 18-H_3), 2.876 (s, broad, 2H, 6-H_2), 3.222 (t, ^3J = 7.5 Hz, 1H, 17α-H), 3.764 (s, 3H, 3-CH_3O), 6.672 (m, 2H, 2- and 4-H), 6.910 (m, 2H, 3- and 5-phenyl-H), 7.256 (m, 3H, 1-H and 4-H and 6-phenyl-H), 8.273 (s, 1H, N=CH), 13.866 (s, 1H, Ar-OH).

^{13}C NMR (63 MHz, $CDCl_3$, DEPT 135): δ 13.0 (18-CH_3), 24.3 (15-C), 26.1 ((11-C), 27.7 (7-C), 29.8 (6-C), 29.9 (12-C), 36.9 (16-C), 55.2 (CH_3O), 79.6 (17-C), 111.4 (2-C), 113.9 (4-C), 126.3 (1-C), 132.5 (10-C), 137.9 (5-C), 157.4 (3-C), 163.1 (CH=N); phenyl-C: 117.0, 118.3, 118.9, 131.0, 132.0, 161.5.

IR (ATR): 1630 (s, C=N valence); 1610 (m), 1579 (w), and 1497 (s, arom. C=C).

17β-(2-hydroxyphenyl-methylamino)-3-methoxy-estra-1,3,5(10)-triene **2c**

Procedure 2. $C_{26}H_{33}NO_2$ (391.55), mp: 117.5-119.5 °C (from methylene chloride/hexane), white plates, yield 71%, TLC: R_f = 0.29 (uniform).

ESI-MS: $[MH]^+$ = 392.2 (100), HRMS (m/z): $[M^+]$ calcd. for $C_{26}H_{34}NO_2$, 392.25895; found, 392.25849.

Analysis (calcd., found): C (79.74, 79.52), H (8.49, 8.64), N (3.58, 3.61). $[\alpha]_D$ +45.9 ° (c = 9.190).

^{1}H NMR (400 MHz, $CDCl_3$): δ 0.789 (s, 3H, 18-H_3), 1.170-2.332 (m, overl., approx. 13H, 13x aliph. H), 2.720 (t, ^3J = 8.4 Hz, 1H, 17α-H), 2.847 (s, broad, 2H, 6-H_2), 3.780 (s, 3H, 3-CH_3O), 3.959 (d, ^2J = 15.1 Hz, 1H, benzyl-H), 4.106 (d, ^2J = 15.1 Hz, 1H, benzyl-H), 6.627 (m, 1H, 4-H), 6.694-6.861 (m, overl. 3H, 1-H and 2x Ar-H), 7.001 (d, ^3J = 7.5 Hz, 1H, Ar-H), 7.184 (m, 2H, 2x Ar-H).

^{13}C NMR (100 MHz, $CDCl_3$, DEPT 135): δ 11.86 (18-C), 23.46 (15-C), 26.29 (11-C), 27.38 (7-C), 28.84 (6-C), 29.76 (12-C), 37.56 (16-C), 38.72, 42.92, 43.96, 51.68 (benzyl-C), 52.02, 55.19 (OCH_3), 67.78 (17-C), 111.46 (2-C), 113.82 (4-C), 116.43 (aryl-C), 118.90 (aryl-C), 122.94 (aryl-C), 126.28 (1-C), 128.10 (aryl-C), 128.68 (aryl-C), 132.53 (10.C), 137.89 (5-C), 157.47 (3-C), 158.47 (aryl-C).

IR (ATR):1612 (m), 1589 (w), and 1496 (s, arom. C=C), no C=N valence at 1630.

17β-[*(E)*-3-hydroxyphenyl-carbaldimino]-3-methoxy-estra-1,3,5(10)-triene **2d**

$C_{26}H_{31}NO_2$ (389.54). This compound was not isolated.

17β-(3-hydroxyphenyl-methylamino)-3-methoxy-estra-1,3,5(10)-triene **2e**

Procedure 3. $C_{26}H_{33}NO_2$ (391.55), mp: 184-187 (from EtOH/THF, 4:1 v/v), 69%, colourless amorphous compound, TLC: R_f = 0.90, with solvent AcOEt/hexane 1:4 (v/v): R_f = 0.48 (uniform).

Analysis (calcd., found): C (79.76, 79.74), H (8.50, 8.62), N (3.58, 3.59). ESI-MS: $[MH]^+$ = 392.2 (100%); HRMS (m/z): $[M^+]$ calcd. for $C_{26}H_{34}NO_2$, 392.25895; found, 392.25896.

^{1}H NMR (250 MHz, $CDCl_3$; broad signals): δ 0.795 (s, 3H, 18-H_3), 1.100-2.350 (m, overl., approx. 13H, aliph. H), 2.704 (t, ^{3}J approx. 7 Hz, 1H, 17α-H), 2.482 (s, very broad, approx. 4H, 6-H_2 and 2x N-benzyl-H), 3.779 (s, 3H, 3-CH_3O), 6.663 (s, 1H, 4-H), 6.700 (s, broad, overl., 2H, 2-H and phenyl-H), 6.799-6.869 (m, overl., 2H, 2x Ar-H), 7.181 (m, overl., 2x Ar-H).

^{13}C NMR (63 MHz, $CDCl_3$): δ 11.92, 23.50, 26.50, 27.42, 29.54, 29.82, 38.12, 38.79, 43.17, 43.99, 52.28, 52.44, 55.19, 68.30, 111.43, 113.79, 114.05, 115.17, 120.17, 126.27, 129.50, 132.76, 137.99, 142.43, 155.96, 157.40.

IR (ATR): 1613 (m), 1586 (m-s), 1577 (m), and 1497 (m, arom. C=C).

17β-[*(E)*-2-hydroxynaphth-1-yl-carbaldimino]-3-methoxy-estra-1,3,5(10)-triene **2l**

$C_{30}H_{33}NO_2$ (339.60). This compound (oil) was not isolated.

17β-[(2-hydroxynaphth-1-yl)-methylamino]-3-methoxy-estra-1,3,5(10)-triene hydroperchlorate **2m**

Procedure 3. The raw product was chromatographed on silica gel (eluent AcOEt/hexane, gradient 1:7 to 1:4 v/v). The resulting oil was dissolved in THF, followed by the addition of 2 equivalents of perchloric acid and precipitation of the resulting salt with water at 4 °C.

$C_{30}H_{36}NO_6Cl$ (542.07), mp: 138-143 °C (decomp.), ochre flakes, yield 23%, TLC: R_f = 0.50 (uniform).

Analysis (calcd., found): C (66.47, 66.28), H (6.69, 6.99), N (2.58, 2.50), Cl (6.54, 5.90). ESI-MS: 442.4 $[M - ClO_4]^+$.

^{1}H NMR (250 MHz, $CDCl_3$; broad signals, selected data): δ 0.896 (s, 3H, 18-H_3), 2.770 (s, broad, 2H, 6-H_2), 3.752 (s, 3H, 3-CH_3O), 4.716 (m, 2H, CH_2-N), 8.455 (s, broad, 1H, X-H).

^{13}C NMR (63 MHz, $CDCl_3$): δ 11.51, 23.21, 25.15, 25.76, 27.08, 29.45, 35.97, 38.16, 38.45, 42.73, 43.29, 43.74, 51.25, 55.19, 66.89, 107.72, 111.54, 113.82, 117.64, 121.26, 123.83, 126.24, 127.98, 129.07, 131.64, 132.03, 132.44, 137.52, 153.71, 157.54.

IR (ATR): 1629 (w), 1609 (m), 1584 (m), 1577 (m), 1518 (m), and 1500 (m-s, arom. C=C), 1038-1089 (vs, broad).

17β-[*(E)*-3-hydroxy-naphth-2-carbaldimino]-3-methoxy-estra-1,3,5(10)-triene **2n**

Procedure 1 (solvent EtOH/THF = 1:1 v/v, room temperature; alternatively in EtOH at 40 °C). $C_{30}H_{33}NO_2$ (339.60), mp: 234.5-236.5 °C (from EtOH/THF = 1:1 v/v), yellow needles, yield 91% (crude product about 100% yield), TLC: R_f = 0.76 (uniform).

Analysis (calcd., found): C (81.97, 82.07), H (7.57, 7.67), N (3.19, 3.22).

^1H NMR (250 MHz, $CDCl_3$; selected data): δ 0.960 (s, 3H, 18-H_3), 2.880 (s, (s, broad, 1H, OH)., 2H, 6-H_2), 3.296 (t, ^3J = 8.6 Hz, 1H, 17α-H), 3.782 (s, 3H, 3-CH_3O), 6.661 (m, 1H, 4-H), 6.780 (d, ^3J = 7.5 Hz, 1H, 2-H), 7.264 (d, ^3J = 7.5 Hz, 1H, 1-H), 7.304-7.769 (m, approx. 6H, 6x naphthyl-H), 8.530 (s, 1H, N=CH), 13.303 (s, broad, 1H, OH).

^{13}C NMR (63 MHz, $CDCl_3$): δ 13.11, 24.37, 26.13, 27.71, 29.85, 30.06, 36.98, 38.79, 43.94, 45.45, 52.53, 55.20, 80.09, 110.84, 111.47, 113.84, 121.30, 123.30, 126.31, 127.29, 127.92, 128.31, 132.51, 132.57, 133.75, 137.94, 157.11, 157.49, 163.14.

IR (ATR): 1629 and 1624 (s, overl.; C=N and C=C), 1610 (m), 1570 (m), 1517 (m), 1497 (w) and 1473 (m-s, arom. C=C).

17β-[(3-hydroxynaphth-2-yl)-methylamino]-3-methoxy-estra-1,3,5(10)-triene **2o**

Procedure 2 (solvent EtOH/THF = 1:1 v/v, 40 °C). $C_{30}H_{35}NO_2$ (441.61), mp: 192-194 °C (from EtOH/THF, 1:1 v/v, finally addition of about 10% H_2O), fine white crystals, yield 67%, TLC: R_f = 0.40 (uniform). Advantageously, synthesis can be performed as one-pot synthesis following procedure 3 (solvent EtOH/THF = 1:1 v/v): Uniform crude product, yield about 100%.

Analysis (calcd., found): C (81.59, 81.48), H (7.99, 7.92), N (3.17, 3.18). ESI-MS: $[MH]^+$ = 442.4 (100%).

^{1}H NMR (250 MHz, $CDCl_3$): δ 0.814 (s, 3H, 18-H_3), 1.293-2.291 (m, overl., approx. 13H, 13 x aliph. H), 2.706 (t, ^{3}J = 8.6 Hz, 1H, 17α-H), 2.850 (s, broad, 2H, 6-H_2), 3.784 (s, 3H, 3-CH_3O), 4.130 (d, ^{2}J = 13.7 Hz, 1H, benzyl-H), 4.273 (d, ^{2}J = 13.7 Hz, 1H, benzyl-H), 6.639 (s, broad, 1H, 4-H), 6.717 (d, ^{3}J = 8.2 Hz, 1H, 2-H), 7.204-7.308 (m, overl. 3H, 1-H and 2x naphthyl-H), 7.390 (t, ^{3}J = 7.8 Hz, 1H, naphthyl-H), 7.508 (s, 1H, naphthyl-H), 7.692 (d, ^{3}J = 8.1 Hz, 2H, 2x naphthyl-H).

^{13}C NMR (63 MHz, $CDCl_3$): δ 11.88, 23.46, 26.29, 27.37, 28.82, 29.76, 37.50, 38.71, 42.92, 43.94, 51.96, 51.99, 55.20, 67.69, 110.61, 111.46, 113.82, 123.03, 125.76, 125.93, 126.19, 126.29, 127.16, 127.26, 127.98, 132.51, 134.52, 137.89, 156.50, 157.47.

IR (ATR): 1637 (s, C=N), 1611 (m), 1574 (m), and 1498 (m, arom. C=C).

17β-[*(E)*-4-N,N-diethylamino-2-hydroxyphenyl-carbaldimino]-3-methoxy-estra-1,3,5(10)-triene **2p**

Procedure 1. $C_{30}H_{40}N_2O_2$ (460.65), mp: 234.5-235.5 °C (from EtOH), pale yellow crystals, yield 88%, TLC: R_f = 0.48 (uniform).

^{1}H NMR (250 MHz, $CDCl_3$): δ 0.890 (s, 3H, 18-H_3), 1.190 (t, ^{3}J = 7.0 Hz, 6H, 2xC$\underline{H}_3$-CH_2), 1.200-2.311 (m, overl., 13H), 2.862 (s, broad, 2H, 6-H_2), 3.173 (t, ^{3}J = 8.7 Hz, 1H, 17α-H), 3.373 (q, ^{3}J = 7.0 Hz, 4H, 2xCH_3-C$\underline{H}_2$), 3.780 (s, 3H, 3-CH_3O), 6.084 (s, 1H, phenyl-H), 6.138 (d, ^{3}J = 8.4 Hz, 1H, phenyl-H), 6.628 (d, ^{3}J = 8.4 Hz, 1H, phenyl-H), 6.643 (s, 1H, 4-H), 6.69 (d, ^{3}J = 8.6 Hz, 1H, 2-H), 7.204 (d, ^{3}J = 8.6 Hz, 1H, 1-H), 7.957 (s, 1H, CH=N), 14.260 (s, broad, 1H, OH).

^{13}C NMR (63 MHz, $CDCl_3$): δ12.70, 12.75, 24.02, 25.60, 26.13, 27.61, 29.66, 29.84, 36.88, 38.86, 43.95, 44.49, 44.74, 52.23, 55.19, 76.87, 77.20, 98.65, 102.90, 108.36, 111.44, 113.80, 126.30, 132.58, 132.69, 137.94, 151.76, 157.45, 161.03, 168.04.

IR (ATR): 1613 (vs, C=N); 1563 (m), 1520 (s), and 1498 (m, arom. C=C).

17β-[4-(N,N-diethylamino)-2-hydroxyphenyl-methylamino]-3-methoxy-estra-1,3,5(10)-triene **2q**

Procedure 2, in EtOH/THF (2:1 v/v) at 40 °C. $C_{30}H_{42}N_2O_2$ (462.67), mp: 137-142 °C, amorphous off-white raw product, yield 92%, TLC: R_f = 0.30 (uniform).

ESI-MS: $[MH]^+$ = 463.3 (100%).

^{1}H NMR (250 MHz, $CDCl_3$; very broad signals): δ 0.773 (s, 3H, 18-H_3), 1.157 (s, overl., 6H, 2xCH_3), 2.851 (s, broad, 2H, 6-H_2), 3.310 (s, 4H, 2xCH_2N), 3.783 (s, 3H, 3-CH_3O).

^{13}C NMR (63 MHz, $CDCl_3$): δ 11.20, 12.10, 23.49, 28.86, 29.78, 30.31, 31.17, 37.57, 38.73, 42.89, 44.37, 44.47, 51.05, 52.21, 55.19, 67.54, 99.37, 102.73, 110.18, 111.44, 113.80, 126.30, 128.69, 132.74, 138.02, 148.78, 157.44, 159.37.

IR (ATR): 1629 (m-s), 1610 (m-s), 1560 (m), 1518 (s), and 1500 (s, arom. C=C).

17β-[*(E)*-5-chloro-2-hydroxyphenylcarbaldimino]-3-methoxy-estra-1,3,5(10)-triene **2r**

Procedure 1. $C_{26}H_{30}NO_2Cl$ (423.98), mp: 146.5-147.5 °C, citreous crystals, yield 88%, TLC: R_f = 0.87 (uniform). This product was further used.

17β-(5-chloro-2-hydroxyphenyl-methylamino)-3-methoxy-estra-1,3,5(10)-triene **2s**

Procedure 2. $C_{26}H_{32}NO_2Cl$ (426.00), mp: 161-163 (from EtOH), colourless plates, yield 47%, TLC: R_f = 0.69 (uniform).

Analysis (calcd., found): C (73.31, 73.34), H (7.57, 7.64), N (3.29, 3.29), Cl (8.32, 8.20). ESI-MS: $[MH]^+$ = 426.2 (100%).

^{1}H NMR (250 MHz, $CDCl_3$): δ 0.812 (s, 3H, 18-H_3), 1.153-2.280 (m, overl, ca. 13H, 13 x aliphatic H), 2.722 (t, ^3J = 8.25 Hz, 1H, 17α-H), 2.843 (m, broad, 2H, 6-H_2), 3.776 (s, 3H, 3-CH_3O), 3.932 (d, ^2J = 14.0 Hz, 1H, N-benzyl-H), 4.105 (d, ^2J = 14.0 Hz, 1H, N-benzyl-H), 6.632 (s, 1H, 4-H), 6.708 (d, ^3J = 8.7 Hz, 1H, 2-H),), 6.846 (d, ^3J = 8.5 Hz, 2H, 2x Ar-H), 7.003 (s, 1H, Ar-H), 7.108-7.203 (m, overl., 2H, 1-H and Ar-H).

IR (ATR): 1611 (m), 1577 (m-w), 1480 (m, sh), and 1462 (s, arom. C=C).

17β-[*(E)*-2-hydroxy-5-nitrophenyl-carbaldimino)-3-methoxy-estra-1,3,5(10)-triene **2t**

Procedure 1. $C_{26}H_{30}N_2O_4$ (434.54), mp: 204-206 °C, orange crystals, yield 85%, TLC: R_f = 0.62 (uniform). The crude product was further applied.

17β-(2-hydroxy-5-nitrophenyl-methylamino)-3-methoxy-estra-1,3,5(10)-triene **2u**

Procedure 2. $C_{26}H_{32}N_2O_4$ (436.55), mp: 212-215 °C (from EtOH/THF 1:1 v/v), yellowish amorphous powder, yield 40%, TLC: R_f = 0.14 (uniform).

ESI-MS: $[MH]^+$ = 437.2 (100), HRMS (m/z): $[M^+]$ calcd. for $C_{26}H_{33}N_2O_4$, 437.24403; found, 437.24411.

^1H NMR (250 MHz, $CDCl_3$): δ 0.814 (s, 3H, 18-H_3), 1.200-2.250 (m, overl, ca. 13H, aliphatic H), 2.717 (t, ^3J = 8.2 Hz, 1H, 17α-H), 2.852 (m, broad, 2H, 6-H_2), 3.778 (s, 3H, 3-CH_3O), 4.050 (d, ^2J = 14.0 Hz, 1H, N-benzyl-H), 4.210 (d, ^2J = 14.0 Hz, 1H, N-benzyl-H), 6.634 (s, 1H, 4-H), 6.732 (d, ^3J = 8.75 Hz, 1H, 2-H), 6.870 (d, ^3J = 9.0 Hz, 1H, Ar-H), 7.197 (d, ^3J = 8.75 Hz, 1H, 1-H), 7.966 (s, 1H Ar-H), 8.090 (d, ^3J = 9.0 Hz, 1H, Ar-H).

^{13}C NMR (63 MHz, $CDCl_3$): δ 11.87, 23.40, 26.21, 27.32, 28.53, 29.71, 36.88, 37.46, 38.65, 43.87, 51.04, 51.94, 55.20, 67.63, 111.49, 113.82, 116.76, 122.48, 124.34, 125.30, 126.26, 132.28, 137.84, 139.94, 157.51, 165.28.

IR (ATR): 1603 (m), 1590 (m), 1572 (m), 1566 (m), and 1498 (m, arom. C=C).

16α-[*(E)*-2-hydroxyphenylcarbaldimino]-3-methoxy-estra-1,3,5(10)-triene **3b**

Procedure 1, $C_{26}H_{31}NO_2$ (389.54), mp: 118-120 (from MeOH), yellow crystals, yield 88%, $[\alpha]_D$ = +126.4 (c = 8.027), TLC: R_f = 0.77 (uniform).

Analysis (calcd., found): C (80.16, 80.01), H (8.02, 8.09), N (3.67, 3.77).

^1H NMR (250 MHz, $CDCl_3$): δ 0.84 (s, 3H, 18-H_3), 3.76 (s, 3H, OCH_3), 3.97 (m, 1H, 16β-H), 6.61-7.30 (7H, 7 x Ar-H), 8.27 (s, 1H, CH=N), 13.68 (s, 1H, -XH).

^{13}C NMR (63 MHz, $CDCl_3$): δ 18.7 (C-18), 26.4 (C-11), 28.0 (C-7), 29.6 (C-6), 35.5 (C-15), 38.4 (C-12), 38.9 (C-8), 42.3 (C-13), 43.8 (C-9), 50.5 (C-17), 52.1 (C-14), 55.2 (OCH_3), 66.9 (C-16), 111.4 (C-2), 113.8 (C-4), 126.2 (C-1), 132.7 (C-10), 137.9 (C-5), 157.4 (C-3), 162.0 (CH=N); Ar-C: 116.9, 118.4, 118.8 (q-C), 131.0, 131.8, 161.1 (q-C).

IR (ATR): 1628 (vs, CH=N), 1613 (m-s), 1581 (m), 1507 (m-s), and 1479 (m, arom. C=C).

16α-(2-hydroxyphenyl-methylamino)-3-methoxy-estra-1,3,5(10)-triene **3c**

Procedure 2. $C_{26}H_{33}NO_2$ (391.55), mp: 148-149.5 °C (from MeOH), white powder, yield 87%, TLC: R_f = 0.30 (uniform), $[\alpha]_D$ = +54.8 (c = 6.780).

Analysis (calcd., found): C (79.75, 79.59), H (8.49, 8.44), N (3.58, 3.59). HRMS (m/z): $[M^+]$ calcd. for $C_{26}H_{33}NO_2$, 391.25113; found, 391.25390.

^{1}H NMR (250 MHz, $CDCl_3$): δ 0.75 (s, 3H, 18-H_3), 3.38 (m, 1H, 16β-H), 3.39 (q, 2H, N-CH_2), 3.76 (s, 3H, OCH_3), 6.61-7.20 (7H, 7 x Ar-H).

^{13}C NMR (250 MHz, $CDCl_3$): δ 18.3 (C-18), 26.3 (C-11), 28.0 (C-7), 29.6 (C-6), 33.0 (C-15), 38.6 (C-12), 38.7 (C-8), 41.6 (C-13), 43.9 (C-9), 49.5 (C-17), 51.7 (C-14), 55.2 (OCH_3), 55.8 (C-16), 111.4 (C-2), 113.8 (C-4), 126.2 (C-1), 132.7 (C-10), 137.9 (C-5), 157.4 (C-3); 51.7 (CH_2-N); Ar-C: 116.4, 118.9, 122.8 (q-C), 128.1, 128.6, 154.4 (q-C).

IR (ATR): 1613 (m),1588 (m), 1574 (w), and 1498 (m, arom. C=C).

16β-[*(E)*-2-hydroxyphenylcarbaldimino]-3-methoxy-estra-1,3,5(10)-triene **4b**

$C_{26}H_{31}NO_2$ (389.54), mp: 181-183 (from MeOH), yellow powder, yield 90%, $[\alpha]_D$ = +24.6 (c = 7.315), TLC: R_f = 0.74 (uniform).

Analysis (calcd., found): C (80.16, 80.39), H (8.02, 8.39), N (3.67, 3.67).

^{1}H NMR: δ 1.02 (s, 3H, 18-H_3), 3.76 (s, 3H, OCH_3), 3.88 (m, 1H, 16α-H), 6.61-7.30 (7H, 7 X Ar-H), 8.25 (s, 1H, CH=N), 13.70 (s, 1H, -XH).

^{13}C NMR: δ 19.1 (C-18), 26.5 (C-11), 28.1 (C-7), 29.8 (C-6), 35.9 (C-15), 38.8 (C-12), 38.6 (C-8), 41.4 (C-13), 43.9 (C-9), 50.0 (C-17), 53.6 (C-14), 55.2 (OCH_3), 67.1 (C-16), 111.5 (C-2), 113.8 (C-4), 126.2 (C-1), 132.7 (C-10), 137.8 (C-5), 157.4 (C-3), 162.1 (CH=N); Ar-C: 116.9, 118.4, 118.8 (q-C), 131.0, 131.9, 161.1 (q-C).

IR (KBr): 1630 (s, CH=N); 1582 (m) and 1503 (m, arom. C=C).

16β-(2-hydroxyphenyl-methylamino)-3-methoxy-estra-1,3,5(10)-triene **4c**

Procedure 2, $C_{26}H_{33}NO_2$ (391.55), mp: 149-151.5 °C (from MeOH), yield 88%, white powder, TLC: R_f = 0.43 (uniform), $[\alpha]_D$ = +61.7 (c = 7.315).

Analysis (calcd., found): C (79.75, 78.70), H (8.49, 8.37), N (3.58, 3.95). HRMS (m/z): $[M^+]$ calcd. for $C_{26}H_{33}NO_2$, 391.25113; found, 391.25171.

^{1}H NMR (250 MHz, $CDCl_3$): δ 0.92 (s, 3H, 18-H_3), 3.29 (m, 1H, 16α-H), 3.93 (q, 2H, N-CH_2), 3.76 (s, 3H, OCH_3), 6.61-7.19 (7H, 7 x Ar-H).

^{13}C NMR (250 MHz, $CDCl_3$): δ 19.9 (C-18), 26.4 (C-11), 28.0 (C-7), 29.8 (C-6), 34.2 (C-15), 38.9 (C-12), 38.4 (C-8), 40.5 (C-13), 43.8 (C-9), 47.6 (C-17), 52.7 (C-14), 55.2 (OCH_3), 56.4 (C-16), 111.5 (C-2), 113.7 (C-4), 126.2 (C-1), 132.6 (C-10), 137.8 (C-5), 157.4 (C-3); 51.6 (CH_2-N); Ar-C: 116.4, 119.0, 122.7 (q-C), 128.2, 128.7, 158.2 (q-C).

IR (KBr): 1608 (m), 1499 (m), and 1472 (m, arom. C=C).

17α-hydroxy-16α-[*(E)*-2-hydroxyphenylcarbaldimino)-3-methoxy-estra-1,3,5(10)-triene **5b** [10]

Also obtained as tautomeric mixture containing both 1,3-oxazolidine C2 epimers.

$C_{26}H_{31}NO_3$ (405.56), mp: 128-132 °C; 72% (crude product), recrystallization from EtOH again yields a mixture with an unchanged ratio of the isomers. This mixture was further used. $[\alpha]_D$ = +103.7 (c = 0.875).

Analysis (calcd., found): C (76.99, 76.57), H (7.71, 7.87), N (3.47, 3.53).

^{1}H NMR (250 MHz, $CDCl_3$): δ 0.72, 078 und 0.84 (3 x s, 3 x 3H, 3 x 18-H_3); 5.38 and 5.98 (2x s, 2x 1 H, 2x CH-N); 8.43 (s, 1H, CH=N).

IR (KBr): 1637 (s, CH=N), 3104 (broad, NH).

17α-hydroxy-16α-(2-hydroxyphenyl-methylamino) -3-methoxy-estra-1,3,5(10)-triene **5c** [10]

Procedure 2 - using mixture of isomers as obtained above. $C_{26}H_{33}NO_3$ (407.6), mp: 176-182 °C, yield 88%. $[\alpha]_D$ = +31.4 (c = 0.921).

Analysis (calcd., found): C (76.62, 76.46), H (8.16, 8.37), N (3.44, 3.51). ESI-MS: M^+ = 408 (100%).

^{1}H NMR (250 MHz, $CDCl_3$): δ 0.71 (s, 3H, 18-H_3); 3.42 (m, 1H, 16-H); 3.76 (s, 3H, OCH_3); 3.81 (d, 1H, 17-H); 3.98 (q, 2H, -CH_2); 6.60-7.20 (m, 7H, 7 x Ar-H).

^{13}C NMR (63 MHz, $CDCl_3$): δ 17.15 (C-18), 51.23 (N-CH_2), 58.01 (C-16), 78.14 (C-17), 116.37, 118.98, 128.42, 128.65 (C_6H_4); 122.72 (H_2C-$\underline{C}_6H_4$); 158.33 (HO-$\underline{C}_6H_4$).

IR (ATR): 1609 (m), 1581 (m-w), and 1490 (s, arom. C=C).

17α-hydroxy-16α-[*(E)*-5-Chloro-2-hydroxyphenylcarbaldimino]-3-methoxy-estra-1,3,5(10)-triene **5r**

Procedure 1. $C_{26}H_{30}NO_3Cl$ (439.99). Precipitation from the reaction mixture at -18 °C furnishes **5r**, yield 85%, mp: 167-169 °C, and bright yellow micro-crystalline compound.

^{1}H NMR (400 MHz, $CDCl_3$): Mixture of the azomethine and oxazolidine epimers (ratio about 2:1:1; estimated by 18-H_3-integrals at δ = 0.767, 0.819 and 0.874 ppm). This compound was not further purified.

17α-hydroxy-16α-(5-Chloro-2-hydroxyphenyl-methylamino9-3-methoxy-estra-1,3,5(10)-triene **5s**

Procedure 2, alternatively Procedure 3. $C_{26}H_{32}NO_3Cl$ (441.99), mp: 258-262 °C (removal of boric complexes by treatment of the crude product with 5% HCl, after neutralization with 5% $NaHCO_3$ solution the product was purified by precipitation several times from a solution in EtOH with water upon adding of one drop of AcOH), amorphous off-white powder, yield 50%, TLC: R_f = 0.01.

ESI-MS: $[MH]^+$ = 442.2 (100%).

^{1}H NMR (250 MHz, DMSO-D_6): δ 0.68 (s, 3H, 18-H_3), 1.20-1.55 (m, overl., 5-H, aliph. H), 1.77 (s, broad, 4 H. aliph. H), 2.10 (s, broad, 1 H, aliph. H), 2.35 (s, broad, 1 H, aliph. H), 2.49 (s, 1H, aliph. H), 2.77 (s, broad, 2H, 6-H_2), 3.20-3.90 (m, overl,, 5H, 16β-H, 17β-H and 3-OCH_3), 4.06 (s, broad, 2H, 6-H_2), 6.59 (s, 1H, 4-H), 5.66 (d, J = 7.5 Hz, 1H, 2-H), 6.99 (d, ^{3}J = 7.5 Hz, 1H, 1-H), 7.20-7.40 (2x d, overl., ^{3}J each 7.5 Hz, 3H, 3 x AR-H), 7.49 (s, 1H, X-H), 8.86 (s, very broad, about 1 H, X'-H).

^{13}C NMR (63 MHz, DMSO-D_6): δ 16.91, 25.83, 28.07, 29.07, 29.65, 31.44, 43.68, 44.35, 45.75, 46.26, 55.33, 58.62, 75.88, 111.94, 113.87, 117.45, 121.01, 122.59, 126.60, 130.12, 131.17. 132.28, 137.75, 146.89, 155.39, 157.51.

IR (ATR): 1608 (m), 1568 (w), and 1494 (s, arom. C=C).

17α-hydroxy-16β-[*(E)*-2-hydroxyphenylcarbaldimino)-3-methoxy-estra-1,3,5(10)-triene **6b** [10]

Procedure 1. $C_{26}H_{31}NO_3$ (405,56), mp: 198-203 °C (from MeOH), yield 90%, yellow powder, $[\alpha]_D$ = +62.7.

Analysis (calcd., found): C (76.99, 77.07), H (7.71, 7.89), N (3.47, 3.39).

^1H NMR: δ 0.96 (s, 3H, 18-H_3); 3.66 (t, ^3J = 7.5 Hz, 1H, 16β-H); 3.76 (s, 3H, OCH_3); 3.78 (s, 1H, 17β-H); 6.62-7.32 (m, 7H, 7 x Ar-H); 8.33 (s, 1H, N=CH);

^{13}C NMR ($CDCl_3$): δ 17.31 (C-18), 77.71 (C-16), 77.71 (16-C); 86.80 (17-C); 116.88, 118.65, 131.22, 132.19 (C_6H_4); 118.72 (C-$\underline{C}_6H_4$); 160.92 (HO-$\underline{C}_6H_4$); 163.55 (N=CH).

IR (KBr): 1628 (s, CH=N).

16β-(2-hydroxyphenyl-methylamino)-17α-hydroxy-3-methoxy-estra-1,3,5(10)-triene **6c** [10]

Procedure 2. $C_{26}H_{33}NO_3$ (407.55), mp: 158-161 °C (from MeOH), colourless plates, yield 94%. $[\alpha]_D$ = +62.0 (c = 0.981).

Analysis (calcd., found): C (76.62, 76.46), H (8.16, 8.37), N (3.44, 3.51). ESI-MS: M^+ = 408 (100%).

^1H NMR (250 MHz, $CDCl_3$): δ 0.88 (s, 3H, 18-H_3); 3.03 (t, 1H, 16-H); 3.70 (s, 1H, 17-H); 3.76 (s, 3H, OCH_3); 4.02 (s, 2H, CH_2); 6.62-7.21 (m, 7H, 7 x Ar-H).

^{13}C NMR (63 MHz, $CDCl_3$): δ 17.87 (C-18), 51.99 (N-CH_2), 67.88 (C-16), 84.88 (C-17), 116.42, 119.24, 128.23, 128.86 (C_6H_4); 122.77 (H_2C-$\underline{C}_6H_4$); 157.96 (HO-$\underline{C}_6H_4$).

IR (ATR): 1617 (w), 1604 (m), 1586 (m-w), 1579 (w, sh), and 1497 (m-s, arom. C=C).

16β-([*(E)*-3,5-di-*tert*-butyl-2-hydroxyphenyl]carbaldimino)-17α-hydroxy-estra-1,3,5(10)-triene **6v**

Procedure 1. $C_{34}H_{47}NO_3$ (517.54), mp: 185-188 °C (from MeOH or heptane), yellow crystals, yield 90%, $[\alpha]_D$ = +45.5.

Analysis (calcd., found): C (78.91, 78.62), H (9.15, 9.16), N (2.71, 2.74).

^{1}H NMR (250 MHz, $CDCl_3$): δ 0.983 (s, 3H, 18-H_3); 1.284 (s, 9H, *tert*-Bu);1.413 (s, 9H, *tert*-Bu); 2.836 (m_c, 2H, 6-H_2); 3.620 (t, ^3J = 8 Hz, 1H, 17β-H); 3.762 (s, 3H, OCH_3); 3.807(s, 1H, 16α-H); 6.618 (s, 1H, 4-H), 6.695 (d, ^3J = 9.4 Hz, 1H, 2-H), 7.066 (s, 1H, Ar-H), 7.222 (d, ^3J = 9.4 Hz, 1H, 1-H), 7.346 (s, 1H, Ar-H), 8.360 (s, 1H, CH=N).

IR (ATR): 2956 and 2916 (broad, m; C-H), 1619 and 1608 (m-s, C=N and arom. C=C), 1596 (m, sh), 1584 (w, sh), and 1503 (m-s, arom. C=C).

16β-([3,5-di-*tert*-butyl-2-hydroxyphenyl]methylamino)-17α-hydroxy-3-methoxy-estra-1,3,5(10)-triene **6w** [10]

Procedure 2. $C_{24}H_{49}NO_4$ (519.76), mp: 184.5-186 °C (from EtOH/water), white powder, yield 82%, TLC: R_f = 0.71 (uniform), $[\alpha]_D$ = +43 (*c* = 0.934).

Analysis (calcd., found): C (78.60, 77.67), H (9.51, 9.93), N (2.70, 2.83). ESI-MS: 519 (71) [M^+], 214 (100). HRMS (m/z): [M^+] calcd. for $C_{24}H_{49}NO_4$, 519.3710; found, 519.3712.

^{1}H NMR (400 MHz, $CDCl_3$, COSYDQF): δ 0.88 (s, 3H, 18-H_3); 1.28 (s, 9H, *tert*-Bu),1.43 (s, 9H, *tert*-Bu), 2.85 (m_c, 2H, 6-H2), 3.09 (t, line form analysis: 2x d with 2x ^3J approx. 8 Hz, 1H, 16α-H), 3.04 (s, 1H, 17β-H), 3.70 (s, 3H, OCH_3), 4.00 (m_c, 2H, 2x benzyl-H); 6.63–7.23 (m, 7H, 7 x Ar-H).

^{13}C NMR (100 MHz, $CDCl_3$): δ 53.03 (s, N-CH_2), 68.29 (s, C-16), 84.77 (s, C-17).

IR (KBr): 3299 (m) and 3569 (s, NH, OH), no CH=N signal.

17β-hydroxy-16α-[*(E)*-2-hydroxyphenylcarbaldimino]-3-methoxy-estra-1,3,5(10)-triene **7b** [10]

Procedure 1. $C_{26}H_{31}NO_3$ (405.56), mp: 169-172 °C (from MeOH), yellow powder, yield 89%, $[\alpha]_D$ = +81.7 (c = 0.969).

Analysis (calcd., found): C (76.99, 76.82), H (7.71, 7.87), N (3.47, 3.54).

^1H NMR (250 MHz, $CDCl_3$): δ 0.89 (s, 3H, 18-H_3); 3.63 (m, 1H, 16β-H); 3.70 (d, ^3J = 6.5 Hz, 1H, 17α-H); 3.77 (s, 3H, OCH_3); 6.62 (d, ^4J = 2.8 Hz, 1H, 4-H); 6.70 (dd, ^3J = 8.5 Hz, ^4J = 2.8 Hz, 1H, 2-H); 6.86 (m, 1H, C_6H_4); 6.94 (m, 1H, Ar-H); 7.17–7.32 (m, 3H, 1-H and Ar-H); 8.31 (s, 1H, N=CH).

^{13}C NMR (63 MHz, $CDCl_3$): δ 12.23 (s, C-18), 25.97 (s, C-11), 27.11 (s, C-7), 29.68 (s, C-6), 32.78 (s, C-15), 36.49 (s, C-12), 38.42 (s, C-8), 43.87 (s, C-9), 43.99 (s, C-13), 48.36 (s, C-14), 55.15 (s, OCH_3), 74.64 (s, C-16), 87.91 (s, C-17), 111.47 (s, C-2), 113.75 (s, C-4), 116.91, 118.59 (23 s, C_6H_4), 118.66 (s, C-C_6H_4), 126.23 (s, C-1), 131.24, 132.12, (2x s, Ar-C), 132.29 (s, C-10), 137.83 (s, C-5), 157.42 (s, C-3), 161.04 (s, HO-Ar), 163.87 (CH=N).

IR (ATR): 3492 (m-s, OH); 2929, 2897, 2871 and 2836 (m, C-H); 1622 (vs, C=N); 1577 (m), and 1498 (s, arom. C=C).

17β-hydroxy-16α-(2-hydroxyphenyl-methylamino)-3-methoxy-estra-1,3,5(10)-triene **7c** [10]

Procedure 2. $C_{26}H_{33}NO_3$ (407.6), mp: 167-169 °C, yield 90%, $[\alpha]_D$ = +26.2 (c = 0.979). Analysis (calcd., found): C (76.62, 76.65), H (8.16, 8.38), N (3.44, 3.51). ESI-MS: $[MH]^+$ = 408; HRMS (m/z): $[M^+]$ calcd. for $C_{26}H_{33}NO_3$, 407.24604; found, 407.24600.

^1H NMR (250 MHz, $CDCl_3$): δ 0.79 (s, 3H, 18-H_3); 3.07 (m, 1H, 16-H); 3.50 (d, ^3J = 6.6 Hz, 1H, 17-H); 3.75 (s, 3H, OCH_3); 3.97 (d, ^3J = 13.9 Hz, 1H, -CH_2); 4,09 (d, ^3J = 13.9 Hz, 1H, -CH_2); 6.61-7.18 (m, 7H, 7x Ar-H).

^{13}C NMR (63 MHz, $CDCl_3$): δ 12.11 (C-18), 51.51 (N-CH_2), 63.55 (C-16), 88.17 (C-17), 116.52, 119.17, 128.18, 128.76 (C_6H_4); 122.77 (H_2C-$\underline{C}_6H_4$); 158.12 (HO-$\underline{C}_6H_4$).

IR (ATR): 1608 (m), 1582 and 1577 (m-w, sh), 1487 (s, arom. C=C).

17β-hydroxy-16α-[*(E)*-5-Chloro-2-hydroxyphenylcarbaldimino]-3-methoxy-estra-1,3,5(10)-triene **7r**

$C_{26}H_{30}NO_3Cl$ (439.99). This compound was not isolated.

17β-hydroxy-16α-[5-Chloro-2-hydroxyphenyl-methylamino]-3-methoxy-estra-1,3,5(10)-triene **7s**

Procedure 3. $C_{26}H_{32}NO_3Cl$ (441.99), mp: 153.5-155 °C (EtOH/water), white amorphous powder, yield 29%, TLC: R_f = 0.14 (uniform).

ESI-MS: $[MH]^+$ = 442.3 (100%).

^{1}H NMR (250 MHz, $CDCl_3$): δ 0.81 (s, 3H, 18-H_3), 1.25-1.65 (m, overl., 6H, aliph. H), 1.7-1.95 (m, overl., 3 H. aliph. H), 2.29 (m_c, overl., 2 H, aliph. H), 2.85 (s, broad, 2H, 6-H_2), 3.08 (t, ^3J = 7.5 Hz, 1H, 16β-H), 3.52 (d, ^3J = 7.5 Hz, 1H, 17α-H), 3.78 (s, 3H, 3-OCH_3), 4.02 (q, ^2J = 15.0 Hz, 2H, 6-H_2), 6.64 (s, 1H, 4-H), 6.68-6.83 (m, overl., 2H, 2-H), 6.99 (s, 1H, 1-H), 7.10 (dd, ^3J = 7.5 Hz, ^4J = 2.5 Hz, 1H, Ar-H), 7.20 (s, ^3J = 7.5 Hz, 1H, Ar-H).

^{13}C NMR (63 MHz, $CDCl_3$): δ 12.10, 25.91, 27.18, 29.66, 30.84, 36.50, 38.33, 43.83, 43.95, 48.14, 51.01, 55.20, 63.39, 88.10, 111.53, 113.82, 117.79, 123.64, 124.05, 126.21, 127.95, 128.50, 132.20, 137.77, 156.80, 157.51.

IR (ATR): 1607 (m), 1588 (m-w), 1577 (w), and 1482 (s, arom. C=C).

17β-hydroxy-16β-[*(E)*-2-hydroxyphenylcarbaldimino]-3-methoxy-estra-1,3,5(10)-triene **8b**

$C_{26}H_{31}NO_3$ (405,56), mp: 221-224 °C (from AcOEt/heptane or MeOH), yield 75%, bright yellow needles and columns.

Analysis (calcd., found): C (76.99, 77.01), H (7.71, 7.71), N (3.47, 3.75); $[\alpha]_D$ = +47.4.

^{1}H NMR (250 MHz, $CDCl_3$): δ 0.90 (s, 3H, 18-H_3), 1.13-2.31 (m, overl., 12H), 2.79 (m, 2H, 6-H_2), 3.66 (t, ^3J = 8.5 Hz, 1H, 17α-H), 3.70 (s, 3H, 3-CH_3O), 3.84 (m, 1H, 16α-H), 6.55-7.25 (m, overl., 7H, 1-H, 2-H, 4-H and 4 x Ar-H), 8.30 (s, 1H, N=CH).

^{13}C NMR (63 MHz, $CDCl_3$): δ 12.1 (C-18), 34.4 (C-15), 37.1 (C-12), 43.4 (C-13), 47.9 (C-14), 69.0 (C-16), 55.5 (OCH_3), 82.9 (C-17), 166.3 (N=CH).

IR (ATR): 1624 (m-s, C=N); 1608 (m-s), 1577 (m-w), and 1497 (s, arom. C=C).

16β-(2-hydroxyphenyl-methylamino)-17β-hydroxy-3-methoxy-estra-1,3,5(10)-triene **8c**

Procedure 2. $C_{26}H_{33}NO_3$ (407.60), mp: 163-167 °C, yield 88%, $[\alpha]_D$ = +97.7 (c = 0.909).

Analysis (calcd., found): C (76.62, 75.91), H (8.16, 8.32), N (3.44, 3.50). ESI-MS: $[MH]^+$= 408.

^{1}H NMR (250 MHz, $CDCl_3$): δ 0.85 (s, 3H, 18-H_3); 3.20 (m, 1H, 16-H); 3.71 (d, 1H, 17-H); 3.76 (s, 3H, OCH_3); 3.93 (q, 2H, -CH_2); 6.61-7.20 (m, 7H, 7 x Ar-H).

^{13}C NMR (63 MHz, $CDCl_3$): δ 12.39 (C-18), 52.04 (N-CH_2), 57.62 (C-16), 80.92 (C-17), 116.30, 119.10, 128.26, 128.74 (C_6H_4); 123.26 (H_2C-$\underline{C}_6H_4$); 157.94 (HO-$\underline{C}_6H_4$).

IR (ATR): 1610 (m), 1590 (m), 1576 (m), and 1496 (m-s, arom. C=C).

3,17β-dihydroxy-16α-*(E)*-phenylcarbaldimino-estra-1,3,5(10)-triene **9b**

Procedure 1. $C_{25}H_{29}NO_3$ (391.51), mp: 235-238 °C (from MeOH), yellow crystals, yield 75%, $[\alpha]_D$ = -2.80 (c = 9.432, pyridine), TLC: R_f = 0.34.

Analysis (calcd., found): C (76.69, 76.36), H (7.47, 7.18), N (3.58, 3.56).

^{1}H NMR (250 MHz, $CDCl_3$): δ 0.89 (s, 3H, 18-H_3), 3.62-3.66 (1H, 16β-H), 3.71 (d, 1H, 17α-H), 6.55-7.32 (Ar-H), 8.33 (s, 1H, CH=N), 13.65 (s, 1H, 3-OH).

^{13}C NMR (63 MHz, $CDCl_3$): δ 12.2 (C-18), 32.8 (C-15), 36.5 (C-12), 44.0 (C-13), 48.4 (C-14), 74.6 (C-16), 163.9 (CH=N).

IR (ATR): 1624 (s, sh, overl.; C=N and C=C), 1615 (m, sh), 1551 (m), and 1497 (m, arom. C=C).

3,17β-dihydroxy-16α-(2-hydroxyphenyl-methylamino)-estra-1,3,5(10)-triene **9c**

Procedure 2. $C_{25}H_{31}NO_3$ (393.52), mp: 144-145 °C (from MeOH, colourless small rods), yield 65%, TLC: R_f = 0.03 (uniform).

ESI-MS: $[MH]^+$ = 394.1 (100), HRMS (m/z): $[M^+]$ calcd. for $C_{25}H_{32}NO_3$, 394.238218; found, 394.238360.

^{1}H NMR (250 MHz, $CDCL_3$ + DMSO-D_6): δ 0.706 (s, 3H, 18-H_3), 1.288-1.863 (m, overl., aliph. H), 2.678 (s, broad, 2H, 6-H_2), 2.998 (d, ^3J = 6.75 Hz, 1H, 17α-H), 3.415 (d, ^3J = 6.75 Hz, 1H, 16β-H), 3.862 (d, ^2J = 13.8 Hz, 1H, N-benzyl-H), 3.948 (d, ^2J = 13.8 Hz, 1H, N-

benzyl-H), 4.199 (s, very broad, X-H), 6.452 (s, 1H, 4-H), 6.503 (d, ^3J = 8 Hz, 1H, 2-H), 6.635-6.715 (m, overl., 2H, 2x Ar-H), 6.947-7.033 (m, overl., 3 H, 3 x Ar-H).

^{13}C NMR ((250 MHz, $CDCL_3$ + DMSO-D_6): δ 11.67, 25.44, 26.65, 28.96, 29.81, 36.12, 37.87, 43.20, 43.29, 47.46, 50.09, 63.18, 86.19, 112.36, 114.74, 115.59, 118.35, 122.44, 123.49, 128.04, 130.35, 137.03, 154.38, 157.39.

IR (ATR): 3270 (m-w, very broad, O-H/N-H); 1624 (m), 1614 (m, sh), 1551 (m), and 1497 (m, arom. C=C).

16α-[*(E)*-2-hydroxyphenyl-carbaldimino]-17β-hydroxy-3-methoxy-13α-estra-1,3,5(10)-triene **10b**

$C_{26}H_{31}NO_3$ (405.24). This compound was not isolated.

16α-(2-hydroxyphenyl-methylamino)-17β-hydroxy-3-methoxy-13α-estra-1,3,5(10)-triene **10c**

Procedure 3. $C_{26}H_{33}NO_3$ (407.25), mp: 131-136 °C (from MeOH), white amorphous powder, yield 70%, TLC: R_f = 0.09.

ESI-MS: M^+ = 407 (100%).

^1H NMR (250 MHz, $CDCl_3$): δ 1.15 (s, 3H, 18-H_3), 1.20-1.70 (m, overl., 8H, 8 x aliph. H), 2.01 (m. overl. 2H, 2x aliph. H), 2.37 (m, 2H, 2x aliph. H), 2.77 (s, broad, 2H, 6-H_2), 3.16 (m, 1H, 16β-H), 3.59 (d, ^3J = 7.0 Hz, 1H, 17β-H), 3.77 (s, 3H, 3-CH_3O), 4.05 (s, 2H, N-benzyl-H_2), 6.59 (d, J = 2.6 Hz, 1H, 4-H), 6.71-6.86 (3H, 3 x Ar-H), 7.01 (d, J = 7.2 Hz, 1H, Ar-H), 7.14 (d, overl., ^3J = 7.3 Hz, 2H, 2x Ar-H).

^{13}C NMR (63 MHz, $CDCl_3$): δ 158.06, 157.22, 137.73, 133.51, 128.76, 128.07, 127.55, 123.00, 119.14, 116.48, 113.16, 112.07, 87.08, 64.10, 59.77, 55.18, 51.67, 49.23, 42.47, 41.18, 38.08, 32.63, 30.26, 29.09, 28.61, 27.46.

IR (ATR): 1610 (m), 1589 (m-w), 1574 (w), and 1497 (m, arom. C=C).

16β-(2-hydroxyphenyl-carbaldimino)-17β-hydroxy-3-methoxy-13α-estra-1,3,5(10)-triene **11b**

$C_{26}H_{31}NO_3$ (405.24). This compound was not isolated.

16β-[*(E)*-2-hydroxyphenyl-methylamino]-17β-hydroxy-3-methoxy-13α-estra-1,3,5(10)-triene **11c**

Procedure 3. $C_{26}H_{33}NO_3$ (407.25), mp: 92-98 °C (from MeOH), white amorphous powder, yield 60%, TLC: R_f = 0.03.

ESI-MS: M^+ = 407 (100%).

^{1}H NMR (250 MHz, $CDCl_3$): δ 0.95 (s, 3H, 18-H_3), 1.20-1.70 (m, overl., 8H, 8 x aliph. H), 2.00-2.50 (m. overl. 3H, 3 x aliph. H), 2.76 (s, broad, 2H, 6-H_2), 3.30 (m, 1H, 16α-H), 3.77 (s, broad, 4H, 3-CH_3O and 17α-H)), 4.01 (d, ^2J = 8.0 Hz, 2H, N-benzyl-H_2), 6.59 (s, 1H, 4-H), 6.73-6.85 (m, overl., 3H, 3 x Ar-H), 6.99 (d, ^3J = 9.0 Hz, 1H, Ar-H), 7.16 (d, overl., ^3J = 9.0 Hz, 2H, 2x Ar-H).

^{13}C NMR (63 MHz, $CDCl_3$): δ 158.06, 157.22, 137.73, 133.51, 128.76, 128.07, 127.55, 123.00, 119.14, 116.48, 113.16, 112.07, 87.08, 64.10, 59.77, 55.18, 51.67, 49.23, 42.47, 41.18, 38.08, 32.63, 30.26, 29.09, 28.61, 27.46.

IR (ATR): 1609 (m), 1590 (m-w), 1577 (w), and 1499 (m-s, arom. C=C).

2β,3β-dihydroxy-1α-[*(E)*-2-hydroxyphenyl-carbaldimino]-cholane **12b**

$C_{34}H_{53}NO_3$ (523.81). Procedure 1: yellow oil (yield 100%), further processing of crude product following Procedure 3 is advantageous.

2β,3β-dihydroxy-1α-(2-hydroxyphenyl-methylamino)-cholane **12c**

Procedure 3. $C_{34}H_{55}NO_3$ (525.81), raw product: clearing point 162-164 °C, amorphous white powder, yield 84%, TLC: R_f = 0.16 (uniform).

ESI-MS: $[MH]^+$ = 526.4 (100%)

^{1}H NMR (250 MHz, $CDCl_3$): δ 0.670 (s, 3H, 18-H_3), 2.861 (s, broad, 1H), 3.863 (d, ^2J = 13.4 Hz, 1H, benzyl-H), 3.900 (m, overl., 1H), 4.086 (d, ^2J = 13.4 Hz, 1H, benzyl-H), 4.221 (s, broad, 1H), 6.774-7.194 (m, 4H, 4 x Ar-H).

^{13}C NMR (63 MHz, $CDCl_3$): δ 12.11, 14.40, 18.73, 21.10, 22.54, 22.79, 23.79, 24.20, 27.99, 28.15, 28.43, 31.41, 32.55, 34.86, 35.77, 36.12, 39.10, 39.24, 39.46, 39.49, 42.58, 48.81, 52.68, 56.05, 56.23, 64.48, 68.81, 70.77, 116.41, 119.34, 122.95, 128.33, 129.10, 157.67.

IR (ATR): 1617 (w), 1588 (m) and 1490 (m, arom. C=C); 2864-2928 (broad, s, C-H), and 3364 (m, very broad, OH and NH).

3β-hydroxy-2α[*(E)*--2-hydroxyphenyl-carbaldimino]-cholane **13b**

Procedure 1 (in MeOH at 40 °C). $C_{34}H_{53}NO_2$ (507.81). Cooling the reaction mixture to -18 °C furnishes a pale yellow precipitate, after drying: clearing point 196 °C, yield 90%, TLC: R_f = 0.76 (uniform). This product was further used.

3β-hydroxy-2α-(2-hydroxyphenyl-methylamino)-cholane **13c**

Procedure 2 (solvent THF/EtOH = 1:1 v/v). $C_{34}H_{55}NO_2$ (509.81), clearing point 167-168 °C (from MeOH), fine white crystals, yield 53%, TLC: R_f = 0.60 (uniform).

ESI-MS: $[MH]^+$ = 510.4 (100%); HRMS (m/z): $[M^+]$ calcd. for $C_{34}H_{56}NO_2$, 510.43110; found, 510.43232.

^1H NMR (250 MHz, $CDCl_3$): δ 0.665 (s, 3H, 18-H_3), 2.633 (m, 1H), 3.432 (m, 1H), 3.958 (d, ^2J = 13.0 Hz, 1H, benzyl-H), 4.100 (d, ^2J = 13.0 Hz, 1H, benzyl-H), 6.780-7.165 (m, 4H, 4 x Ar-H).

^{13}C NMR (63 MHz, $CDCl_3$): δ 11.50, 13.20, 18.68, 21.32, 22.53, 22.77, 23.83, 24.19, 27.99, 28.10, 28.20, 31.89, 35.02, 35.77, 36.18, 36.73, 37.31, 39.53, 39.89, 42.59, 44.72, 50.30, 54.26, 56.27, 56.37, 59.64, 116.48, 119.03, 123.55, 127.94, 128.61, 158.10.

IR (ATR): 3070 (m, very broad, N-H/O-H); 2932 (s, broad), 2894 (m-s, broad) and 2852 (m-s, broad, C-H); 1607 (w, sh), 1598 (w,sh) and 1507 (w-m, arom. C=C).

3α-hydroxy-2α-[*(E)*-2-hydroxyphenyl-carbaldimino]-cholane **14b**

Procedure 1 (in EtOH at 50 °C). $C_{34}H_{53}NO_2$ (507.81). Cooling the reaction mixture to 4 °C furnishes a pale yellow product in quantitative yield which was directly further used.

3α-hydroxy-2α-(2-hydroxyphenyl-methylamino)-3α-hydroxycholane **14c**

Procedure 2 (or 3). $C_{34}H_{55}NO_2$ (509.81), clearing point: 285-290 °C (decomposition; from EtOH/water, then AcOEt/hexane), glassy compound, yield 73%, TLC: R_f = 0.22.

ESI-MS: M^+ = 509.4 (100%)

^{1}H NMR (250 MHz, $CDCl_3$): δ 0.537 (s, 3H, 18-H_3), 0.620 (s, 3H, 19-H_3); 0.742, 0.7967 and 0.803: overl., 21-, 26- and 27-H_3), 0.7 - 2.0 (m, overl., aliph. H), 2.478 (s, 1H, 2β-H), 3.010 (s, 1H, 3β-H), 4.057 and 4.138 (each: s, broad, 1H; CH_2-N), 6.737 (t, ^3J = 8 Hz, 1H, Ar-H), 6.924 (d, ^3J = 8 Hz, 1H, Ar-H), 7.108 (t, ^3J = 8 Hz, 1H, Ar-H), 7.256 (d, ^3J = 8 Hz, 1H, Ar-H), 8.366 (s, broad, 1H, OH), 8.495 (s, broad, 1H, OH).

^{13}C NMR (63 MHz, $CDCl_3$): δ 17.01, 17.08, 20.44, 27.08, 28.40, 28.82, 32.33, 32.61, 32.86, 36.41, 39.63, 39.70, 39.95, 40.37, 40.78, 41.31, 42.86, 44.84, 47.21, 48.99, 58.60, 60.80, 60.97, 61.49, 62.55, 68.21, 120.76, 122.59, 124.40, 135.44, 136.30, 160.95.

IR (ATR): 1599 (broad, overl., m), 1503 (w), and 1460 (m-s, arom. C=C); 2898-2930 (broad, s, C-H), 3074, and 3300 (m, very broad, OH and NH).

3. STS 948

$C_{32}H_{42}N_4O$ (498.72). The "mixed" tridentate ligand **STS 948** is accessible via reductive amination of estrone methylether with 3-(imidazole-1-yl)propylamine and subsequent acylation with 2-pyridyl acetic acid followed by reduction with borane as described in ref. 1.

4. Non-steroid-derived analogs (series 15-20)

These compounds were prepared as described above for steroids (section 2, general procedures 1-3), whereby the condensation step was performed under reflux conditions. Preferably, the one-pot approach (general procedure 3) was chosen.

(E)-2-[(cyclopentylimino)methyl]phenol **15b** [34]

$C_{12}H_{15}NO$ (189.25). This compound was not isolated.

2-[(cyclopentylamino)methyl]phenol hydroperchlorate **15c**

Procedure 3. $C_{12}H_{18}NO_5Cl$ (291.73), mp: 168-169 °C (from EtOH/water), white amorphous powder, yield 78%, TLC (AcOEt:THF, 1:1 v/v): R_f = 0.66 (uniform).

Analysis (calcd., found): C (49.41, 49.42), H (6.22, 6.18), N (4.80, 4.91). ESI-MS: $[M-ClO_4]^+$ = 192.0 (100), HRMS (m/z): $[M^+]$ calcd. for $C_{12}H_{18}NO$, 192.138839; found, 192.138590.

^1H NMR (250 MHz, acetone-D_6): δ 1.55-2.00 (m, overl., 6H, 3 x CH_2), 2.10-2.30 (m, 2H, CH_2), 3.84 (m_c, 1H, CH), 4.39 (s, 2H, CH_2-N), 6.88 (t, ^3J = 7.5 Hz, 1H, Ar-H), 6.99 (d, ^3J = 7.5 Hz, 1H, Ar-H), 7.27 (td, ^3J = 7.9 Hz, ^4J = 1.6 Hz, 1H, Ar-H), 7.41 (d, ^3J = 7.5 Hz, 1H, Ar-H).

^{13}C NMR (63 MHz, acetone-D_6): δ 23.77, 46.56, 59.67, 115.29, 117.63, 120.01, 131.11, 131.64, 155.58.

IR (ATR): 1061 (s, broad); 1183, 1243, 1259, 1344, 1405, 1464, 1506, 1599 (all: m); 2973 (m-w), 3080 (m), 3180 (m-w), and 3438 (m, broad).

(E)-2-[(cyclohexylimino)methyl]phenol **16b** [35]

$C_{13}H_{17}NO$ (203.28). This compound was not isolated.

2-[(cyclohexylamino)methyl]phenol hydroperchlorate **16c**

Procedure 3. $C_{13}H_{20}NO_5Cl$ (305.75), mp: 152-153 °C (from EtOH/water), colorless plates, yield 59%, TLC (AcOEt:THF, 1:1 v/v): R_f = 0.75 (uniform).

Analysis (calcd., found): C (51.07, 51.42), H (6.59, 6.57), N (4.58, 4.73). ESI-MS: $[M-ClO_4]^+$ = 206.2 (100), HRMS (m/z): $[M^+]$ calcd. for $C_{13}H_{20}NO$, 206.154489; found, 206.154840.

^1H NMR (400 MHz, acetone-D_6): δ 1.27 (m_c, 1H, CH), 1.41 (m_c, 2H, CH_2), 1.66 (ddd, ^2J = 24.2 Hz, ^3J = 12.2 Hz, ^4J = 3.4 Hz, 3H, 3 x CH), 1.89 (m_c, 2H, 2x CH), 2.35 (m_c, 2H, 2x CH), 3.44 (s, 1H, CH), 4.49 (s, 2H, CH_2-N), 6.92 (td, ^3J = 7.5 Hz, ^4J = 0.9 Hz, 1H, Ar-H), 7.02 (d, ^3J

= 7.7 Hz, 1H, Ar-H), 7.31 (td, ^3J = 8.1 Hz, ^4J = 1.6 Hz, 1H, Ar-H), 7.45 (dd, ^3J = 7.5 Hz, ^4J = 1.4 Hz, 1H, Ar-H), 7.81 (s, broad, 2H, NH_2^+), 9.25 (s, 1H, OH).

^{13}C NMR (101 MHz, acetone-D_6): δ 24.20, 24.76, 45.03, 58.00, 115.38, 117.79, 120.16, 131.17, 131.71, 155.83.

IR (ATR): 1029 (vs, broad), 1095 (s, broad), 1347 (m), 1436 (m), 1453(s-m), 1506 (m), 1602 (m-w), 2861 (w), 2929 (m), 3080 (m-w), 3180 (m), and 3420 (m, broad).

The preparation of the free base 2-[(cyclohexylamino)methyl]phenol is described in ref. 36.

(E)-2-[(cyclooctylimino)methyl]phenol **17b** [37]

$C_{15}H_{21}NO$ (231.33). This compound was not isolated.

2-[(cycloctylamino)methyl]phenol hydroperchlorate **17c**

Procedure 3. $C_{15}H_{24}NO_5Cl$ (333.81), mp: 136-138.5 °C (from EtOH/water), colorless plates, yield 33%, TLC (AcOEt:THF, 1:1 v/v): R_f = 0.75 (uniform).

Analysis (calcd., found): C (53.97, 54.14), H (7.25, 7.20), N (4.20, 4.28). ESI-MS: $[M-ClO_4]^+$ = 234.0 (100), HRMS (m/z): $[M^+]$ calcd. for $C_{15}H_{24}NO$, 234.185789; found, 234.185730.

^1H NMR (400 MHz, acetone-D_6): δ 1.50-1.71 (m, overl., 8H, 4 x CH_2), 1.82-1.90 (m, 2H, CH_2), 1.96-2.08 (m, 2H, CH_2), 2.21-2.27 (m, 2H, CH_2), 3.69 (s, 1H, CH), 4.49 (s, 2H, CH_2-N), 6.92 (td, ^3J = 7.5 Hz, ^4J = 0.9 Hz, 1H, Ar-H), 7.02 (dd, ^3J = 8.1 Hz, ^4J = 0.5 Hz, 1H, Ar-H), 7.31 (td, ^3J = 8.0 Hz, ^4J = 1.6 Hz, 1H, Ar-H), 7.45 (dd, ^3J = 7.5 Hz, ^4J = 1.5 Hz, 1H, Ar-H), 7.75 (s, broad, 2H, NH_2^+), 9.28 (s, 1H, OH).

^{13}C NMR (101 MHz, acetone-D_6): δ 23.63, 25.52, 26.02, 45.39, 59.50, 115.38, 117.82, 120.19, 131.20, 131.77, 155.73.

IR (ATR): 1044 (vs, sh, broad), 1102 (s, sh, overl.), 1184 (m), 1237 (m-w), 1262 (m), 1340 (m9, 1411 (m, sh), 1424 (m, sh), 1463 (m, sh), 1506 (m-w>), 1598 (m-w), 1613 (m-w), 2858 (m-w), 2919 (m-s), 3106 (m-w), 3192 (m), and 3412 (m-s, broad).

E)-2-[(1-adamantylamino)methyl]phenol **18b** [38]

$C_{17}H_{21}NO$ (255.34). This compound was not isolated.

2-[(1-adamantylamino)methyl]phenol hydroperchlorate **18c**

$C_{17}H_{24}NO_5Cl$ (357.83). Modified procedure 3: A mixture of 5.0 mmol (938.5 mg) of 1-adamantylamine hydrochloride, 5.1 mmol (516.2 mg) of triethylamine, and 10 mmol (1.22 g) of salicylaldehyde in 40 ml of abs. EtOH was refluxed for 45 min. After cooling to room temperature, 15 mmol (567.4 mg) of sodium borohydride was added. The mixture decolorized within 20 min and was stirred for further 20 min at room temperature, neutralized with concentrated acetic acid, and concentrated until a thick precipitate appears. To this suspension, an excess of water was added followed by concentrated perchloric acid (final pH: 2). After stirring for 10 minutes, the obtained precipitate was filtered off, thoroughly washed twice with water, and recrystallized from 50 ml of EtOH/water (1:4 v/v) to yield a colorless crystal wadding. Yield after drying: 1.5 g (84%), mp: 228-229.5 °C (decomp., rods), TLC (AcOEt:THF, 1:1 v/v): R_f = 0.82 (uniform).

Analysis (calcd., found): C (57.06, 57.29), H (6.76, 6.71), N (3.91, 4.01). ESI-MS: $[M-HClO_4]^+$ = 258.3 (100), HRMS (m/z): $[M^+]$ calcd. for $C_{17}H_{24}NO$, 258.185789; found, 258.185540.

^1H NMR (300 MHz, acetone-D_6): δ 1.76 (m_c, overl., 6H, 3 x CH_2), 2.20 (s, overl., 6H, 3 x CH_2), 2.34 (s, overl., 3H, 3 x CH), 3.22 (s, broad, 2H, NH_2^+), 4.35 (s, 2H, CH_2-N), 6.84 (td, ^3J = 7.5 Hz, ^4J = 1.2 Hz, 1H, Ar-H), 6.69 (d, ^3J = 7.5 Hz, 1H, Ar-H), 7.25 (td, ^3J = 8.1 Hz, ^4J = 1.8 Hz, 1H, Ar-H), 7.40 (dd, ^3J = 7.5 Hz, ^4J = 1.2 Hz, 1H, Ar-H).

^{13}C NMR (75 MHz, acetone-D_6): δ 35.02, 35.23, 35.44, 37.78, 38.01, 38.22, 40.11, 58.68, 115.24, 117.96, 119.97, 131.05, 131.77, 155.82.

IR (ATR): 1036, 1069, and 1082 (vs, broad, overl.); 1123 (s, sh), 1263 (m), 1308 (m), 1339 (m, sh), 1369 (m), 1424 (m, sh), 1457 (m-s), 1506 (m-w), 1581 (m-w), 2856 (m, sh), 2915 (s, sh), 3100 (m-w), 3159 (m), and 3432 (m-w, broad).

Recently, the free base was prepared by reduction of **18b** with $NaCNBH_3$ in 76% yield. [39]

(E)-2-[(phenylimino)methyl]phenol **19b** [40,41]

$C_{13}H_{11}NO$ (197.23). This compound was not isolated.

2-[(phenylamino)methyl]phenol hydroperchlorate **19c**

Procedure 3. $C_{13}H_{14}NO_5Cl$ (299.71), mp: 183-186 °C (from water), colorless plates, yield 93%, TLC (AcOEt:THF, 1:1 v/v): R_f = 0.78 (uniform).

Analysis (calcd., found): C (52.10, 52.29), H (4.71, 4.67), N (4.67, 4.69). ESI-MS: $[M\text{-}HClO_4]^+$ = 200.0 (100), HRMS (m/z): $[M^+]$ calcd. for $C_{13}H_{14}NO$, 200.107539; found, 200.107290.

^{1}H NMR (400 MHz, acetone-D_6): δ 4.88 (s, 2H, CH_2-N), 6.86 (td, ^3J = 7.5 Hz, ^4J = 0.9 Hz, 1H, Ar-H), 7.05 (d, ^3J = 7.6 Hz, 1H, Ar-H), 7.31 (td, ^3J = 7.6 Hz, ^4J = 1.8 Hz, 2H, 2x Ar-H), 7.52-7.61 (m, overl., 3H, 3 x Ar-H), 7.66-7.71 (m, 2H, 2x Ar-H), 7.90 (s, broad, 1H, XH).

^{13}C NMR (101 MHz, acetone-D_6): δ 53.34, 115.37, 116.86, 120.11, 123.32, 130.03, 130.17, 131.56, 132.04, 135.01, 156.01.

IR (ATR): 1025 (vs, br, overl.), 1087 (s, br, overl.), 1162 (m), 1242 (m), 1262 (m), 1347 (m, sh), 1408 (m-s), 1461 (m-s), 1493 (m), 1507 (m-w), 1588 (m), 1598 (m), 3100 (m, v broad), and 3400 (m, v broad).

(E)-2-[(1-pyrenylimino)methyl]phenol **20b** [42,43]

$C_{23}H_{15}NO$ (321.38). This compound was prepared as described in ref. 44.

Crude product: mp: 146-150 °C, yellow crystals, yield 81%, TLC: R_f = 0.80 (uniform).

Analysis (calcd., found): C (85.96, 86.23), H (4.70, 4.64), N (4.36, 4.44).

^{1}H NMR (250 MHz, $CDCl_3$): only one CH=N signal at δ = 8.819 ppm (s, 1H).

IR (ATR): 1607 (m, CH=N).

Using heptane/THF (9:1) instead of EtOH as solvent gave the same results.

2-[(1-pyrenylamino)methyl]phenol **20c**

Procedure 3. $C_{23}H_{17}NO$ (323.40), mp: 160-163 °C (from EtOH), citreous plates, yield 73%, TLC (AcOEt:THF, 1:1 v/v): R_f = 0.58 (uniform).

Analysis (calcd., found): C (84.42, 85.45), H (5.30, 5.23), N (4.33, 4.39). ESI-MS: $[M]^+$ = 322.8 (100), HRMS (m/z): $[M^+]$ calcd. for $C_{23}H_{17}NO$, 323.131014; found, 323.130670.

^1H NMR (250 MHz, CD_3CN): δ 4.72 (s, 2H, CH_2-N), 6.00 (s, 1H, X-H), 6.75-6.97 (m, 2H, 2x Ar-H), 7.12 (t, ^3J = 7.7 Hz, 1H, Ar-H), 7.30-7.42 (m, 2H, 2x Ar-H), 7.52 (s, 1H, Ar-H), 7.79 (d, ^3J = 8.9 Hz, 1H, Ar-H), 7.84-8.12 (m, overl., 6H, 6x Ar-H), 8.20 (d, ^3J = 9.3 Hz, 1H, Ar-H).

^{13}C NMR (63 MHz, CD_3CN): δ 44.20, 110.63, 116.19, 118.22 (overl.), 120.82, 121.25, 123.70, 123.85, 124.06, 124.53, 126.02, 126.22, 126.40, 126.60, 127.03, 127.28, 128.62, 129.15, 129.67, 132.65, 133.26, 143.76, 156.17.

IR (ATR): 835 (vs), 1243 (s), 1490 <8m<9, 1507 (m), 1587 (m-s, sh), 2855 and 3033 (m, v broad, overl.), and 3292 (m); no CH=N signal at 1607 cm^{-1}.

2-[(1-pyrenylimino)methyl]naphth-3-ol **20n**

Procedure 1 (solvent THF:EtOH, 1:1 v/v). $C_{27}H_{17}NO$ (371.44). The uniform (TLC) crude product was further processed. Yield 92%, orange-ochre finely crystalline powder. Analytical sample from THF/EtOH (1:1 v/v, 69% recovery): orange-ochre plates, mp: 216-219.5 °C (decomp.), TLC (AcOEt:THF, 1:1 v/v): R_f = 0.64 (uniform).

Analysis (calcd., found): C (87.31, 87.17), H (4.61, 4.55), N (3.77, 3.83). ESI-MS: $[M]^+$ = 371.9 (100), HRMS (m/z): $[M^+]$ calcd. for $C_{27}H_{18}NO$, 372.138839; found, 372.139050.

^1H NMR (400 MHz, DMSO-D_6), 2.4:1 mixture of E/Z isomers according to integrals of N = CH signals at 9.41 ppm (E-isomer) and 10.41 ppm (Z-isomer): δ 7.34-7.46 (m, overl., ca. 2H, 2x Ar-H), 7.57 (s, broad, ca. 1H, Ar-H), 7.80-7.94 (m, ca. 2H, 2x Ar-H), 8.00 (d, J = 6.6 Hz, ca. 2H, 2x Ar-H), 8.08-8.15 (m, ca. 1H, Ar-H), 8.16-8.28 (m, ca. 2H, 2x Ar-H), 8.28-8.38 (m, ca. 2H, 2x Ar-H), 8.42 (d, broad, ^3J = 7.9 Hz, 1H, Ar-H), 8.49-8.55 (m, 2H, 2x Ar-H), 8.49 (s, 0.7H, CH=N), 10.41 (s, 0.3H, CH=N), 12.68 (s, 1H, OH).

^{13}C NMR (101 MHz, $CDCl_3$): δ 110.94, 113.71, 116.77, 122.33, 122.83, 124.23, 125.83, 126.06, 126.47, 126.47, 126.63, 127.07, 127.65, 127.75, 127.85, 128.14, 128.54, 129.05, 129.28, 129.80, 130.15, 130.34, 131.25, 131.55, 132.97, 134.68, 136.54, 142.79, 156.12, 156.59, 164.29, 193.11.

IR (ATR): 828 (s), 840 (s), 1145 (m), 1310 (m, sh), 1470 (m, sh), 1507 (m-s), 1540 (m-w), 1559 (m), 1654 (w), 1690 (w), and 3080 (w, v broad).

2-[(1-pyrenylamino)methyl]naphth-3-ol **20o**

Procedure 2 (suspension of low soluble crude product **20n** in THF:EtOH, 2:3 v/v, which turns to a clear solution after some minutes). $C_{27}H_{19}NO$ (373.45), mp: 234-236 °C (decomp.; from EtOH:THF, 1:1 v/v), ochre plates, which exhibit in solution pronounced solvatochromic effects. Yield 81%, TLC (AcOEt:THF, 1:1 v/v): R_f = 0.44 (uniform).

Analysis (calcd., found): C (86.84, 86.75), H (5.13, 5.07), N (3.75, 3.79). ESI-MS: $[MH]^+$ = 373.8 (100), 138.9 (87); HRMS (m/z): $[M^+]$ calcd. for $C_{27}H_{20}NO$, 374.154489; found, 374.154560.

^{1}H NMR (400 MHz, DMSO-D_6): δ 4.79 (d, J = 3.9 Hz, 2H, CH_2-N), 7.01 (s, broad, overl., 1H, X-H), 7.11 (m_c, 1H, Ar-H), 7.20 (d, ^3J = 8.7 Hz, 2H, 2x Ar-H), 7.26 (d, ^3J = 7.5 Hz, 1H, Ar-H), 7.49 (t, ^3J = 8.6 Hz, 1H, Ar-H), 7.54-8.02 (m, overl., 9H, 9 x Ar-H), 8.39 (t, ^3J = 9.5 Hz, 1H, Ar-H), 9.85 (s, 1H, X-H).

^{13}C NMR (101 MHz, $CDCl_3$): δ 43.42, 109.06, 109.32, 116.36, 121.37, 122.06, 122.64, 122.95, 123.45, 125.19, 125.62, 125.78, 125.99, 126.12, 126.75, 127.54, 127.95, 128.11, 128.44, 131.87, 132.42, 134.02, 143.46, 154.30.

IR (ATR): 825 (s, sh), 1301 (m), 1308 (m), 1507 (m), ca. 2840 and 3048 (m-w, v broad), and 3282 (m-s); no signals at 1654 and 1690 cm^{-1}.

Critical issues and future perspectives on synthesis

1. In order to address the problem of potential hormone side action, variations at the steroid skeleton and the introduction of a second (or third) hydroxyarylmethylamino group are of interest (undermining the lock/key principle).

13α-steroids [7] possessing a cis-hydrindane system and represented here by series **10** and **11** are promising candidates in this context. The starting 17-ketone is simple to prepare via isomerization of estrone-3-methyl ether as outlined above in the synthesis schemes for **10a** and **11a**.

The absence of hormonal activity is also supposed for derivatives of 1-hydroxy-4-methyl-estratriene-17-one, also simple to prepare via isomerization of 1,4-androstadiene-3,17-dione (ADD). [12-14] The synthesis and biological investigation of 16 and 17-(hydroxyarylmethyl)amines from these should give further information about the influence of the aromatic A ring on biological activity.

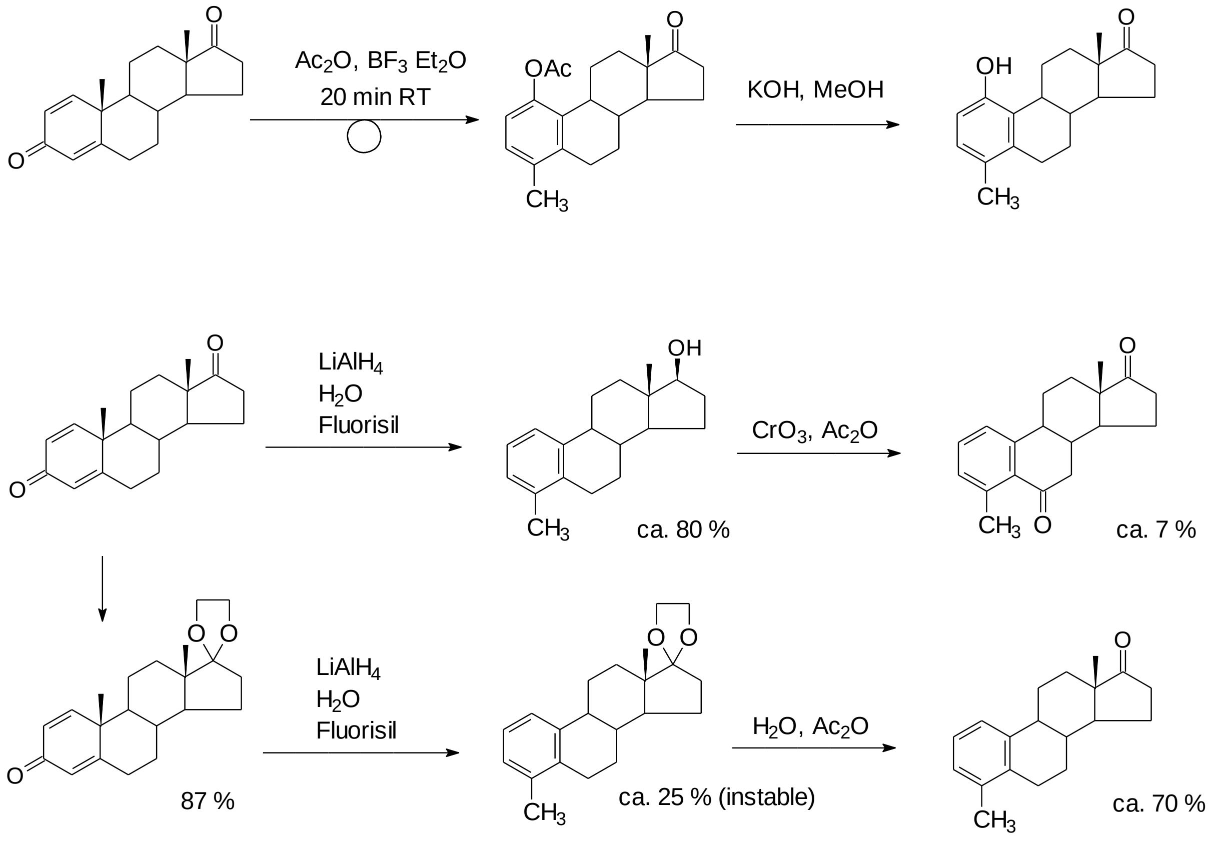

Furthermore, the synthesis of compounds with the (hydroxyarylmethyl)amino group in other positions of the estratriene core seems expedient. Recently a two-step method for the introduction of a 12β-hydroxy group starting with estratriene-17-ketones was developed. [2]

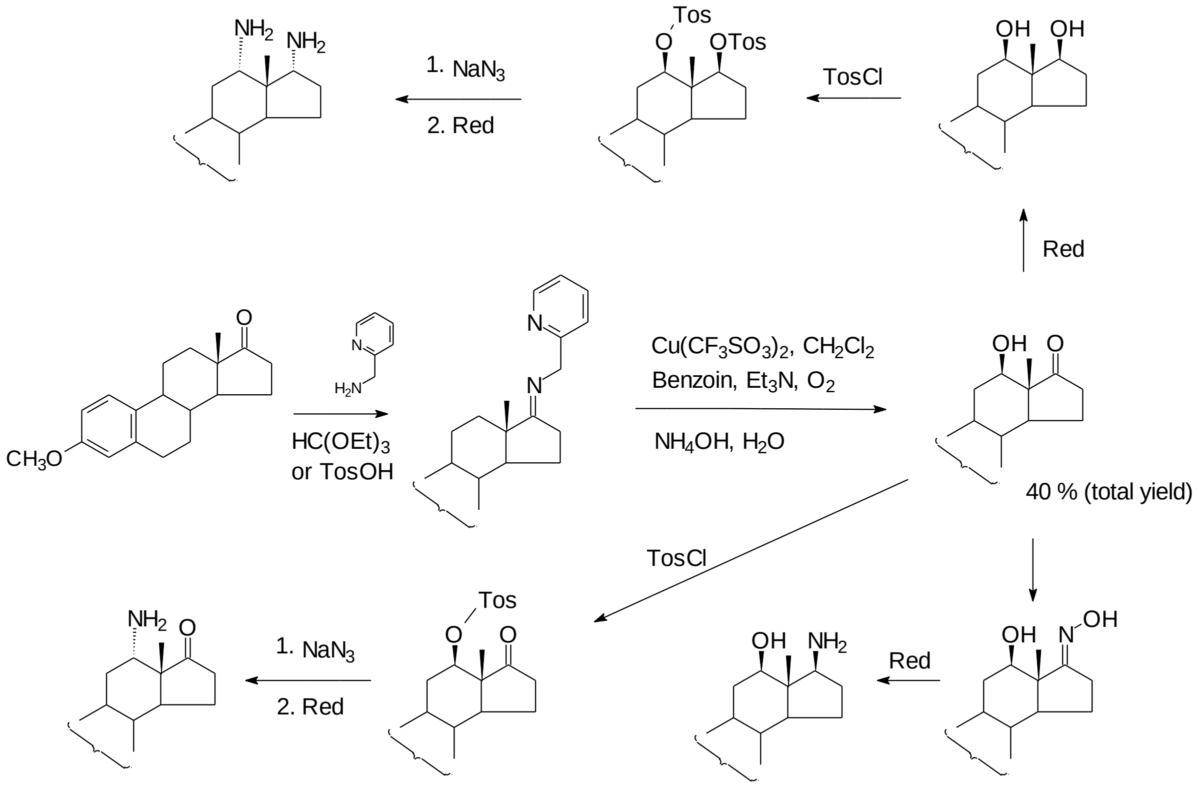

This opens the way for the synthesis of hitherto barely investigated 12-substituted estratrienes. Introducing two (hydroxybenzyl)amino groups in positions 12 and 17 also seems possible. Is there a cumulative or additive biological effect?

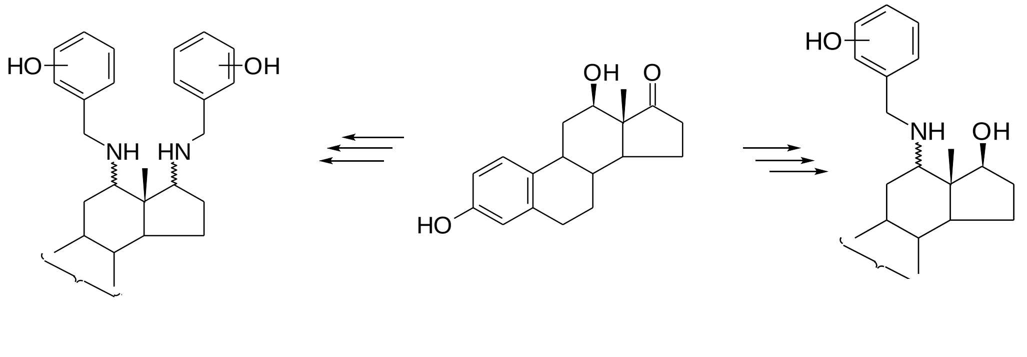

In contrast to antimalarials with the hydroxyarylmethyl-amino groups attached at steroid ring atoms in a defined steric alignment, it would also be of interest to investigate compounds

bearing this group in a more flexible environment. Therefore estrone derivatives can be transformed into ω-bromoalkylethers with different chain lengths. [15] Substitution of the bromine group with azide, subsequent azide reduction followed by aldehyde-condensation with the amine, and azomethine reduction should furnish hydroxyarylmethyl amines with high conformational freedom.

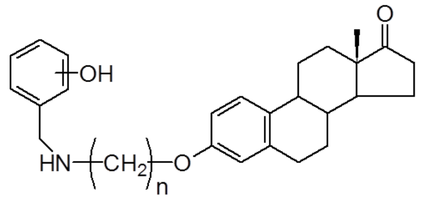

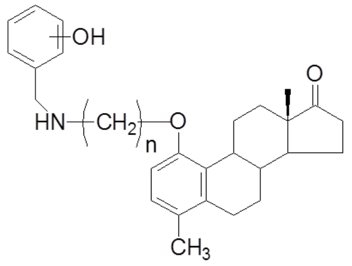

Interestingly some related ω-pyridinium alkylethers of steroidal phenols exhibit potent antibacterial and antiproliferative activities. [15]

It also stands to reason to replace the steroid gonan skeleton with derivatives of synthetic nonsteroidal “hormones” - in the first instance with estrogen mimetica such as biphenol A (BPA, 2,2-*bis*(4-hydroxyphenyl)propane)) or diethylstilbestrol (4,4'-(3*E*)-hex-3-ene-3,4-diyldiphenol). In contrast to steroids, such compounds are easily accessible and modifiable from cheap starting materials.

2. Ongoing optimization of the hydroxyarylmethyl amino core:

The formation of quinone methides has been revealed as the basic principle of a large number of chemical and biological processes. [16] Their reactivity is mainly due to their electrophilic nature, which can be correlated to their toxicological properties. [17] The electrophilicity is tuneable with substituents. Excellent *in vitro* activities of the chlorinated derivatives **1s** and **2s** (Table 1, main document) suggest ongoing investigation of different substitution patterns, especially those with electron-withdrawing groups such as halogens. Halogens can also serve as leaving groups. Therefore halogen methyl as well as allyl, hydroxymethyl, and trialkylammoniummethyl substituents are of special interest and may

also lead to highly reactive quinone methides via different activation modes. This was demonstrated in more detail, e.g. in connection with DNA-based damaging processes: (1) *metal-mediated oxidative Fenton*-like transformation of allyl phenols, e.g. eugenol,[18] (2) *thermal and photochemical oxidation* of 2-hydroxybenzyl-trimethylammonium salts,[17] (3) *bio- and photo-reductive* activation, e.g. of mesyloxymethyl- and bromomethyl-naphthoquinone derivatives.[19] Additionally, preparation of steroid-linked tandem quinone methide precursors [20,21] or chimeric derivatives bearing further pharmacophores would be interesting. Promising examples are hydroxyarylmethylamino steroids with a quinine moiety as leaving group via quaternary quinine-ammonium salt attachment (as described recently for a quinine-based chemo sensor in another context [22]) or via the reaction of steroid amino alcohols with DAMNACANTHAL and subsequent reduction of the azomethine.

Working hypothesis for drug design - further arguments

Role of the steroid moiety:

The capability of steroids to deliver small molecules into living cells via non-specific (e.g. via serum albumin) and specific (via receptors) binding modes is well known and the basis for applications such as fluorochrome-based receptor binding assays. Therefore, the specificity of receptor binding of the steroid-fluorochrome conjugate is crucially dependent upon the kind of steroid substitution, and maintenance of the key/lock principle is irrevocable. This is investigated in detail for a series of oestrogen derivatives. In conclusion, “free” and sterically unhindered 3- and 17β-hydroxy steroid binding sites are demanded. [23-26]

We assume that the steroid moiety acts mainly as a carrier with zero or repressed hormonal activity via non-specific steroid binding sites. So far, this is supported by series **15** to **20**, representing lipophilic 2-hydroxyphenylmethylamino derivatives of mono- and polycyclic aliphatic and aromatic hydrocarbons. In order to ensure that the steroid motive is acting as a specific interacting group rather than just as a general lipophilicity-permitting group, which

could increase potency through entropic factors, the synthesis of further variants with smaller other lipophilic substituents in place of the steroid is of interest. Interestingly, within the cycloaliphatic non-steroidal series **15**-**18** in the following order, a weak antimalarial activity may be deduced (in brackets IC_{50}-values in nM, cf. Table SI2a): cyclopentyl (> 6,000) ≈ cyclohexyl (> 6,000) < 1-adamantyl (4,000) < cyclooctyl (2,000). The adamanthylamine derivative **18c** is closely related to the neuroprotective agent *memantine* (3,5-dimethyladamantan-1-amine).[44]

Chemistry and role of postulated quinone methide intermediates:

Quinone methides are involved as transient intermediates in a wide range of biological processes such as storage, defence, or antibiotic activity and are currently emerging as useful intermediates in organic synthesis.[16] The high reactivity is due to their propensity to undergo rapid re-aromatization either via *Michael* addition of nucleophiles (“cross linking”) [1], or, in the absence of suitable nucleophiles or at high concentration, “dimerization” or “polymerization” (typically in the presence of parent aromatics).

This behaviour is the basis of action of “dye-substrates” for detecting peroxidatic activity. The influence of substitution patterns on staining capability at the cellular level was investigated recently in detail,[2] and the outcome supports our hypothesis compiled in Scheme 4. In conclusion, an almost alkylating reaction mode with little or no oxidant activity is hypothesized, as ascertained from a large number of drugs.[16-19,27,28]

1) In the presence of suitable substituents, intramolecular cycloaddition may also occur, either with 2π-electron partners to benzoxans or via oxa-6π-electro-cyclization to benzopyrans.[16]

2) (1) Oxidative self-anchoring fluorescent dyes following a “hetero-cross-linking pathway.[29-31]

(2) Chromogenic precipitating substrates following a poly-condensation (so called "polymerization") pathway, which is tuneable by utilizing metal catalytic effects via introduction of metal chelating groups.[32-33]

Supplementary Note 2 - Effects of compound 1o on the cytosolic redox potential of *P. falciparum* 3D7 parasites using the genetically encoded redox sensor hGrx1-roGFP2

In order to further study and substantiate the proposed mechanism of action of the steroid compounds, compound **1o** was tested in cell culture on the chloroquine-sensitive *P. falciparum* 3D7 strain transiently expressing the cytosolic glutathione redox sensor hGrx1-roGFP2 generated in our laboratory.[45] To differentiate between pharmacologic effects of compound **1o** on the glutathione homeostasis of $3D7^{[hGrx1\text{-}roGFP2]}$ parasites and direct interactions of the compound with the probe (potentially causing artificial effects), its *in vitro* interaction with the recombinant protein hGrx1-roGFP2[45] was characterized first. Heterologous overexpression of hGrx1-roGFP2 and *in vitro* measurements with the recombinant protein were carried out as described[45] with minor modifications. Stock solutions of diamide (DIA) and dithiothreitol (DTT) to fully oxidize and to fully reduce the probe, respectively, were dissolved in sterile distilled H_2O, while compound **1o** was dissolved in DMSO. Compound **1o** and the redox-active compounds DIA and DTT were diluted with a degassed (1 h, RT) standard reaction buffer (100 mM potassium phosphate, 1 mM EDTA, pH 7.0) and used immediately. Purified recombinant hGrx1-roGFP2 protein was reduced with 20 mM DTT for 30 min at 4 °C, desalinated (Zeba™ Spin Desalting Columns, Thermo Scientific), and diluted in reaction buffer to a final concentration of 1.25 µM. A 5-fold drug/redox-active compound dilution (10 µl) was mixed with 40 µl of 1.25 µM hGrx1-roGFP2 in a 96-well microplate (black, half-area, Greiner bio-one, Frickenhausen). The emission of hGrx1-roGFP2 (510 nm) after excitation at 405 nm and 475 nm was measured in a plate reader (Clariostar, BMG Labtech) with optimal read settings. The ratio of the fluorescence

signals at 405/475 nm were calculated and plotted against time and/or concentration of compound **1o** or redox active compounds. Data from three independent experiments were analyzed. No direct interaction was detected for compound **1o** within the range of 10 nM and 100 µM for 4 h and 24 h incubations. Transfection of the *Pf*3D7 strain with the redox sensor was carried out, and the parasites were cultured as described under constant drug pressure with 5 nM WR99210 (Jacobus Pharmaceuticals, New Jersey, USA).[45,46] *P. falciparum* trophozoites were enriched via magnet separation.[47] Parasitemia was counted by using Giemsa-stained blood smears. The IC_{50} of compound **1o** on *P. falciparum* 3D7 asexual blood stages was verified to be about 5 nM in an additional 72 h [^{3}H] incorporation assay[45] directly before the experiment. The effect of compound **1o** on *P. falciparum* was investigated in 4 h and 24 h incubations. For 4 h experiments, trophozoite stage parasites (26-30 h post invasion) of 3D7$^{[hGrx1\text{-}roGFP2]}$ (6-8% parasitemia) were magnetically enriched (Miltenyi Biotec, Germany), counted by using the improved Neubauer hemocytometer (Brand GmbH, Germany), and returned to cell culture (at 0.5 x 10^6 trophozoites/µl) for at least 1 h to recover. 1.0 x 10^6 cells in 100 µl cell culture medium were placed into LoBind tubes (Eppendorf) for 4 h incubation experiments. The parasites were treated with compound **1o** at 50 x, and 100 x IC_{50} and with mefloquine (MQ) as a standard antimalarial drug at 1 µM (IC_{50} = 8 nM)[45] for 4 h under cell culture conditions and subsequently blocked with 2 mM N-ethylmaleimide (NEM) for 15 min at 37 °C. For 24 h experiments, a 5 ml culture (6-8% parasitemia) of ring stage parasites (6-10 h post invasion) was treated with compound **1o** at 4 x, 10 x, 20 x IC_{50} and with 100 nM MQ. Prior to enrichment, cysteines were blocked with 2 mM NEM. For 4 h and 24 h experiments, cells were washed after incubation with pre-warmed Ringer's solution (122.5 mM NaCl, 5.4 mM KCl, 1.2 mM $CaCl_2$, 0.8 mM $MgCl_2$, 11 mM D-glucose, 25 mM Hepes, 1 mM NaH_2PO_4, pH 7.4) and were seeded onto poly-L-lysine-coated µ-slides VI (Ibidi, Martinsried, Germany). A Leica confocal system TCS SP5 inverted microscope equipped with the objective HCX PL APO 63.0x1.30 GLYC 37 °C UV connected to a 37 °C temperature chamber was used. The argon laser power was set to 20%; scanning was performed at 400 Hz frequency and at a 512 × 512 pixel resolution. The smart gain and

smart offset were 950 V and -0.9%, respectively. With a sequential scan, we excited the sensor at 405 nm and at 488 nm and detected emissions at 500-550 nm. Laser intensity for both lines was adjusted to match the full dynamic range of the probe to the dynamic range of the detector (405 nm: 12%, 488 nm: 4%). Autofluorescence images were simultaneously taken at ex 405 nm / em 430-450 nm and individually defined together with the background for every image, but no fluorescence signal could be detected. All experiments included non-treated parasites as controls, and fully oxidized and fully reduced parasites with 1 mM DIA and 10 mM DTT (2 min incubation), respectively, prior to blocking with NEM. Only parasites showing fluorescent signals at both 405 and 488 nm excitation and an intact host cell were chosen for analysis. Each experiment was carried out three times. At least 10 parasites were analyzed, resulting in at least 30 experimental values per incubation. The Leica LAS AF Lite software for fluorescence analysis was used. The 405/488 nm ratio was calculated. The obtained ratio values were normalized to the control ratio value, which was set to 100. The graphs were plotted using the GraphPad Prism 5 software (San Diego, CA, USA). One way-ANOVA test with 95% confidence intervals with the Dunnett's Multiple Comparison Test was applied for statistical analysis of significance (*, $p < 0.05$; **, $p < 0.01$; ***, $p < 0.001$).

Supplementary Figure 8 panel A shows an approximately 1.5-fold oxidizing effect of compound **1o** for both 250 nM (50 x IC_{50}) and 500 nM (100 x IC_{50}) on $3D7^{[hGrx1\text{-}roGFP2]}$-enriched parasites after 4 h of incubation, which is comparable to the result of the treatment with 1 µM MQ. 24 h incubation (Supplementary Figure 8 panel B) with compound **1o** resulted in a higher increase of the fluorescence ratio in a concentration-dependent manner. Interestingly, 20 nM of compound **1o** (4 x IC_{50}) exhibited a greater oxidation of the hGrx1-roGFP2 redox probe (1.8-fold) than the incubation with the antimalarial drug MQ at 100 nM (1.5-fold). In comparison, 100 nM of compound **1o** (20 x IC_{50}) led to a 2.4-fold change in fluorescence ratio, indicating a higher impact of the compound on the GSH/GSSG level in the parasite cytosol than MQ. Although the results failed to reach significance in the experimental setup chosen, they indicate a trend. Therefore, the influence of compound **1o** on the glutathione redox homeostasis deserves to be studied in more detail.

Supplementary Note 3 – Characterization of haem-adducts

To further substantiate the covalent character of the haem-adducts observed with **1o** (pseudomolecular mass m/z = 1,057.7) or **2o** (pseudomolecular mass m/z = 1,057.7), we carried out another set of experiments in which the solutions containing **1o** (or **2o**) and haem at equimolar concentrations (50 µM) were acidified at very low pH (pH < 1) with an excess of TFA. Under these conditions and assuming non-covalent interactions, no haem-adduct formation should be seen due to the unfavorable conditions. Supplementary Figure 12 first confirms the data already obtained for the 1:1 stoichiometric mixture of **1o** and haem at pH 7-8, demonstrating the formation of a haem-adduct at *m/z* 1,057.7 associated with a high DV_{50}. Importantly, lowering the pH to a value below 1 with an excess of TFA (expressed as pure TFA/ml of solution) does not lead to fading of the peak associated with haem-**1o** species, but on the contrary it favors its formation, thus substantiating its covalent nature. The same conclusions can be drawn for compound **2o** with haem (Supplementary Figure 12).

To go further, we performed the same experiments on a steroid derivative (designated as **STS948**, see Figure 1 in the manuscript) that displays a steroid moiety together with nitrogen-based units that are known to bind haem. In contrast to **1o** and **2o**, this compound is clearly not prone to quinone methide formation and according to our hypothesis would not lead to covalent drug-haem adducts. At pH 7.5 and using absorption spectrophotometric titrations, **STS948** was shown to bind haem with a K_D (0.8 µM) larger than those measured with **1o** (3.3 µM) or **2o** (8.3 µM) (Supplementary Fig. 14). CID ESI-MS conducted at pH 7-8 is additional evidence of the presence of a **STS948**:haem adduct associated to a DV_{50} of 367 V along with 36% of complex remaining at a fragmentor voltage of 400 V (Supplementary Fig. 15). At a first sight, these data would suggest a more active antiplasmodial derivative based on the data already obtained for **1o** and **2o** and therefore invalidate our initial hypotheses. However, the antiplasmodial activity IC_{50} on *Plasmodium falciparum* 3D7 strain was found to be 675 nM, far behind the nM antiplasmodial activities measured for **1o** and **2o**.

To get a deeper insight into the haem binding process, we performed the same set of MS experiments under classical conditions (CH_3CN/H_2O + 0.1% formic acid pH 7-8, (Supplementary Fig. 16) and under acidic conditions. If the first experiments carried out at neutral pH corroborate the formation of **STS948**:haem adduct at *m/z* 1,128.75, those conducted under acidic conditions reveal the absence of any complexation between **STS948** and haem. These data clearly confirmed the expected non-covalent character chairing between **STS948** and haem and give credibility to our assumptions on covalent complexes with **1o** and **2o**.

Supplementary Note 4 – Considerations on metal and haem binding *in vivo*

Both compounds were shown to lead to 1:1 stoichiometric complexes with stability constants log K_{LM} (L = **1o** or **2o**, M = Cu(II) or Fe(III)) ranging from = 4.1 to 4.7. These constants can be considered rather low. However, one should keep in mind that red blood cells (RBCs) represent a haemoglobin-rich (and iron-rich) environment. It is usually assumed that for healthy RBCs the haemoglobin mass concentrations are at about 12-18 grams dl^{-1}, which corresponds to haemoglobin concentrations of about 10 mM. It is also accepted that the erythrocytic forms of *Plasmodium* degrade about 60% to 80% of the total RBC Hb content as a vital source of amino acids.[48] As a consequence, parasitized RBCs contain high amounts of haem and free iron. We demonstrated that compound **1o** displays good stability in plasma (62% and 58% remaining, respectively, after a 3 h incubation with human and mouse plasma) with, however, poor aqueous solubility (<1 µg/ml across a range of pH; < 2.3×10^{-6} M). Assuming a concentration of 10^{-8} M of **1o** (100 times lower than the aqueous solubility) and 7×10^{-4} M Fe (corresponding to 10% of the degraded Hb in the form of free Fe), one can estimate that more than 80% of **1o**:Fe complex will be formed (Supplementary Fig. 18). In the case of haem complexes with **1o** (K_D ~ µM), much higher amounts of the haem:**1o** adduct will be formed. This rather simple calculation demonstrates that despite a rather low stability of the metal complexes with **1o**, a high percentage of these complexes is expected *in vivo* due to the particular conditions in *Plasmodium falciparum*-infected RBCs.

Supplementary Methods

Physicochemistry – General Methods

Distilled water was purified by passing it through a mixed bed of ion exchanger (Bioblock Scientific R3-83002, M3-83006) and activated carbon (Bioblock Scientific ORC-83005). Dimethyl-sulphoxide DMSO (E. Merck, Uvasol, for spectroscopy or Sigma, Bioreagent, for molecular biology), acetonitrile CH_3CN (E. Merck Uvasol, for spectroscopy), chloroquine (Sigma, diphosphate salt), and amodiaquine (Sigma, dihydrochloride dihydrate) were obtained from commercial suppliers and used without further purification. Copper(II) perchlorate ($Cu(ClO_4)_2 \cdot 6H_2O$, Fluka, purum p.a.) and Fe(III) perchlorate ($Fe(ClO_4)_3 \cdot xH_2O$, Aldrich) are commercial products that were used without further purification. The Cu(II) content of the stock solution was determined according to the classic colorimetric titrations,[49] while that of the Fe(III) stock solution was determined spectrophotometrically.[50] CAUTION! Perchlorate salts combined with organic ligands are potentially explosive and should be handled in small quantities and with adequate precautions.[51] Hepes (2-[4-(2-hydroxyethyl)piperazin-1-yl]ethanesulphonic acid, Gerbu Biotechnik) buffer (0.2 M) was prepared in water, and the pH (7.5) was adjusted with NaOH slurry. The pH reading was done on a PHM240 MeterLab® millivoltmeter fitted with a combined glass electrode (Metrohm 6.0234.100, Long Life, 0.1 m NaCl). Calibration was done with commercial Merck® INIST-certified buffers (1.68, 4.00, 6.86, 7.41, and 9.18). Haematin (Fe^{III}PPIX(OH)) solution (5 mM) was prepared from haemin equine Type III (Fe^{III}PPIXCl, Sigma-Aldrich) and 50% aqueous ammonia (ESI-MS CID experiments) and was vigorously stirred at room temperature (RT) for 1 h. Haematin (Fe^{III}PPIX(OH)) solution (2 mM) was prepared from haemin equine Type III (Fe^{III}PPIXCl, Sigma-Aldrich) and 0.1 M aqueous NaOH (absorption spectrophotometric titrations) and was vigorously stirred at room temperature (RT) for 1 h. Stock solutions (5 mM) of the substrates (**1o**, **2o**, **1a**, **2a**, **1h,** and **2h**) for the different assays were freshly prepared in Eppendorf tubes in pure acetonitrile. All the stock solutions were prepared by weighing solid products using a Mettler Toledo XA105 Dual Range (0.01/0.1 mg

- 41/120 g), and complete dissolution was achieved using an ultrasonic bath. All solutions were protected from daylight to avoid any photochemical degradation. All the physiochemical measurements were carried out at 25.0(2) °C.

Spectrophotometric titration of haematin π-π dimer with the substrates

Typically, an aliquot of 38 µL of the Fe^{III}PPIX(OH) stock solution (~ 2 mM) was dissolved in 2.5 ml 0.2 M sodium Hepes buffer at pH 7.5 in a 1 cm Hellma optical quartz cell. The absorption spectrophotometric titrations were carried out by adding microvolumes of a stock solution of the substrate (~5 mM in CH_3CN for **1a**, **1i**, **1o**, **2o**). Aliquots of 2 µl of the stock substrate solution were successively added to the reaction mixture, and after each addition a UV-visible absorption spectrum (250 nm < λ < 800 nm) was recorded with an Agilent Cary 5000 spectrophotometer. Special care was taken to ensure that equilibrium was attained after each addition of the substrate. The dissociation constants K_D and the stoichiometry of the species at equilibrium were determined by processing the spectrophotometric data with the Specfit program.[52-57] Specfit uses factor analysis to reduce the absorbance matrix and to extract the eigenvalues prior to the multiwavelength fit of the reduced data set according to the Marquardt algorithm. The uncertainties on the log K values are given as 3σ with σ = standard deviation. Origin 7.0 was used to process the analytical results.[58]

Electrospray mass spectrometric measurements stopping point

Electrospray mass spectra of the haem complexes were obtained with an Agilent Technologies 6120 quadrupole equipped with an electrospray (ESI) interface. For electrospray ionization, the drying gas was heated to 250 °C, and its flow was set at 6 $l.min^{-1}$. The capillary exit voltage was fixed at 5 kV, and the skimmer voltage varied from 50 to 200 V in order to optimize the signal responses. Scanning was performed from m/z = 100 to 1,500. CID experiments were performed on the Fe^{III}PPIX/substrate complex with a capillary exit (cone voltage) ranging from 120 to 400 V with 20 V increments.[59] Stock solution of haematin ($[Fe^{III}PPIX(OH_2)]^{3+}$ or $[Fe^{III}PPIX(OH)]^{2+}$) was freshly prepared from haemin

(ferriprotoporphyrin chloride, $[Fe^{III}PPIX(Cl)]^{2+}$) just before use in 50% ammonia. A stock solution of chloroquine (**CQ**, 2 mM) was prepared in water, while those of (~**1a**, **1i**, **1o**, **2o** were prepared in CH_3CN (5 mM). Haematin and the substrates were mixed together in CH_3CN/H_2O (50:50 *v:v*) in order to obtain equimolar concentrations of 100 µM. Prior to analyses, the samples were further diluted at 50 µM in $CH_3CN/H_2O/HCO_2H$ (50:50:1 *v:v:v*). The sample solutions were then introduced into the spectrometer source with a syringe pump (Kd Scientific) with a flow rate of 800 $\mu l.h^{-1}$.

Spectrophotometric titration of 1o or 2o by Cu(II) and Fe(III)

Typically, an aliquot of 250 µl (or 500 µl) of a stock solution (~ 0.89 mM) of **1o** or **2o** was dissolved in 2.5 (or 2.0 ml) of a mixed solvent made of 0.2 M sodium hepes buffer at pH 7.5 and DMSO (1:1 v/v) in a 1 cm Hellma optical quartz cell. The absorption spectrophotometric titrations were carried out by adding microvolumes of a stock solution of the metallic cation (5 mM for Cu(II) or 7.36 mM for Fe(III). Aliquots of 5 µl of the stock metal solution were successively added to the reaction mixture, and after each addition a UV-visible absorption spectrum (250 nm < λ < 800 nm) was recorded with an Agilent Cary 5000 spectrophotometer. Special care was taken to ensure that equilibrium was attained after each addition of the substrate. The association constants $K_{L.M}$ (**L** = **1o**, **2o** and M = Fe(III) or Cu(II)) and the stoichiometry of the species at equilibrium were determined by processing the spectrophotometric data with the Specfit program. The uncertainties on the log *K* values are given as 3σ with σ = standard deviation. Origin 7.0 was used to process the analytical results.[58]

Supplementary References

1. Gonschior, M., Kötteritzsch, M., Rost, M., Schönecker, B. & Wyrwa, R. Synthesis of N,N-bis[2-(2-pyridyl)ethyl]amino steroids and related compounds intended as chiral ligand for copper ions. *Tetrahedron Asymmetry* **11**, 2159-2182 (2000).

2. Schönecker, B., Zheldakova, T., Lange, C., Günther, W., Görls, H. & Bohl, M. Intramolecular γ-hydroxylations of nonactivated C-H bonds with copper complexes and molecular oxygen. *Chem. Eur. J.* **10**, 6029-6042 (2004).

3. Krieg, R., Wyrwa, R., Möllmann, U., Görls, H. & Schönecker, B. Novel (N-ferrocenylmethyl)amines and (N-ferrocenylmethylene)imines derived from vicinal steroid amino alcohols and amines: synthesis, molecular structure, and biological activity. *Steroids* **63**, 531-541 (1998).

4. Schönecker, B. & Ponsold, K. & Steroide XL. 16,17-Azido- und 16,17-Aminoalkohole des Östra-1,3,5(10)-trien-3-methylethers. *Tetrahedron* **31**, 1113-1118 (1975).

5. Ponsold, K., Schlegel, J., Schönecker, B. & Schubert, G. 16α-Heterosubstituted estradiols (estriol analogues). Preparation and binding power at receptors of rat uterus *in vitro*. *Pharmazie* **30**, 32-34 (1975).

6. Heiman, D.F., Senderoff, S.G., Katzenellenbogen, J.A. & Neeley, R.J. Estrogen receptor-based imaging agents. 1. Synthesis and receptor binding affinity of some aromatic and D-ring halogenated estrogens. *J. Med. Chem.* **23**, 994–1002 (1980).

7. Schönecker, B., Lange, C., Kötteritzsch, M., Günther, W., Weston, J., Anders, E. & Görls, H. Conformational Design for 13α-Steroids. *J. Org. Chem.* **65**, 5487-5497 (2000).

8. Mernyak, E., Schönecker, B., Lange, C., Kötteritzsch, M., Görls, H., Wölfling, J. & Schneider, G. Addition reactions at the 16(17) double bond of 3-methoxy-13-estra-1,3,5(10),16-tetraene. *Steroids* **68**, 289-296 (2003).

9. Ponsold, K. Stickstoffhaltige Steroide IV. Darstellung von Azidohydrinen und vicinalen Aminoalkoholen aus Epoxysterinen. *Chem. Ber.* **96**, 1855-1864 (1963).

10. Dubs, M., Krieg, R., Görls, H. & Schönecker, B. Reactions of the four diastereomeric 16-amino-17-hydroxy-3-methoxyestra-1,3,5(10)-trienes with aromatic ortho-hydroxy and heteroaromatic α-aldehydes and with 1,3-dicarbonyl compounds - molecular structures of condensation products and of copper(II) complexes. *Steroids* **65**, 305-318 (2000).

11. Narasimhan, N.S. & Mali, R.S. Synthetic application of lithiation reactions-VII: New syntheses of linear and angular naphthofurans and benzocoumarins**.** *Tetrahedron* **31**, 1005-1009 (1975).

12. Hanson, J.R., Raines, D. & Knights, S.G. The preparation and dienone-phenol rearrangement of androsta-2,5-diene-4,17-dione. *J. Chem. Soc.*, Perkin Trans. 1: Organic and Bio-Organic Chemistry, **1980**, 1311-1313.

13. Caspi, E., Piatak, D.M. & Grover, P.K. Steroids containing ring A aromatic. Part XI. Mechanism of the dienol–benzene rearrangement. *J. Chem. Soc.* Section C, Organic, **1966**, 1034-1037.

14. Gentles, M.J., Moss, J.B., Herzog, H.L. & Hershberg, E.B. The dienol-benzene rearrangement. Some chemistry of 1,4-androstadiene-3,17-dione. *J. Amer. Chem. Soc.* **80**, 3702-3704 (1958).

15. Lange, C., Holzhey, N., Schönecker, B., Beckert, R., Möllman, U. & Dahse, H.-M. Omega-pyridiniumalkylethers of steroidal phenols: new compounds with potent antibacterial and antiproliferative activities. *Bioorg. Med. Chem.* **12**, 3357-3362 (2004).

16. Willis, N.J. & Bray, C.D. *ortho*-Quinone Methides in Natural Product Synthesis. *Chem. Eur. J.* **18**, 9160–9173 (2012).

17. Modica, E., Zanaletti, R., Frecdero, M. & Mella, M. Alkylation of amino acids and glutathione in water by o-quinone methide, reactivity and selectivity. *J. Org. Chem.* **66**, 41-52 (2001).

18. Bodell, W.J., Ye, Q., Pathak, D.N. & Pongracz, K. Oxidation of eugenol to form DNA adducts and 8-hydroxy-2'-deoxyguanosine: role of quinone methide derivative in DAN adduct formation. *Carcinogenesis* **19**, 437-443 (1998).

19. Chatterjee, M. & Rokita, S. The role of quinone methide in the sequence-specific alkylation of DNA. *J. Amer. Chem. Soc.* **116**, 1690-1697 (1994).

20. Zeng, L., Wang, P., Zhang, H., Zhuang, X., Dai, Q. & Liu, W. Highly selective and sensitive heparin probing from supramolecular assembly of pyrene derivatives. O*rg. Lett.* **11**, 4294-4297 (2009).

21. Wang, P., Liu, R., Wu, X., Ma, H., Cao, X., Zhou , P., Zhang , J., Weng , X., Zhang , X.-L., Qi , J., Zhou , X. & Weng, L. A potent, water-soluble and photoinducible DNA cross-linking agent. *J. Am. Chem. Soc.* **125**, 1116–1117 (2003).

22. Zeng, Q. & Rokita, S.E. Tandem quinone methide generation for cross-linking DNA. *J. Org. Chem.* **61**, 9080-9081 (1996).

23. Asai, D., Tokunaga, T., Kondo, K., Kawaguchi, T., Takayanagi,S., Shinmyozu, T., Nakai, M., Yakabe, Y. & Shimohigashi, Y. Direct measure of fluorescence intensity for efficient receptor-binding assay: conjugates of ethinylcarboxyestradiol and 5 (and 6)-carboxyfluorescein via α,ω-diaminoalkanes as a tracer for estrogen receptor. *J. Biochem.* **143**, 781-792 (2008).

24. Mappus, E., Chambon, C., Fenet, B., de Ravel, M.R., Grenot, C. & Cuilleron, C.Y. Synthesis of (5-azido-2-nitrobenzoyl)amido, (4-azido-2-nitrophenyl)amino, and (5-azido-2-nitro-3,4,6-trifluorophenyl)amino derivatives of 17α-methylamino-, 17α-ethylamino-, and 17α-propylamino-5α-dihydrotestosterone as reagents of different linker lengths for the photoaffinity labeling of sex hormone binding globulins and androgen receptors. *Steroids* **65**, 459–481 (2000).

25. Hauptmann, H., Paulus, B., Kaiser, T. & Luppa, P.B. Concepts for the syntheses of biotinylated steroids. Part II: 17β-estradiol derivatives as immunochemical probes. *Bioconjug. Chem.* **11**, 537–548 (2000).

26. Adamczyk, M., Mattingly, P.G. & Reddy, R.E. Synthesis of 6β-aminoestradiol and its biotin, acridinium, and fluorescein conjugates. *Steroids* **63**, 130-134 (1998).

27. Hulsman, N., Medema, J.P., Bos, C., Jongejan, A., Leurs, R., Smith, M.J., de Esch, I.J.P., Richel, D. & Wijtmans, M. Chemical Insights in the concept of hybrid drugs: the antitumor effect of nitric oxide-donating aspirin involves a quinone methide but not nitric oxide or aspirin. *J. Med. Chem.* **50**, 2424–2431 (2007).

28. Thompson, D.C., Thompson, J.A., Sugumaranc, M. & Moldéusd, P. Biological and toxicological consequences of quinone methide formation. *Chem. Biol. Interact.* **86**, 129-162 (1992).

29. Krieg, R., Eitner, A., Günther, W. & Halbhuber, K.-J. Optimization of heterocyclic 4-hydroxystyryl derivatives for histological localization of endogenous and immunobound peroxidase activity. *Biotech. Histochem.* **82**, 235-262 (2007).

30. Krieg, R., Eitner, A., Günther, W., Schürer, C., Lindenau, J. & Halbhuber, K.-J. . N,N-dialkylaminostyryl dyes: specific and highly fluorescent substrates of peroxidase and their application in histochemistry. *J. Mol. Hist.* **39**, 169-191 (2008).

31. Krieg, R. & Halbhuber, K.-J. Detection of endogenous and immunobound peroxidase - The *status quo* in histochemistry. *Prog. Histochem. Cytochem.* **45**, 81-142 (2010).

32. Krieg, R., Halbhuber, K.-J. & Oehring, H. Novel chromogenic substrates with metal chelating properties for the histochemical detection of peroxidatic activity, derived from 3-amino-9-ethylcarbazle (AEC) and 3,6-diamino-9-ethylcarbazole. *Cell. Mol. Bio.* **46**, 1191-1212 (2000).

33. Krieg, R., Oehring, H. & Halbhuber, K.-J. Towards versatile, chromogenic, and metal-associating substrates for the determination of peroxidatic activity/hydrogen peroxide

by chemically designing Schiff-based derivatives. *Cell. Mol. Bio.* **47**, OL209–OL234 (2001).

34. Giuseppone, N., Schmitt, J.-L., Schwartz, E. & Lehn, J.-M. Scandium(III) Catalysis of transimination reactions. Independent and constitutionally coupled reversible processes. *J. Am. Chem. Soc.* **127**, 5528-5539 (2005).

35. Mierde, H. V., Van Der Voort, P., De Vos, D. & Verpoort, F. A ruthenium-catalyzed approach to the Friedländer quinoline synthesis. *Eur. J. Organ. Chem.* **2008**, 1625-1631 (2008).

36. Kainz, Q. M., Zeltner, M., Rossier, M., Stark, W. J. & Reiser, O. Synthesis of trisubstituted ureas by a multistep sequence utilizing recyclable magnetic reagents and scavengers. *Chemistry - A European Journal* **19**, 10038-10045 (2013).

37. Patterson, A. E., Miller, J. J., Miles, B. A., Stewart, E. L., Melanson, J.-M. E. J., Vogels, C. M., Cockshutt, A. M., Decken, A., Morin, P. Jr. & Westcott, S. A. Synthesis, characterization and anticancer properties of (salicylaldiminato)platinum(II) complexes. *Inorganica Chimica Acta* **415**, 88–94 (2014).

38. Wang, Z., Gao, J., Wang, J., Jin, X., Zou, M., Li, K. & Kang, P. Spectroscopic analyses on interaction of amantadine-salicylaldehyde, amantadine-5-chloro-salicylaldehyde and Amantadine-o-Vanillin Schiff-Bases with bovine serum albumin (BSA). *Spectrochimica Acta Part A: Molecul. Biomolec. Spectroscopy* **83**, 511–517 (2011).

39. Wang, J., Ma, C., Wang, J., Jo, H., Canturk, B., Fiorin, G., Pinto, L. H., Lamb, R. A., Klein, M. L. & DeGrado, W. F. Discovery of novel dual inhibitors of the wild-type and

the most prevalent drug-resistant mutant, S31N, of the M2 proton channel from influenza A virus. *J. Med. Chem.* **56**, 2804–2812 (2013).

40. Tang, Z., Chen, W., Zhu, Z., Liu, H. Synthesis of 2,3-diaryl-3,4-dihydro-2H-1,3 benzoxazines and their fungicidal activities. *J. Heterocycl. Chem.* **48**, 255-260 (2011).

41. İkiz, M., İspir, E., Aytar, E., Ulusoy, M., Karabuğa, S., Aslantaş, M. & Çelik, Ö. Chemical fixation of CO_2 into cyclic carbonates by azo-containing Schiff base metal complexes. *New J. Chem.* **39**, 7786-7796 (2015).

42. Safin, D. A., Bolte, M. & Garcia, Y. Solid-state photochromism and thermochromism of N-salicylidene pyrene derivatives. *Cryst. Eng. Comm.* **16**, 8786-8793 (2014).

43. Kim, H. J., Bhuniya, S., Mahajan, R. K., Puri, R., Liu, H., Ko, K. C., Lee, Y. L. & Kim, J. S. Fluorescence turn-on sensors for HSO_4^-. *Chem. Commun.* **2009**, 7128-7130 (2009).

44. Stuart, J. T. & Grossberg, G. T. Memantine: a review of studies into its safety and efficacy in treating Alzheimer's disease and other dementias. *Clin. Interv. Aging.* **4**, 367-377 (2009).

45. Kasozi, D., Mohring, F., Rahlfs, S., Meyer, A.J., Becker, K. Real-time imaging of the intracellular glutathione redox potential in the malaria parasite *Plasmodium falciparum*. *PLoS Pathog* **9**, e1003782 (2013).

46. Crabb, B. S., Rug, M., Gilberger, T. W., Thompson, J. K., Triglia, T., Maier, A. G., Cowman, A. F. Transfection of the human malaria parasite *Plasmodium falciparum*. *Methods Mol Biol* **270**, 263-276 (2004).

47. Paul, F., Roath, S., Melville, D., Warhurst, D. C., Osisanya, J. O. Separation of malaria-infected erythrocytes from whole blood: use of a selective high-gradient magnetic separation technique. *Lancet* **2**, 70-1 (1981).

48. Francis, S.E., Sullivan, D.J. Jr, Goldberg, D.E. Hemoglobin metabolism in the malaria parasite *Plasmodium falciparum*. *Ann. Rev. Microbiol.* **51**, 97-123 (1997).

49. Méthodes d'Analyses Complexométriques avec les Titriplex®, Ed. Merck, Darmstadt.

50. Bastian, R., Weberling, R. & Palilla, F. Determination of iron and cyanide in cyanoferrate complexes. *Anal. Chem.* **28**, 459 (1956).

51. Raymond, K.N. Tragic consequence with acetonitrile adduct. *Chem. Eng. News* 61, 4 (1983).

52. Gampp, H., Maeder, M., Meyer, C.J. & Zuberbühler, A.D. Calculation of equilibrium constants from multiwavelength spectroscopic data – I: mathematical considerations. *Talanta* **32**, 95-101 (1985).

53. Rossotti, F.J.C., Rossotti, H.S. & Whewell, R.J. The use of electronic computing techniques in the calculation of stability constants. *J. Inorg. Nucl. Chem.* **33**, 2051-2065 (1971).

54. Gampp, H., Maeder, M., Meyer, C.J. & Zuberbühler, A.D. Calculation of equilibrium constants from multiwavelength spectroscopic data – II: SPECFIT: two user-friendly programs in basic and standard FORTRAN 77. *Talanta* **32**, 257-264 (1985).

55. Gampp, H., Maeder, M., Meyer, C.J. & Zuberbühler, A.D. Calculation of equilibrium constants from multiwavelength spectroscopic data – IV: model-free least-squares refinement by use of evolving factor analysis. *Talanta* **33**, 943-951 (1986).

56. Marquardt, D.W. An algorithm for least-squares estimation of nonlinear parameters. *J. Soc. Indust. Appl. Math.* **11**, 431-441 (1963).

57. Maeder, M. & Zuberbühler, A.D. Nonlinear least-squares fitting of multivariate absorption data. *Anal. Chem.* **62**, 2220-2224 (1990).

58. MicrocalTM OriginTM, 2002; Microcal Software, Inc.: Northampton, MA.

59. Mishra, E. *et al*. Erratum to: Axial Imidazole Binding Strengths in Porphyrinoid Cobalt(III) Complexes as Studied by Tandem Mass Spectrometry. *J. Am. Soc. Mass Spectrom.* **23**, 1428-1439 (2012).
